# Toward ^68^Ga and ^64^Cu Positron Emission Tomography Probes: Is H_2_dedpa-*N*,*N*′-pram the Missing Link for dedpa Conjugation?

**DOI:** 10.1021/acs.inorgchem.2c04123

**Published:** 2023-01-20

**Authors:** Celia Pena-Bonhome, Desiree Fiaccabrino, Tamara Rama, Daniel Fernández-Pavón, Lily Southcott, Zhengxing Zhang, Kuo-Shyan Lin, Andrés de Blas, Brian O. Patrick, Paul Schaffer, Chris Orvig, María de Guadalupe Jaraquemada-Peláez, Teresa Rodríguez-Blas

**Affiliations:** † Grupo METMED, Departamento de Química, Universidade da Coruña, Campus da Zapateira s/n, Coruña 15071A, Spain; ‡ Medicinal Inorganic Chemistry Group, Department of Chemistry, University of British Columbia, Vancouver British Columbia V6T 1Z1, Canada; § Life Sciences Division, TRIUMF, 4004 Wesbrook Mall, Vancouver British Columbia V6T 2A3, Canada; ¥ Department of Molecular Oncology, BC Cancer Research Institute, Vancouver, British Columbia V5Z 1L3, Canada; ⊥ Department of Radiology, University of British Columbia, Vancouver, British Columbia V5Z 1M9, Canada; ∥ Department of Chemistry, University of British Columbia, Vancouver British Columbia V6T 1Z1, Canada; ⧧ Department of Chemistry, Simon Fraser University, Burnaby, British Columbia V5A 1S6, Canada

## Abstract

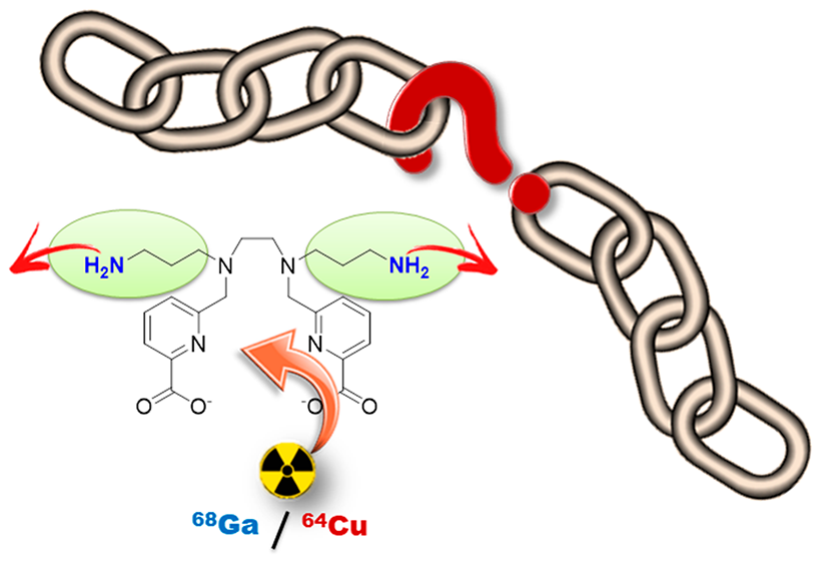

H_2_dedpa-*N*,*N*′-pram (H_2_L^1^), a new chelator derived from the hexadentate ligand 1,2-bis[[(6-carboxypyridin-2-yl)methyl]amino]ethane (H_2_dedpa), which incorporates 3-propylamine chains anchored to the secondary amines of the ethylenediamine core of the latter, has emerged as a very promising scaffold for preparing ^68^Ga- and ^64^Cu-based positron emission tomography probes. This new platform is cost-effective and easy to prepare, and the two pendant primary amines make it versatile for the preparation of bifunctional chelators by conjugation and/or click chemistry. Reported herein, we have also included the related H_2_dedpa-*N*,*N*′-prpta (H_2_L^2^) platform as a simple structural model for its conjugated systems. X-ray crystallography confirmed that the N_4_O_2_ coordination sphere provided by the dedpa^2–^ core is maintained at both Ga(III) and Cu(II). The complex formation equilibria were deeply investigated by a thorough multitechnique approach with potentiometric, NMR spectrometric, and UV–vis spectrophotometric titrations, revealing effective chelation. The thermodynamic stability of the Ga(III) complexes at physiological relevant conditions is slightly higher than that of 1,4,7,10-tetraazacyclododecane-1,4,7,10-tetraacetic acid (DOTA), the common and clinically approved chelator used in the clinic [pGa = 19.5 (dedpa-*N*,*N*′-pram) and 20.8 (dedpa-*N*,*N*′-prpta) versus 18.5 (DOTA) at identical conditions], and significantly higher for the Cu(II) complexes [pCu = 21.96 (dedpa-*N*,*N*′-pram) and 22.8 (dedpa-*N*,*N*′-prpta) versus 16.2 (DOTA)], which are even more stable than that of the parent ligand dedpa^2–^ (pCu = 18.5) and that of 1,4,7-triazacyclononane-1,4,7-triacetic acid (NOTA) (pCu = 18.5). This high stability found for Cu(II) complexes is related to the conversion of the secondary amines of the ethylenediamine core of dedpa^2–^ into tertiary amines, whereby the architecture of the new H_2_L^1^ chelator is doubly optimal in the case of this metal ion: high accessibility of the primary amine groups and their incorporation via the secondary amines, which contributes to a significant increase in the stability of the metal complex. Quantitative labeling of both chelators with both radionuclides ([^68^Ga]Ga^3+^ and [^64^Cu]Cu^2+^) was observed within 15 min at room temperature with concentrations as low as 10^–5^ M. Furthermore, serum stability studies confirmed a high radiochemical *in vitro* stability of all systems and therefore confirmed H_2_L^1^ as a promising and versatile chelator for further radiopharmaceutical *in vivo* studies.

## Abstract 2

H_2_dedpa-*N*,*N*′-pram, which contains primary amino groups attached to the dedpa core, has emerged as a very promising scaffold for preparing both ^68^Ga- and ^64^Cu-based positron emission tomography probes. This new platform is cost-effective and easy to prepare, and the primary amines make it versatile for the preparation of bifunctional chelators by conjugation and/or click chemistry.

## Introduction

Positron emission tomography (PET) has become a practical, high-throughput clinical imaging modality for the visualization of biological processes in living systems. This technique, which utilizes positron-emitting radionuclides, is highly sensitive and requires the use of radiotracers that decay and produce two 511 keV γ-rays resulting from the annihilation of a positron and an electron. There are many factors to bear in mind when choosing the radioactive tracer for PET, two of them being the half-life and accessible production. In this regard, metal-based PET radioisotopes, such as ^68^Ga and ^64^Cu, have emerged as an excellent opportunity and alternative to traditional and short-lived PET radioisotopes (^11^C and ^18^F).^1^ With very useful physical properties, ^68^Ga [*t*_1/2_ = 67.71 min; β^+^ 89%; *E*(β^+^)_max_ = 1.9 MeV]^2^ can be easily produced in a commercially available ^68^Ge/^68^Ga generator system^3,4^ without the need for an on-site cyclotron. Meanwhile, ^64^Cu with a reasonably long half-life of 12.7 h is ideal for developing radiopharmaceuticals requiring long circulation times to achieve optimal uptake, therefore allowing delayed imaging or the use of notoriously slowly localizing antibodies. Furthermore, the dual decay characteristics of this radiometal, with positron [β^+^ 18%; *E*(β^+^)_max_ 653 keV] and beta [β^–^ 37%; *E*(β^–^)_max_ = 578 keV] emission,^5^ make it an attractive radioisotope for the development of dual PET imaging/therapy (theranostic) agents.^6^

A requirement in the development of radiopharmaceuticals based on radiometals is the presence of a bifunctional chelator (BFC) capable of binding to the metal at one terminus and containing a functional group for the linkage to a targeting vector at the second terminus. The targets are specified by a variety of biovectors that can be conjugated (attached) to the BFC agent. An optimal BFC must fulfill some requirements: (i) radiolabeling of the BFC should be efficient and rapid at low temperatures and low concentration at a pH suitable for biological applications; (ii) it should form thermodynamically stable and kinetically inert complexes with the metal to prevent any transmetalation *in vivo*; (iii) it must provide versatile conjugation chemistry; (iv) its preparation should be straightforward, quick, and cost-effective as well as scalable with as few reactions steps as possible. For the last years, significant effort has been made to find optimal BFCs for gallium and copper radionuclides, and many different chemical scaffolds, both cyclic and acyclic, have been promulgated. In general, macrocyclic chelators are kinetically more inert than acyclic chelators but may suffer from slower coordination. This is the case of the most widely used (and only clinically approved) chelator 1,4,7,10-tetraazacyclododecane-1,4,7,10-tetraacetic acid (DOTA), which requires heating above 80 °C and longer reaction times (30–90 min).^7^ Acyclic candidates, when properly designed, are often able to quantitatively coordinate radiometals in ca. 10 min at room temperature (RT). Fast RT labeling is important because of the half-lives of the radiotracers and becomes a crucial point when working with heat-sensitive molecules such as antibodies and their derivatives. Finding a BFC that responds optimally to the above requirements that is endowed with conjugative versatility remains a real challenge in this field.

**Chart 1:**
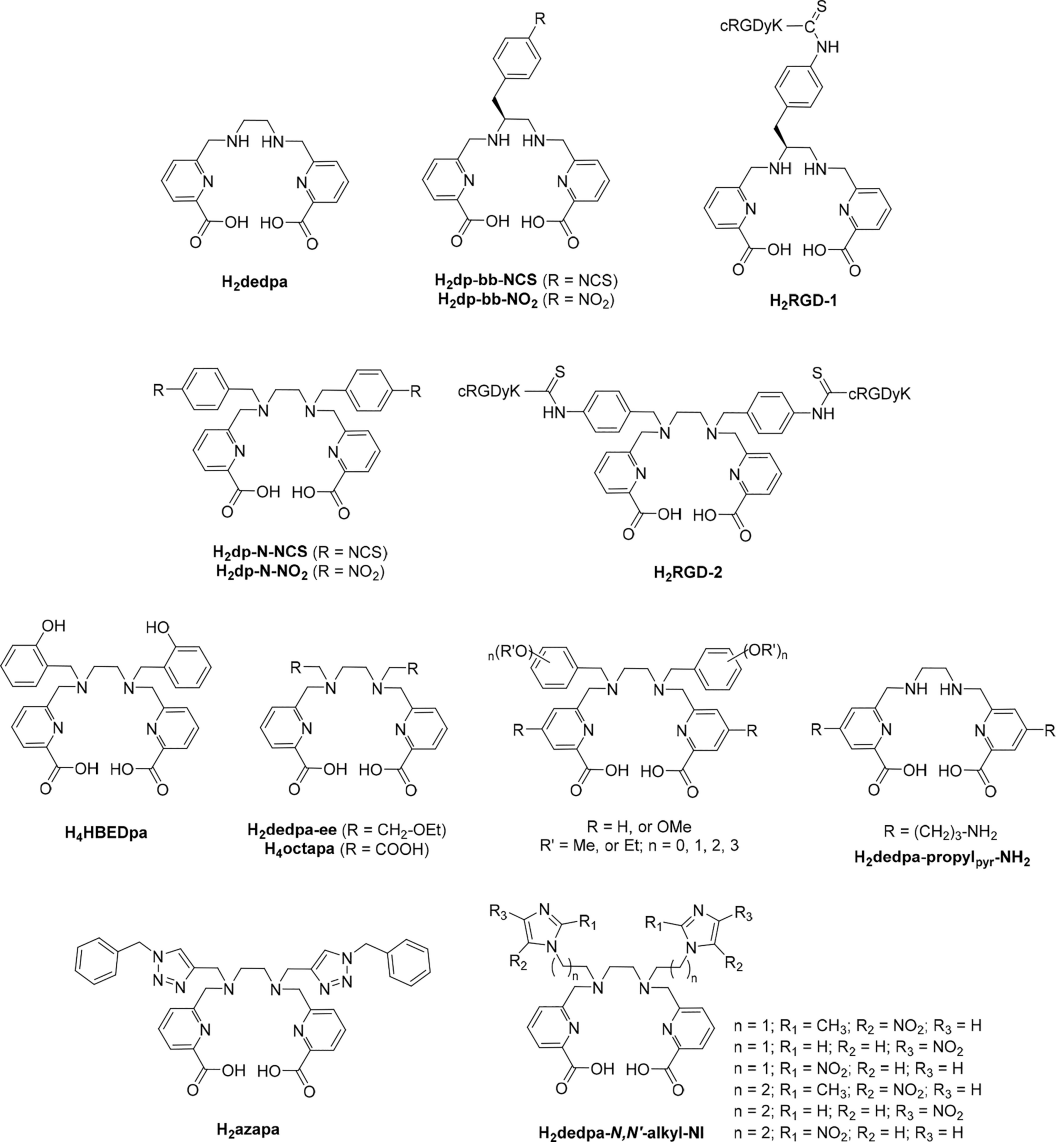
Structures of “dedpa” Family Members Previously Studied for [^64^Cu]Cu^2+^ and/or [^68^Ga]Ga^3+^ Labeling

Some years ago, looking into alternatives to macrocyclic BFCs, we found that the acyclic picolinic acid–base scaffold 1,2-bis[[(6-carboxypyridin-2-yl)methyl]amino]ethane (H_2_dedpa), first named H_2_bcpe and reported for Zn(II), Cd(II), and Pb(II) complexation,^8^ has properties of merit for [^68^Ga]Ga^3+^ PET imaging agent elaboration far superior to those of DOTA^9,10^ and rivalling those of another gold standard, 1,4,7-triazacyclononane-1,4,7-triacetic acid (NOTA):^1,11,12^ quantitative radiolabeling in only 10 min at RT at very low ligand concentrations, forming a metal complex of high thermodynamic stability and kinetic inertness.^13^ Starting from the H_2_dedpa platform, a diversity of functionalized derivatives have been constructed and their different capabilities for the development of Ga-based PET agents have been investigated, including hypoxia, heart uptake, and tumor uptake (Chart 1).^14−19^ Recently, H_2_dedpa has been functionalized with pyridylbenzofuran to design gallium-based islet amyloid imaging probes.^20^

**Chart 2:**
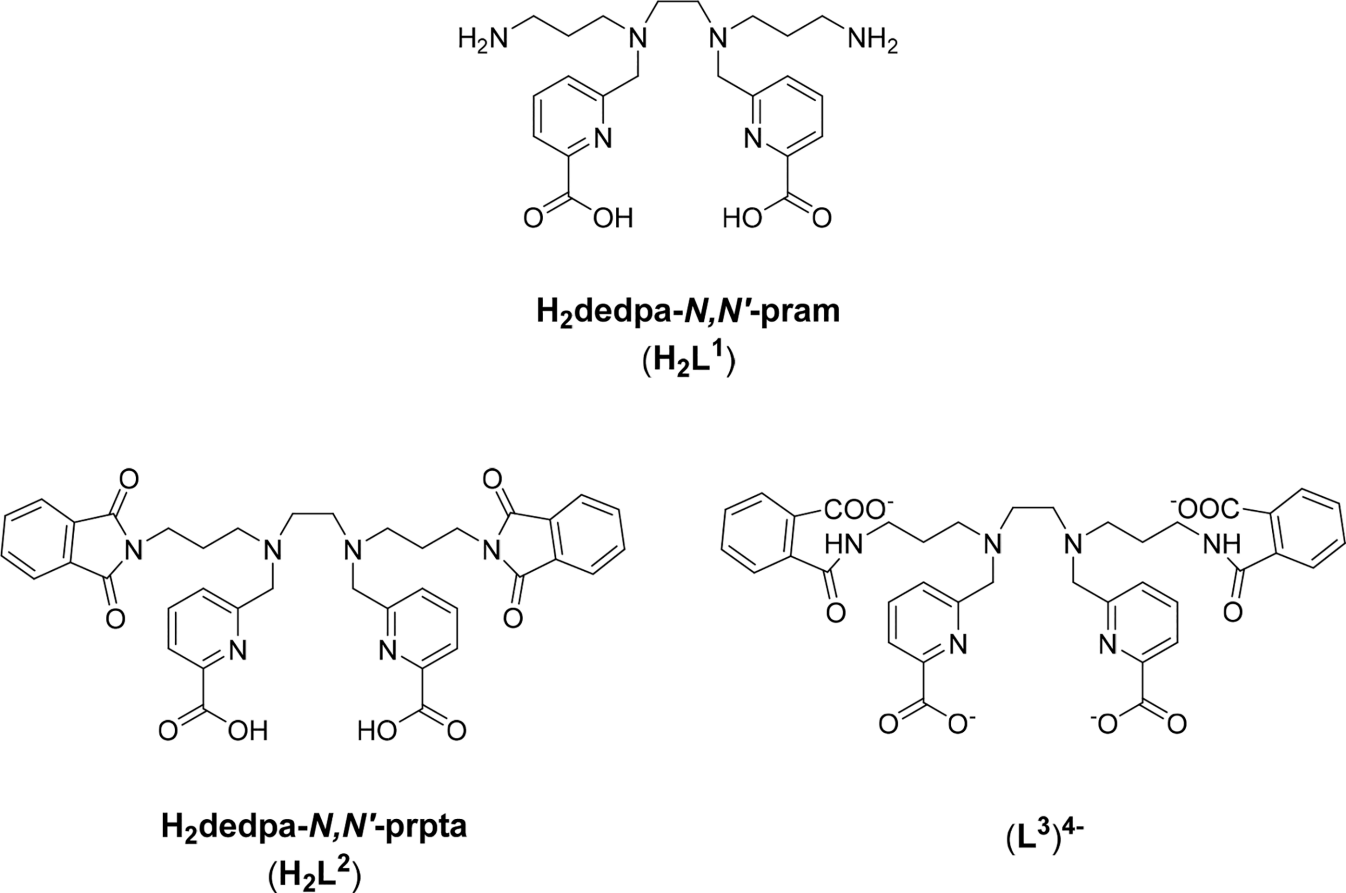
Chelators Investigated in This Work

The field of PET probe development is currently interested in finding BFCs that show great versatility in terms of both their coordination capacity and ease of conjugation. In particular, systems are sought that are easy and fast to prepare, are easily labeled with different radionuclides (e.g., ^68^Ga and ^64^Cu), and incorporate functional groups to allow wide diversity in conjugation. In this regard, the H_2_dedpa scaffold and functionalized and/or conjugated analogues have been probed for [^64^Cu]Cu^2+^ labeling with promising results,^21−23^ suggesting the versatility of this acyclic skeleton to coordinate metals of different nature and charge. Meanwhile, isothiocyanate and primary amines are suitable reactive groups for conjugation. Isothiocyanate groups were incorporated into the H_2_dedpa platform, giving rise to the chelators H_2_dp-bb-NCS and H_2_dp-N-NCS (Chart 1). Although those BFCs can be conjugated and the corresponding conjugated species exhibit acceptable properties for radiolabeling, the lengthy and challenging synthesis of H_2_dp-bb-NCS and H_2_dp-N-NCS resulted in extremely poor yields,^15^ limiting any real use. H_2_dedpa-propyl_pyr_-NH_2_, which incorporates a propylamine chain in the pyridyl groups (Chart 1), was also synthesized as a potential BFC with wide versatility. Unfortunately, radiolabeling studies with the longer half-lived [^67^Ga]Ga^3+^ confirmed that the complex [^67^Ga][Ga(dedpa-propyl_pyr_-NH_2_)]^+^ exhibited reduced stability compared to [^67^Ga][Ga(dedpa)]^+^, and, in addition, the conjugated analogue failed in [^67^Ga]Ga^3+^ labeling.^17^ As a continuation of this effort, herein we report the very promising derivative H_2_dedpa-*N*,*N*′-pram (H_2_L^1^; Chart 2) which incorporates propylamine chains attached to the two secondary amines of H_2_dedpa. The ability of this new member of the dedpa family to label both [^68^Ga]Ga^3+^ and [^64^Cu]Cu^2+^ has been investigated. The study is extended to chelators H_2_dedpa-*N*,*N*′-prpta (H_2_L^2^) and (L^3^)^4–^ (Chart 2) as simple structural models of the conjugated bifunctional agents that could be derived from H_2_L^1^.

## Results and Discussion

### Synthesis and Characterization

Chelator H_2_L^1^ has been thoughtfully devised on the basis of previous results reported with dedpa derivatives. From the comparative study of the dedpa-based BFCs reported to date, it is clear that any modification of the native dedpa skeleton must be carried out with great care because it can have a decisive effect on the coordination capability of the system: negatively affecting the thermodynamic stability and kinetic inertness of the complexes and, consequently, losing its usefulness for radiopharmaceutical applications.

The novel dedpa member H_2_L^1^, which incorporates reactive primary amine groups, has not been conceived as a BFC itself but as a readily accessible versatile intermediate, which in its ester form allows conjugated BFCs to be easily obtained and may even be used for click chemistry.

**Scheme 1:**
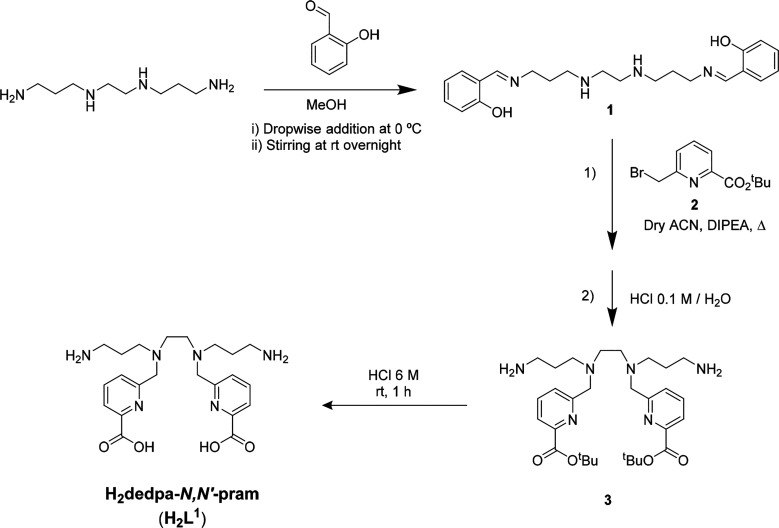
Synthesis of H_2_L^1^ Yields: 50% (**1**), 40% (**2**), 90% (H_2_L^1^).

As shown in Scheme 1, the *tert*-butyl ester of H_2_L^1^, *N*,*N*′-bis(3-aminopropyl)-*N*,*N*′-bis[6-(*tert*-butoxycarboxy)pyridin-2-yl]-1,2-diaminoethane (**3**) can be easily prepared via S_N_2 reaction, leading to alkylation of the secondary amines of 1,5,8,12-tetraazadodecane, a very affordable commercially available starting material. Selective protection of both primary amine groups of the tetramine is required, but neither benzoxycarbonyl (Cbz) nor *tert*-butyloxycarbonyl (Boc), commonly used as amine protecting groups, had adequate selectivity. In contrast, the less-often-utilized bis-imine selective functionalization strategy, suitably adapted, was successful. On the basis of this approach, salicylaldehyde was selected for imine formation.^24^ N-alkylation of the Schiff base (**1**) with *tert*-butyl 6-(bromomethyl)picolinate (**2**) in dry acetonitrile under argon, followed by deprotection of imine protecting groups using 0.1 M HCl and purification by reversed-phase column chromatography, leads to the expected compound **3** as a pure yellowish oil. These mild imine deprotection conditions allow the *tert*-butyl esters of the picolinate groups to remain intact, as confirmed by NMR spectroscopy and high-resolution mass spectrometry (HR-MS; Figures S1–S5 and S37). Acidic deprotection of the carboxylic groups with 6 M HCl affords H_2_L^1^ as a dihydrochloride salt.

**Scheme 2:**
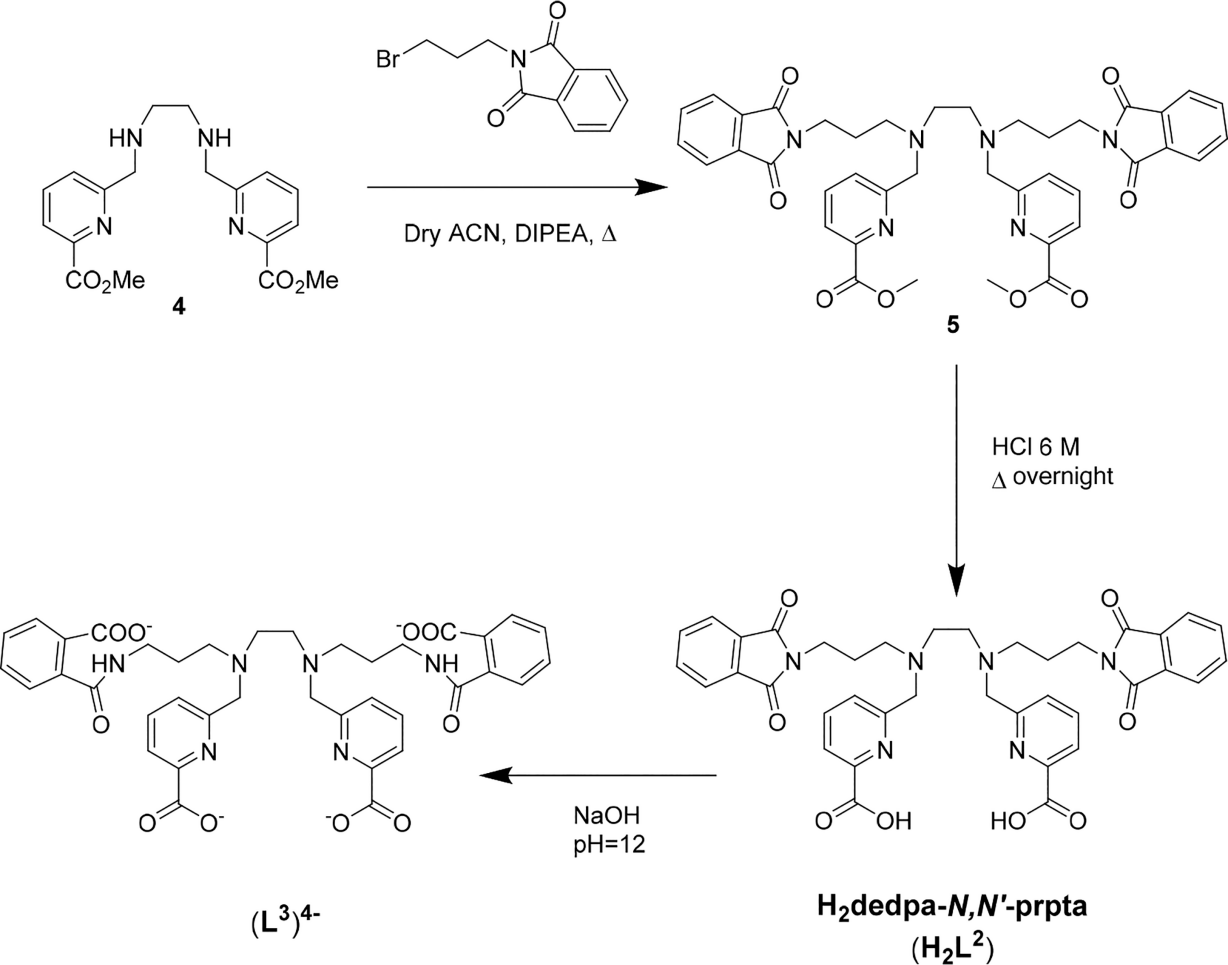
Synthesis of H_2_L^2^ Yields: 70% (**5**), 50% (H_2_L^2^).

**Figure 1:**
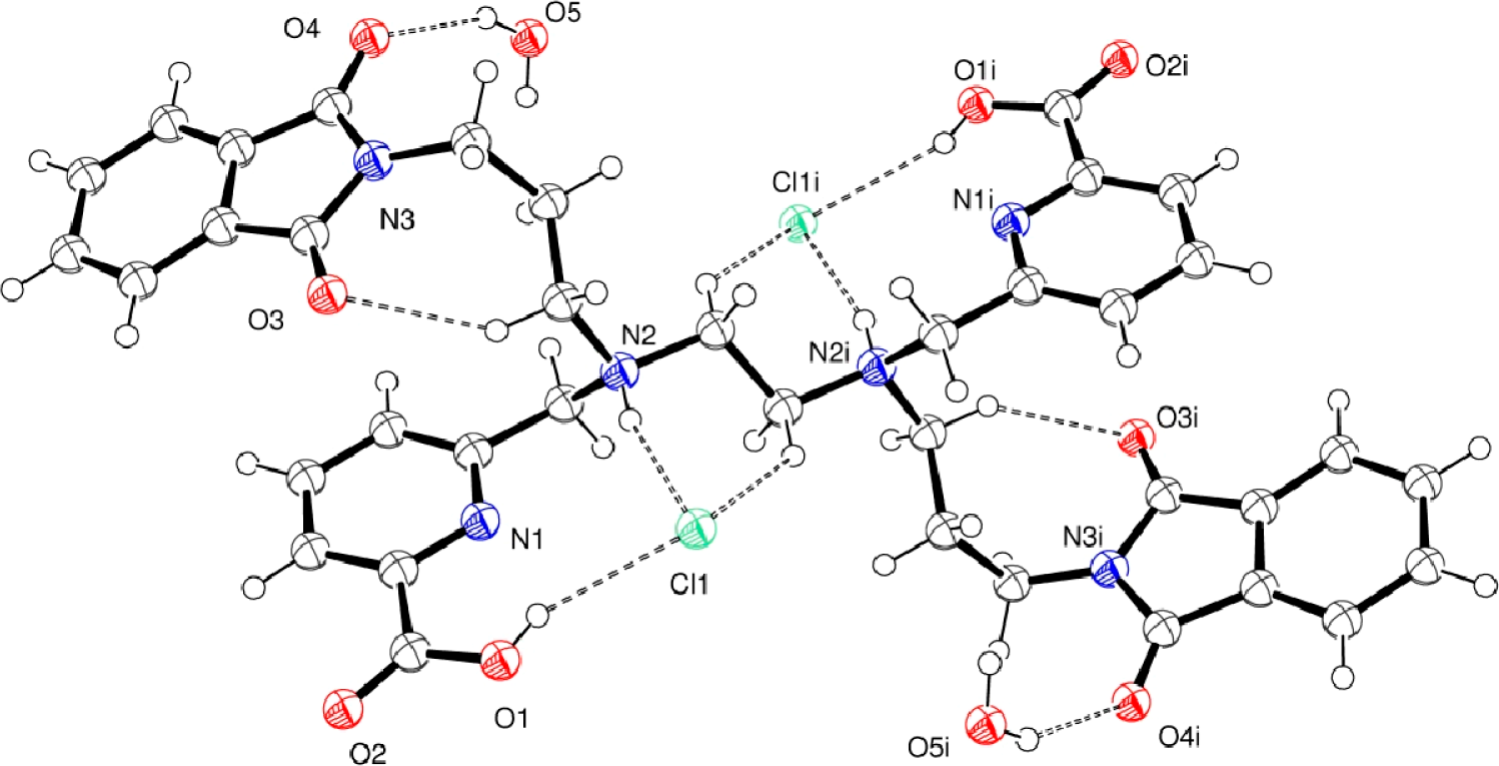
ORTEP diagram of [H_4_L^2^]Cl_2_·2H_2_O with atom labeling. Ellipsoids are shown at the 50% probability level. Hydrogen-bonding interactions: N2–H2 0.84(4) Å, H2···Cl1 2.31(4) Å, N2···Cl1 3.125(3) Å, N2–H2–Cl1 164(3)°; O1–H1 0.82(4) Å, H1···Cl1 2.44(4) Å, O1···Cl1 3.153(3) Å, O1–H1–Cl1 145(4)°; O5–H5A 0.85 Å, H5A···O4 2.11 Å, O5···O4 2.891(3) Å, O5–H5A–O4 152.2°; O5–H5B 0.85 Å, H5B···Cl1 2.41 Å, O5···Cl1 3.150(3) Å, O5–H5B–Cl1 145.2°.

The analogous H_2_L^2^, containing *N*-propylphthalimide pendant arms instead of 3-propylamine, was conceived as a very simple model of H_2_L^1^ wherein the amine groups are functionalized. Moreover, it is well-known that *N*-alkylphthalimides can undergo basic hydrolysis, which can be advantageous in the construction of a new system that incorporates pendant amide groups (L^3^)^4–^, being an even closer model to conjugates of the diamine derivative. Alkylation of the methyl ester-protected Me_2_dedpa (**4**), prepared as previously reported by us,^8^ with (bromopropyl)phthalimide yielded the methyl ester intermediate *N*,*N*′-bis(propylphthalimide)-*N*,*N*′-bis[6-(*tert*-butoxycarboxy)pyridin-2-yl]-1,2-diaminoethane (**5**), which was deprotected with 6 M HCl to give the expected chelator H_2_L^2^ again as its dihydrochloride salt (Scheme 2). The structure of this ligand salt was determined by X-ray diffraction analysis. The ligand crystallizes in the centrosymmetric *P*1̅ triclinic space group, and the asymmetric unit comprises a half-molecule. The crystals contain one [H_4_L^2^]^2+^ cation, two chloride anions, and two lattice water molecules (Figure 1). Ligand protonation occurs on the two tertiary amines of the ethylene backbone, which are arranged as far away from each other as possible to keep electrostatic repulsion to a minimum. In addition, both the two *N*-propylphthalimide pendant arms and the two picolinic acid residues are arranged trans (opposite) to one another, respectively, probably to avoid steric crowding of the aromatic rings. This arrangement is quite similar to that found for the previously reported H_4_HBEDpa.^19^ The conformation of the ligand also appears to be conditioned by the intermolecular hydrogen-bonding interactions between the protonated amine and the carboxylic acid groups and chloride anions (Figure 1), which is also observed in the structure of the parent [H_4_dedpa]^2+^, although no intramolecular hydrogen-bonding interactions exist.^8^ Lattice water molecules are involved in hydrogen-bonding interactions with phthalimide groups as well as chloride anions. Apart from that, two main parallel interactions are found: one between the oxygen atom of the carboxylate group of the pyridinecarboxylate moiety and the centroid of the benzene ring of the phthalimide close to it and one between an oxygen atom of the phthalimide moiety and the pyridinecarboxylate ring. The distances range from 3.2 to 3.7 Å, which is consistent with the existence of π stacking.

**Figure 2:**
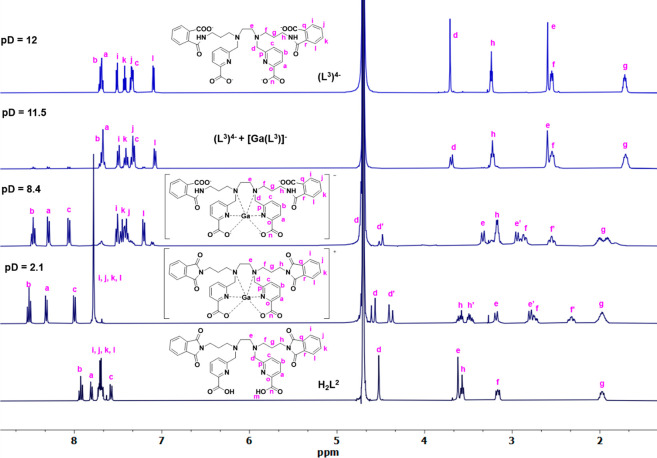
^1^H NMR spectra (400 MHz, 25 °C, D_2_O) of the complexation of Ga(III)-H_2_L^2^ varying pD. The spectra of the free ligands H_2_L^2^ (bottom) and (L^3^)^4–^ (top) are shown for reference. Note that the [GaL^2^]^+^ complex is fully formed at pD = 2.1, and at pD = 8.4, while (L^2^)^2–^ hydrolyses to form (L^3^)^4–^, the coordination of Ga(III) ion is maintained as the [GaL^3^]^−^ species. At pD = 11.5, most of (L^3^)^4–^ is unbound to the metal.

The basic hydrolysis of the phthalimide groups of H_2_L^2^ can be followed by NMR spectroscopy. At pD = 12 the only species present is the derivative (L^3^)^4–^, which incorporates amide functional groups on the pendant arms (lowest and uppermost spectra in Figures 2 and S21–S25). This is also corroborated by HR-ESI^–^-MS, where the expected peak at *m*/*z* 805.2183 corresponding to C_38_H_36_N_6_Na_3_O_10_ ([L^3^ + 3Na]^−^) is found at pH = 12; no peaks due to (L^2^)^2–^ or any intermediate hydrolysis species are observed. The peak due to [L^3^ + 5Na]^+^ is also found in ESI^+^-MS (Figure S41).

**Figure 3:**
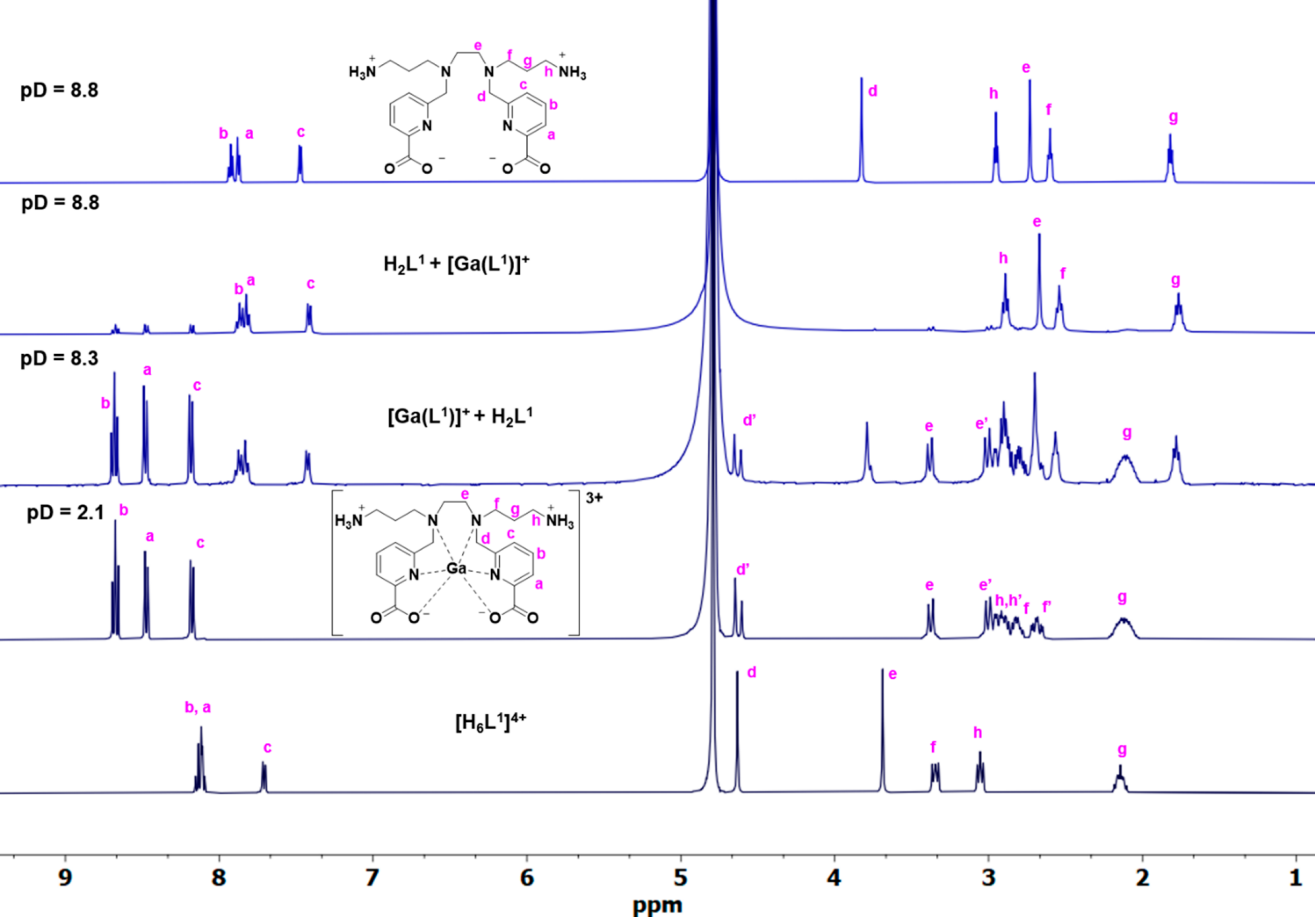
^1^H NMR (400 MHz, 25 °C, D_2_O) spectra of the complexation of Ga(III) with H_2_L^1^ at different pD values. The spectra of the free ligand H_2_L^1^ (bottom and top) are shown for reference.

Complexation of both chelators H_2_L^1^ and H_2_L^2^ with a “cold” (nonradioactive) Ga(III) ion was followed by NMR spectroscopy. The ^1^H and ^13^C NMR spectra were recorded from a D_2_O solution at 298 K and assigned on the basis of two-dimensional (2D) COSY, HSQC, and HMBC experiments (Figures S26–S36). For both H_2_L^1^ and H_2_L^2^, the study of “cold” Ga(III) complexation was carried out at different pD values, and the corresponding ^1^H NMR spectra are shown in Figures 3 and 2, respectively. ^1^H and ^13^C NMR spectra of the free chelator H_2_L^1^ confirm *C*_2*v*_ symmetry, with only half of the resonances expected. NMR spectroscopy confirms that the gallium complex [Ga(H_2_L^1^)]^3+^ is fully formed at pD = 2.1. *C*_2_ symmetry is retained in the complex, as only half the resonances corresponding to the carbon nuclei of the ligand backbone are present (Figure S30). Meanwhile, in the ^1^H NMR spectrum, it can be seen that coordination of the ligand causes not only a downfield shift of the signals but also a diastereotopic splitting of the methylene hydrogen atoms on the picolinate ring, hydrogen atoms in the ethylenediamine bridge, as previously observed,^16^ and hydrogen atoms of the 3-aminepropylene pendant. Although specific assignment of the axial and equatorial CH_2_ protons is impossible on the basis of the 2D NMR spectra, they can be carried out using the stereochemically dependent proton shift effect, resulting from polarization of the C–H bonds by the electric field effect caused by the cation charge. This results in a deshielding effect of the equatorial protons, which are pointing away from the metal ion^8,25,26^ (Figure 3, apostrophe denotes axial protons). The ^1^H NMR aromatic patterns of the [Ga(H_2_L^1^)]^3+^ and [GaL^1^]^+^ species [a triplet at 8.68 ppm (Hb) and two doublets at 8.48 ppm (Ha) and 8.18 ppm (Hc)], confirm the downfield shift with respect to the free ligand due to metal complexation, as well as the presence of only one symmetric complex species (Figures 3 and S26).

NMR spectroscopy also confirms that the Ga(III) complex with H_2_L^2^ is formed under acidic conditions, and at pD = 2.1, the ^1^H NMR spectrum is as expected for the [GaL^2^]^+^ complex, with the corresponding diastereotopic splitting for the methylene, ethylenediamine bridge, and propylene linker hydrogen atoms (Figure 2, apostrophe denotes axial protons). Hydrolysis of the phthalimide groups of (L^2^)^2–^ is found at pD = 8.4, and under these conditions, the only species present in solution is [GaL^3^]^−^, where the chelating ligand (L^3^)^4–^ contains amide groups instead of phthalimide ones and Ga(III) ions remain tightly coordinated to the N_4_O_2_ core of the dedpa^2–^ scaffold. Strong basic conditions are necessary to release the metal ion. Although at pD = 11.5 most of (L^3^)^4–^ is not bound to the metal, it is still possible to see signals from the [GaL^3^]^−^ complex. These results point out that the chelator containing amide groups is also able to effectively complex Ga(III), as do (L^1^)^2–^ [dedpa-*N*,*N*′-pram] and (L^2^)^2–^ [dedpa-*N*,*N*′-prpta].

### X-ray Crystal Structures of Metal Complexes

**Figure 4:**
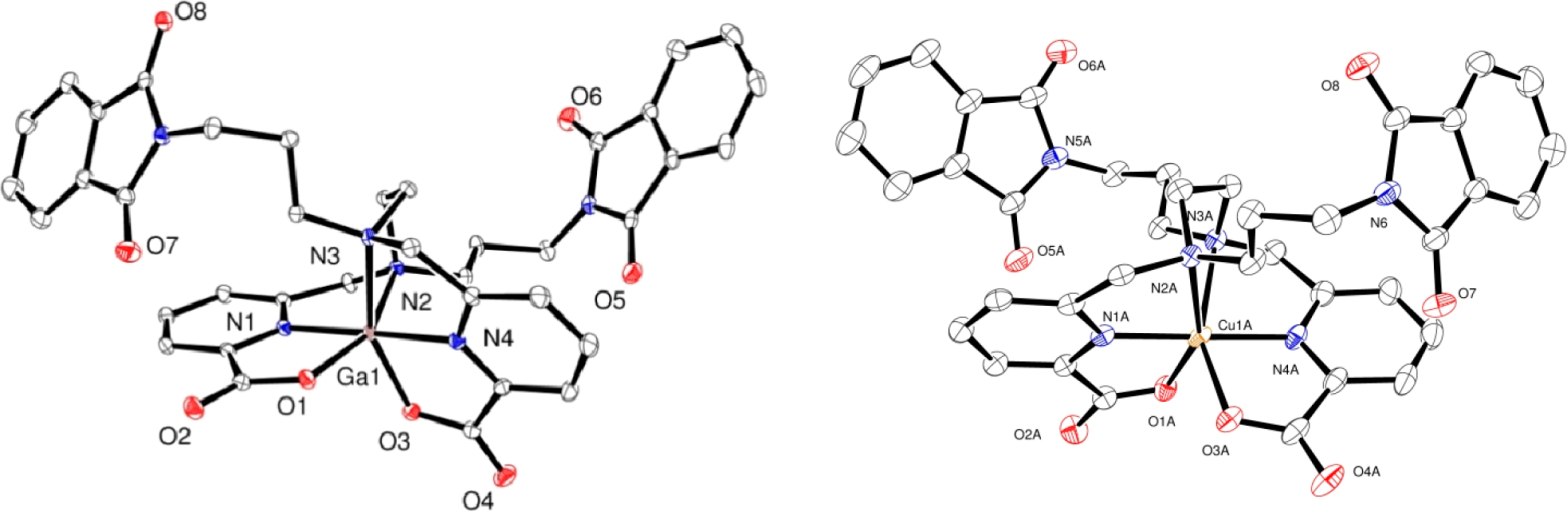
Solid-state X-ray structure of the cation in [GaL^2^]NO_3_ (left) and [CuL^2^] (right). Ellipsoids are drawn at 50% probability. Only heteroatoms are labeled and hydrogen atoms are omitted for clarity.

**Figure 5:**
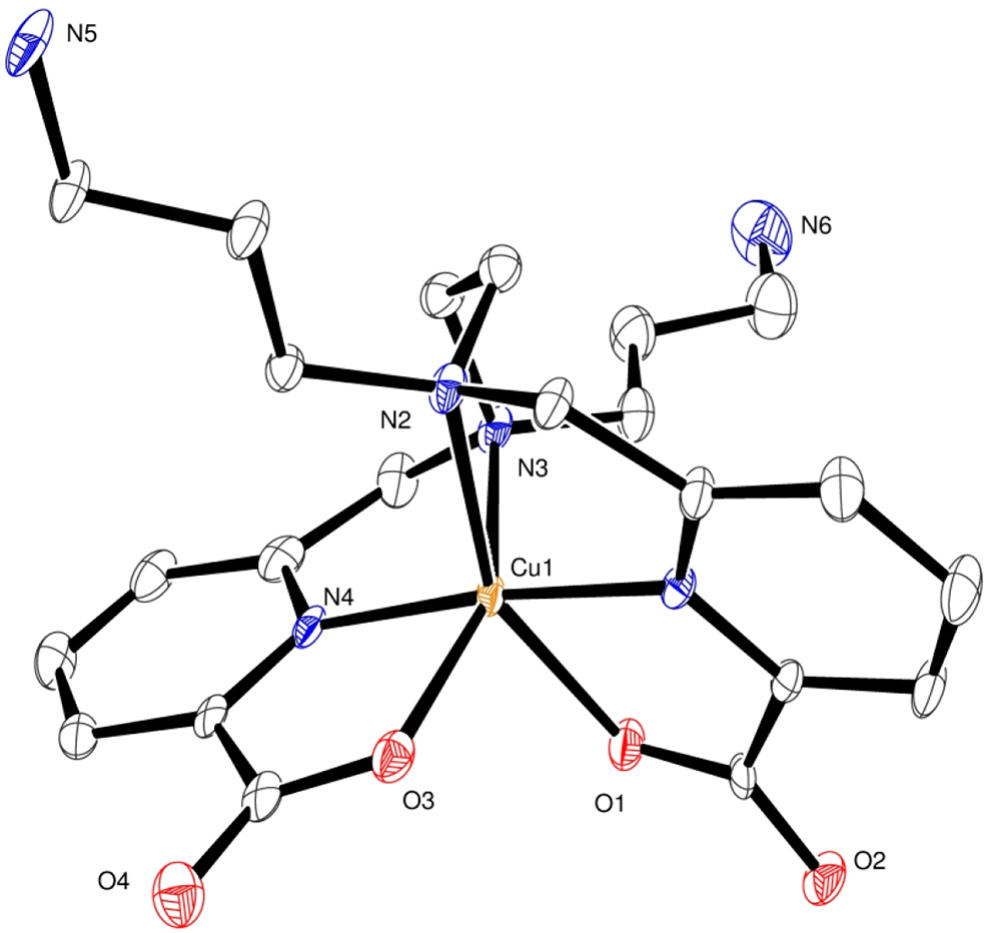
Solid-state X-ray structure of the cation in [Cu(H_2_L^1^)]Cl_2_. Ellipsoids are drawn at 50% probability. Only heteroatoms are labeled and hydrogen atoms are omitted for clarity.

Figures 4 and 5 display the solid-state structures of [Ga(dedpa-*N*,*N*′-prpta)]^+^, [Cu(dedpa-*N*,*N*′-prpta)], and [Cu(H_2_dedpa-*N*,*N*′-pram)]^2+^, denoted as [GaL^2^]^+^, [CuL^2^], and [Cu(H_2_L^1^)]^2+^, respectively, obtained by X-ray crystallographic analysis. It is noteworthy that both amino groups of (L^1^)^2–^ are protonated in the latter structure. This is not surprising, given the strongly basic character of the primary amines present in the pendant chains. The typical N_4_O_2_ core found for crystal structures reported thus far for Ga(III) and Cu(II) complexes of dedpa^2–^ and its bifunctional derivatives is also found in these three new complexes, corroborating the notion that each picolinate moiety of these ligands provides a versatile coordination pocket for a variety of metal ions. In the three structures, the functionalized propyl pendants are pointed in opposite directions, away from the metal-binding sphere, which is beneficial for the radiopharmaceutical application—often functionalization alters coordination of the metal ion.

It is well-known that, although there is not always parallelism between the structures of species in solution and in the solid state due to crystal packing effects and *in vitro* stability tests are therefore better predictors of kinetic inertness, data on bond distances and angles obtained from X-ray diffraction structures provide valuable complementary information. The previous studies point to the high degree of symmetry for metal-dedpa^2–^ complexes, which, along with an approximately equally distributed set of metal–ligand bond lengths, is thought to correlate well with their high stability and favorable biological properties.^13^Table 1 contains the coordination sphere bond lengths of the new [Ga(dedpa-*N*,*N*′-prpta)]^+^ complex compared to those of the parent [Ga(dedpa)]^+^ complex and other family members, with the Ga–L bond lengths found in our new complex being similar to those of previously reported systems. Selected angles of the coordination sphere are given in Table S1. The Ga–N_pyr_ and Ga–O_coo_ bonds have the same values as those found in the “unfunctionalized” [Ga(dedpa)]^+^, whereas the Ga–N_en_ bond lengths are somewhat longer, in line with other systems containing these nitrogen atoms also functionalized. This lengthening of the Ga–N_en_ bond distances observed in [GaL^2^]^+^ translates into an opening of the N_pyr_–Ga–N_pyr_ angle, which is increased by about 7° with respect to that observed in the parent derivative [178.03(4)° in [Ga(L^2^)]^+^ vs 170.97(6)° in [Ga(dedpa)]^+^]. This, however, hardly affects to the N_en_–Ga–O_COO_ angles, which have very similar values [155.41(3)° and 156.40(3)°].

**Table 1:** Bond Lengths of the Coordination Spheres of [GaL^2^]^+^ Compared to [Ga(dedpa)]^+^,^13^ [Ga(dedpa-*N*,*N*′-alkyl-NI)],^16^ [Ga(H_2_BEDpa)]^+^,^19^ and [Ga(dp-*N*-NO_2_)]^+^ ^13^

	[GaL^2^]^+^	[Ga(dedpa)]^+^	[Ga(dedpa-*N*,*N*′-propyl-4NI)]^+^	[Ga(dedpa-*N*,*N*′-ethyl-2NI)]^+^	[Ga(H_2_BEDpa)]^+^	[Ga(dp-*N*-NO_2_)]^+^
Ga–N1 (pyr-N)	1.9877(9)	1.9866(16)	1.985(9)	1.980(2)	1.975(15)	1.992(5)
Ga–N2 (en-N)	2.1980(9)	2.1115(16)	2.177(3)	2.2172(19)	2.147(15)	2.188(5)
Ga–N3 (en-N)	2.1600(9)	2.1132(16)	2.183(2)	2.2172(19)	2.185(15)	2.159(5)
Ga–N4 (pyr-N)	1.9932(9)	1.9902(16)	1.996(9)	1.980(2)	1.982(15)	1.981(5)
Ga–O1 (COO^–^)	1.9701(8)	1.9708(13)	2.038(12)	1.9626(16)	1.982(12)	1.967(4)
Ga–O3 (COO^–^)	1.9805(8)	1.9828(13)	2.068(10)	1.9626(16)	1.969(13)	1.976(4)

The solid-state molecular structures of [CuL^2^] and [Cu(H_2_L^1^)]^2+^ feature a distorted octahedral metal–ligand environment, typical of six-coordinate Cu(II) complexes. The bond lengths of their coordination spheres, compared to those of [Cu(dedpa)] and other functionalized derivatives, are given in Table 2. In contrast to the Ga(III) complex, in the Cu(II) complexes, both M–O_COO_ distances are quite different from each other, with the 0.26 Å difference found in the previously reported parent compound [Cu(dedpa)] being especially striking.^21^ In our derivative [Cu(H_2_L^1^)]^2+^, both bond distances differ much less (by about 0.1 Å), a value similar to that found in [Cu(dedpa-*N*,*N*′-propyl-2NI)], whereas in [CuL^2^], the difference between these two bond distances is even considerably smaller (only 0.051 Å). Even though these two distances do not differ much from each other, this Cu(II) complex of (L^2^)^2–^ also shows a Jahn–Teller distortion, as is often found in hexacoordinated Cu(II) complexes. This is confirmed by the particularly long value of the Cu–N(2)_en_ bond distance (2.355 Å). Jahn–Teller distortion is also observed in [Cu(H_2_L^1^)]^2+^, although in this species, this effect is quite small.

**Table 2:** Bond Lengths of the Coordination Spheres of [CuL^2^] and [Cu(H_2_L^1^)]^2+^, Compared to [Cu(dedpa)],^21^ [Cu(azapa)],^22^ and [Cu(dedpa-*N*,*N*′-propyl-2-NI)]^23^

	[CuL^2^]	[Cu(H_2_L^1^)]^2+^	[Cu(dedpa)]	[Cu(azapa)]	[Cu(dedpa-*N*,*N*′-propyl-2NI)]
Cu–N1 (pyr-N)	1.941(4)	1.952(4)	2.0008(12)	1.935(1)	1.9664(13)
Cu–N2 (en-N)	2.355(3)	2.293(4)	2.3171(13)	2.326(1)	2.3173(14)
Cu–N3 (en-N)	2.274(5)	2.232(4)	2.1364(13)	2.326(1)	2.2562(13)
Cu–N4 (pyr-N)	1.951(2)	1.934(4)	1.9386(13)	1.935(1)	1.9263(13)
Cu–O1 (COO^–^)	2.139(3)	2.203(3)	2.3014(11)	2.120(1)	2.2128(12)
Cu–O3 (COO^–^)	2.088(3)	2.104(4)	2.0430(10)	2.120(1)	2.0741(11)

### Solution Thermodynamics

H_2_L^1^ and H_2_L^2^ were then studied in order to evaluate the thermodynamic driving force (log *K*_M_*p*_H_*q*_L_*r*__) of the reaction between the ligand (L) and metal ion (M) (M = Ga(III) or Cu(II)). This reaction is conventionally expressed through the sum of equilibrium reactions expressed as *p*M + *q*H^+^ + *r*L ↔ M_*p*_H_*q*_L_*r*_. It should be noted that also protons in ligands (H^+^) play a role in this equilibrium because they compete with the metal ion in complex formation to occupy coordinating electron pairs of ligands. Therefore, protonation constants of H_2_L^1^ and H_2_L^2^ were determined through a variety of techniques: ^1^H NMR titrations, in-batch acidic UV titrations, and combined potentiometric–spectrophotometric titrations (Table 3).

**Table 3:** Protonation Constants^a^ (log *K*_H_*q*_L_) of H_2_L^1^ and H_2_L^2^

equilibrium reaction	H_2_L^1^	H_2_L^2^
L + H^+^ ⇆ HL	10.86(1)	8.14(1)
HL + H^+^ ⇆ H_2_L	9.91(1)	4.98(1)
H_2_L + H^+^ ⇆ H_3_L	6.78(1)	3.22(1)
H_3_L + H^+^ ⇆ H_4_L	4.23(1)	2.14(4)
H_4_L + H^+^ ⇆ H_5_L	2.79(1)	
H_5_L + H^+^ ⇆ H_6_L	2.01(6)	
H_6_L + H^+^ ⇆ H_7_L	0.37(2)^b^	
∑log *K*_HqL_	36.95	18.48

a Values were obtained from UV potentiometric titrations (25 °C, *l* = 0.2 cm, and *I* = 0.16 M NaCl). *K*_H_*q*_L_ defined as [H_*q*_L]/([H][H_*q*–1_L]).

b From acidic in-batch UV titrations [25 °C, *l* = 1 cm, *I* = 0.16 M NaCl (when possible)]. Charges are omitted for clarity.

H_2_L^1^ has eight potential protonation sites. The most basic protonations are expected to be on the primary amines on the propylamine pendants. ^1^H NMR titrations (Figures S45 and S46) showed that methylenic protons adjacent to −NH_2_ (Hh and Hg) reasonably undergo a downfield shift with protonation (pD interval 12.3–9) as the electron density is being donated to the proton from the ligand. Therefore, protonation constants calculated through potentiometric titrations log *K*_1_ = 10.86(1) and log *K*_2_ = 9.91(1) are assigned to the primary amines. Protonation of tertiary amines in the backbone [log *K*_3_ = 6.78(1) and log *K*_4_ = 4.23(1)] is evidenced by the expected downfield shift of methylenic protons adjacent to the picolinate donor groups (Hd) as well as methylenic protons He and Hf at the 8–3 pD interval. Carboxylate donors in the picolinic pendants protonate with log *K*_5_ = 2.79(1) and log *K*_6_ = 2.01(1). The final protonation log *K*_7_ = 0.37(2) was calculated from in-batch acidic UV experiments and is attributed to a pyridine nitrogen atom (Figure S51b).

Similarly, protonation constants of H_2_L^2^ were determined through combined potentiometric–spectrophotometric titrations, following diagnostic spectral changes on the picolinate chromophore. Titrations of an acidified aqueous solution of H_2_L^2^ in the pH range of 1.8–9.5 allowed the calculation of protonation constants relative to tertiary amines [log *K*_1_ = 8.14(1) and log *K*_2_ = 4.98(1)] and picolinic carboxylate donors [log *K*_3_ = 3.22(1) and log *K*_4_ = 2.14(4)].

The spectrophotometric data showed that, at pH > 9 (Figures S47–S49), species (L^2^)^2–^ starts to hydrolyze as the phthalimide chromophore with λ_max_ at 300 nm disappeared to form a different ligand (L^3^)^4–^; therefore, those data were excluded from the calculations. This (L^3^)^4–^ species was successfully identified through NMR studies (*vide supra*).

Complex formation equilibria of H_2_L^1^ with Ga(III) and Cu(II) were studied by combined potentiometric–spectrophotometric titrations and in-batch acidic UV titrations, following spectral changes with the pH on the picolinate chromophore at λ = 270 nm [for Ga(III) complexes] and the colored absorption band at λ = 740 nm [for Cu(II) complexes]. For H_2_L^2^, only in-batch acidic UV experiments were possible because the ligand reacts to form (L^3^)^4–^ at basic pH, obviating any fitting of the potentiometric data. Nonetheless, the complexes formed from acidic pH are the ML complexes as opposed to those with H_2_L^1^, where, with either Ga(III) or Cu(II), the [Ga(H_2_L^1^)]^3+^ or [Cu(H_2_L^1^)]^2+^ complex is formed from acidic pH with the propyleneamine arms still protonated (Figures S50–S61). This is further supported by X-ray crystallography of the [GaL^2^]^+^, [CuL^2^], and [Cu(H_2_L^1^)]^2+^ complexes (Figures 4 and 5).

With both ligands, complexation with the Cu(II) ion starts at a lower pH than that with Ga(III). It is interesting to note that the [GaL^1^]^+^ complex has a higher stability constant compared to that of [CuL^1^] (0.75 log unit) because the propyleneamine pendants in [Ga(H_2_L^1^)]^3+^ deprotonate at a lower pH compared to those in [Cu(H_2_L^1^)]^2+^. Despite the fact that the primary amines do not participate in the coordination with either of the metal ions, the lower protonation found in the case of the [Ga(L)]^+^ species could be explained by the zwitterionic nature of the complex in which amine protonation and hydroxide coordination to Ga(III) results in a formally monoprotonated or nonprotonated complex (Tables 4 and S3). Indeed, the preference of Ga(III) ion for the OH^–^ ion at higher pH, as opposed to Cu(II) [where the complex remained intact at the end of the potentiometric titrations at pH ∼ 11 (Figures S56 and S61)], is shown by the observed free ligand from pH ∼ 8–9 during the potentiometric–spectrophotometric titrations (Figures S50 and S55), which aligns with the Ga(III) complexation, followed by ^1^H NMR (*vide supra*).

**Table 4:** Stability Constants (log *K*_ML_)^a^ and pM^b^ Values of H_2_L^1^, H_2_L^2^, H_2_dedpa, DOTA, and NOTA with M^2+^ = Cu^2+^ and M^3+^ = Ga^3+^ Metal Ions

	log *K*_ML_ (M = Cu^2+^)	pCu	log *K*_ML_ (M = Ga^3+^)	pGa
H_2_L^1^^c^	23.05(2)	21.96	23.80(2)	19.5
H_2_L^2^^c^	22.70(2)	22.8	20.69(1)	20.8
H_2_dedpa	19.16(5)^d^	18.5^d^	22.9(1)^e^	22.2^e^
DOTA	22.21(1)^f^	16.2	21.33^g^	18.5^g^
NOTA	21.6^d^	19.2	30.98^g^	27.9^g^

a *K*_ML_ defined as [ML]/([M][L]) (charges are omitted for clarity).

b pM defined as −log [M_free_] when [L] = 10 μM and [M] = 1 μM at pH = 7.4.

c This work, at 25 °C and *I* = 0.16 M (NaCl). p*K*_a_ values of other complex species with either H_2_L^1^ or H_2_L^2^ and Ga(III) and Cu(II) are presented in Table S3.

d From ref (21).

e Corrected value (see the Supporting Information).

f From ref (28).

g From ref (13).

In the course of the studies presented here with H_2_L^1^ and H_2_L^2^, it was concluded that the [Ga(dedpa)]^+^ stability constant [log *K*_ML_ = 28.11(8)] reported in 2010^13^ was overestimated. It was calculated through only ligand–ligand competition using ethylenediaminetetraacetic acid (EDTA) as the ligand competitor. This method of stability constant determination should have been accompanied by a supporting spectroscopic technique such as ^1^H NMR, UV–vis, or a simple potentiometric determination in competition with the [Ga(OH)_4_]^−^ ion at basic pH. There is a section in the Supporting Information addressing this issue and showing that the correct stability constant for [Ga(dedpa)]^+^ is log *K*_ML_ = 22.9(1) (see the potentiometric curve in Figure S64).

A better thermodynamic descriptor of the metal complex stability than log *K*_ML_ is the pM value. pM is defined as the metal-free concentration (−log [M_free_]) at standard conditions ([L] = 10 μM; [M] = 1 μM at pH = 7.4)^27^ and allows for a comparison of metal scavenging between different chelators with different basicities, denticities, protonation states, and metal complex stoichiometries. In fact, despite the higher [GaL^1^]^+^ stability constant [log *K*_ML_ = 23.8(2)] with respect to that of [Ga(dedpa)]^+^ [log *K*_ML_ = 22.9(1)] or [GaL^2^]^+^ [log *K*_ML_ = 20.61(1)], the pGa value for H_2_L^1^ (19.5) is smaller than that of H_2_dedpa or H_2_L^2^ because of the higher overall basicity of H_2_L^1^ (Table S4). However, as shown by the ^1^H NMR titrations for Ga(III), with the three new ligands, H_2_L^1^, H_2_L^2^, or (L^3^)^4–^ at physiological pH, the metal coordination is maintained through the dedpa^2–^ scaffold. Additionally, higher log *K*_ML_ and pCu values are found for both H_2_L^1^ and H_2_L^2^ with respect to those of H_2_dedpa, DOTA, or NOTA.

It is widely known that the thermodynamic stability does not necessarily correlate with kinetic inertness, particularly *in vivo*. Therefore, a complete study of the stability of any system of interest for *in vivo* application should include kinetic inertness studies. In this regard, an *in vitro* assessment of the kinetic inertness can be made through acid-assisted dissociation kinetic experiments. Solutions containing each of the ligands (H_2_L^1^ or H_2_L^2^) and Ga(III) ions in a 1:1 molar ratio were incubated in 5 M HCl. Under these conditions, both Ga(III) complexes displayed first-order dissociation kinetics with half-lives of 1.6 and 2.1 h, respectively (Figures S65 and S66). In contrast, Cu(II) complexes incubated in 2 M HCl for months did not show any decomplexation; however, it cannot be concluded that this is kinetically inert but is rather thermodynamically stable even in 2 M HCl (Figures S57A and S59A).

Given the high affinity of the new H_2_dedpa ligands toward both Cu(II) and Ga(III) metal ions in solution, radiolabeling studies were performed to assess possible radiopharmaceutical application, including also stability studies in human serum, which are even more relevant as an indicator of their *in vivo* kinetic inertness.

### ^68^Ga and ^64^Cu Radiolabeling

**Figure 6:**
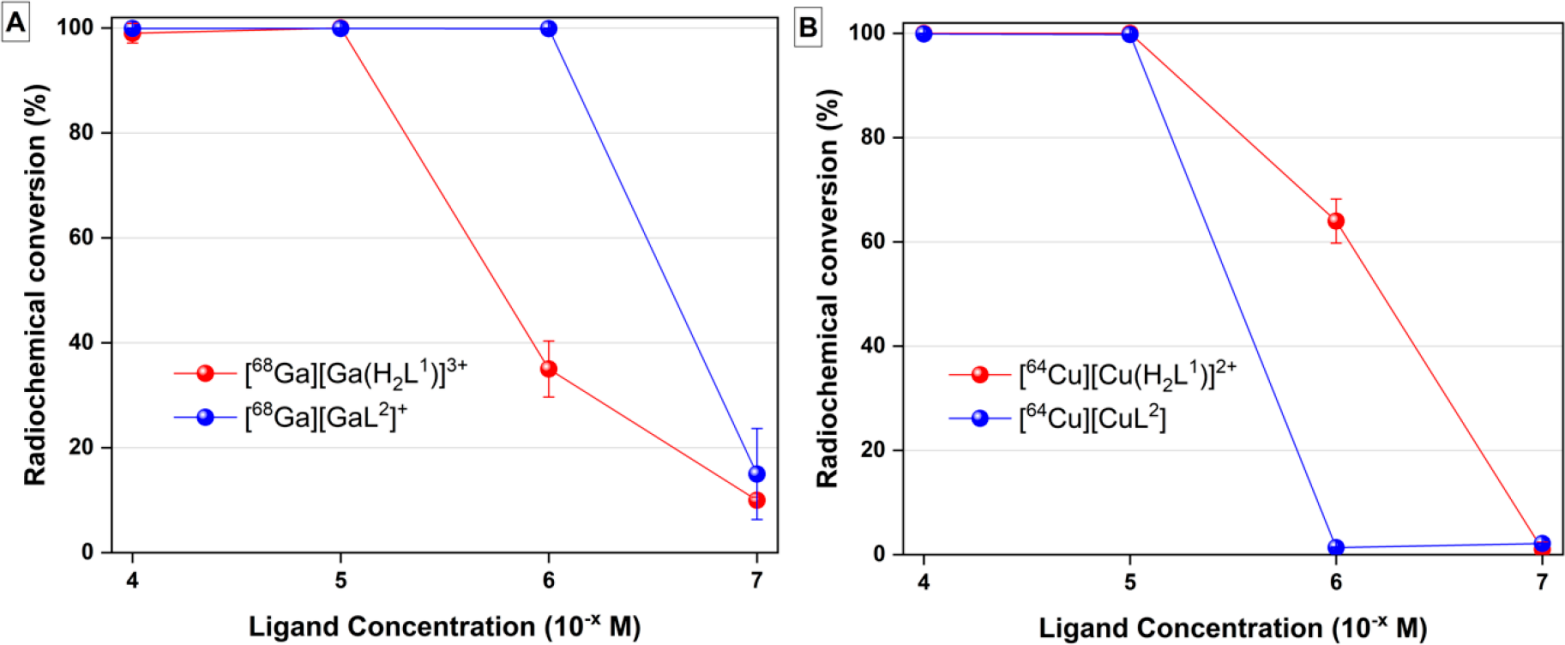
(A) RCC yields (%) of [^68^Ga][Ga(H_2_L^1^)]^3+^ and [^68^Ga][GaL^2^]^+^ in NaOAc buffer (2 M) at pH = 7.4 and ambient temperature. (B) RCC yields (%) of [^64^Cu][Cu(H_2_L^1^)]^2+^ and [^64^Cu][CuL^2^] in NaOAc buffer (0.5 M) at pH = 7 and ambient temperature.

Under mild reaction conditions (RT, pH = 7.4, NaOAc buffer), both H_2_L^1^- and H_2_L^2^-bound [^68^Ga]Ga^3+^ within 15 min of pH-dependent radiolabeling showed quantitative conversion at pH = 4, 6, and 7.4 (Table S5). The latter pH was chosen for the following concentration-dependent studies as the most representative for future *in vitro* and *in vivo* studies. [^68^Ga]Ga^3+^ quantitatively radiolabeled (>99%) H_2_L^1^ at 10^–5^ M reaction concentration, producing molar activities around 3.70 MBq/nmol as determined by radio-TLC (TLC = thin-layer chromatography; Figures 6 and S68). The radiolabeling efficiency of H_2_L^1^ dropped to 35% and 10% at 10^–6^ and 10^–7^ M reaction concentrations, respectively. H_2_L^2^ was quantitatively radiolabeled at 10^–6^ M reaction concentration, producing molar activities of 37.0 MBq/nmol, as determined by radio-TLC. The radiochemical conversion (RCC) of H_2_L^2^ dropped to 15% at 10^–7^ M, and thus no lower concentrations were tested.

The affinity of the model system, H_2_L^2^, for gallium is higher than that of H_2_L^1^ as corroborated by the higher pGa value in the solution thermodynamic studies performed with Ga(III) (*vide supra*); this was corroborated in the radiolabeling studies. The radiolabeling performance of H_2_L^1^ was analogous to that for the H_2_dedpa-*N*,*N*′-alkyl-NI chelators and for H_2_dedpa-propyl_pyr_-NH_2_ because they were also labeled by [^67/68^Ga]Ga^3+^ up to 10^–5^ M reaction concentration.^16,17^ Nonetheless, H_2_L^1^ presents the distinct advantage of a promising site for the functionalization for proof-of-principle studies in bimodal fluorescence and nuclear imaging, as opposed to H_2_dedpa-propyl_pyr_-NH_2_, whose picolinate pendants were negatively impacted once conjugated to the fluorophore.^17^ Other H_2_dedpa-based BFCs, either similarly exploiting the aliphatic secondary amines, such as H_2_RDG-2 and H_2_dp-N-NO_2_, or adding chirality to the ethylenediamine backbone (H_2_dp-bb-NO_2_ and H_2_RDG-1) for functionalization, quantitatively incorporated [^68^Ga]Ga^3+^ up to 10^–6^ M reaction concentration,^15^ showing the same affinity for gallium as the model system H_2_L^2^ did. The advantages of both H_2_L^1^ and H_2_L^2^ compared to bioconjugation on the ethylenediamine backbone, as in H_2_dp-bb-NO_2_ or H_2_dp-bb-NCS, are their ease of synthesis and the possibility of carrying two biotargeting molecules instead of one.

The radiolabeling of H_2_L^1^ and H_2_L^2^ with [^64^Cu]Cu^2+^ was also assessed under mild conditions (15 min, RT, pH = 7, NaOAc buffer). Quantitative labeling of both H_2_L^1^ and H_2_L^2^ with [^64^Cu]Cu^2+^ was observed at 10^–5^ M (Figure 6), achieving molar activities of 3.30 MBq/nmol, as determined by radio-TLC. The H_2_L^1^ and H_2_L^2^ radiolabeling studies demonstrate not only that functionalization of the secondary amines of the backbone does not negatively impact the fast complexation kinetics of the H_2_dedpa scaffold with either [^68^Ga]GaCl_3_ or [^64^Cu]CuCl_2_ but also that the readily accessible chelator for functionalization H_2_L^1^ is a promising platform for proof-of-principle imaging and/or therapy studies, the next step.

### Human Serum Stability Assay with ^68^Ga and ^64^Cu

The stability of metal chelators in radiopharmaceuticals is challenged by endogenous proteins *in vivo*. For instance, apo-transferrin and albumin are well-known to form stable complexes with Ga(III), whereas ceruloplasmin, superoxide dismutase, and metallothioneins can compete for, and displace (transchelate), bound Cu(II). Because human serum contains such endogenous ligands, *in vitro* serum stability challenge assays with [^68^Ga]Ga^3+^ and [^64^Cu]Cu^2+^ complexes at different time points can be predictive indicators of *in vivo* inertness.

To investigate the *in vitro* stability of the complexes formed with either H_2_L^1^ or H_2_L^2^ and [^68^Ga]GaCl_3_, a 1 h stability challenge study was incubated in human serum. Both H_2_L^1^ and H_2_L^2^ gallium complexes were fully intact (>99%) after 1 h in human serum at 37 °C (Figures S73–S75), suggesting that the strategy of providing bifunctionality to the H_2_dedpa scaffold with 3-propylamine (H_2_L^1^) or the simple model with the *N*-propylphthalimide chain (H_2_L^2^) does not negatively impact the inertness of the core [^68^Ga]Ga^3+^-dedpa complexes.

Similarly, human serum stability challenge assays of [^64^Cu][Cu(H_2_L^1^)]^2+^ and [^64^Cu][CuL^2^] also assessed their radiochemical stability *in vitro*. Both [^64^Cu][Cu(H_2_L^1^)]^2+^ and [^64^Cu][CuL^2^] remained intact (>95%) when incubated with serum for 18 h at 37 °C (Figures S76–S78), a marked improvement compared to the parent complex [^64^Cu][Cu(dedpa)],^21^ and is in line with that observed for [Cu(dedpa-*N*,*N*′-propyl-2NI)].^23^ This result supports the finding that conversion of the secondary amines in the ethylenediamine core of depda^2–^ to tertiary amines by anchoring a functionalized propylene chain clearly increases the stability of the corresponding [^64^Cu]Cu^2+^ complexes. Increased stability of [^64^Cu]Cu^2+^ complexes with ligands containing tertiary rather than secondary amines is also documented for TETA derivatives.^29^

## Conclusions

The dedpa^2–^ platform presents a real opportunity for radiopharmaceutical design due to its fast and quantitative radiolabeling with various radiometals at RT. On the basis of this open-chain skeleton, we have built a very promising novel versatile scaffold for use in this field. Our new chelator (denoted as H_2_dedpa-*N*,*N*′-pram, H_2_L^1^) incorporates propylamine chains anchored to the secondary amines of the ethylenediamine backbone of dedpa^2–^, thus providing optimal functional groups to prepare BFCs via conjugation and/or click chemistry. Previous efforts to incorporate the primary alkylamine group into the depda^2–^ core were unsuccessful because the topology was unsuitable.^17^ However, using this tailor-made design strategy, we found an optimal anchoring position of the primary amine group, accompanied by a simple and accessible synthetic route. Likewise, in the study reported herein, we used the H_2_L^2^ platform as a simple structural model for conjugated systems, providing stable coordination of both Cu(II) and Ga(III) metal ions upon conjugation of the primary amines in H_2_L^1^. Solution thermodynamic studies showed that conversion of the secondary amines in the ethylenediamine core of dedpa^2–^ to tertiary amines with two 3-propylamine chains greatly increases the stability of the corresponding Cu(II) complexes (log *K*_ML_ = 23.05(2) for [CuL^1^] and log *K*_ML_ = 22.70(2) for [CuL^2^]; pCu = 21.96 and 22.8, respectively), being higher than those with previous dedpa^2–^ derivatives or those with the commonly used macrocyclic chelators DOTA or NOTA. Higher thermodynamic stability was also achieved for the [GaL^1^]^+^ complex [log *K*_ML_ = 23.80(2); pGa = 19.5] compared to [Ga(dedpa)]^+^ [log *K*_ML_ = 22.9(1); pGa = 22.2] and to that of Ga^III^-DOTA [log *K*_ML_ = 21.33; pGa = 18.5]. X-ray crystallography confirms that the N_4_O_2_ dedpa^2–^ coordination sphere is maintained with both Ga(III) and Cu(II).

Both H_2_L^1^ and H_2_L^2^ are quantitatively radiolabeled with [^68^Ga]Ga^3+^ (>99%) at 10^–5^ M reaction within 15 min at RT and H_2_L^2^ also at 10^–6^ M. The complexes formed show high radiochemical stability in human serum stability assays [intact (>99%) after 1 h in human serum at 37 °C], confirming the potential of H_2_L^1^ for preparing conjugated [^68^Ga]Ga^3+^ PET probes. Moreover, quantitative labeling of both H_2_L^1^ and H_2_L^2^ with [^64^Cu]Cu^2+^ was also observed at 10^–5^ M within 15 min at RT, and the human serum stability assays confirm the high stability *in vitro* as [^64^Cu][Cu(H_2_L^1^)]^2+^ and [^64^Cu][CuL^2^] complexes remained intact (>95%) when incubated with serum for 18 h at 37 °C.

The results herein show the enormous potential of H_2_L^1^ and its conjugates in the development of radiopharmaceuticals based on both [^67/68^Ga]Ga^3+^ and [^64^Cu]Cu^2+^. Conjugation studies are underway with the idea of preparing not only BFCs for PET probes but also scaffolds that can be used in hybrid imaging modalities.

## Experimental Section

### Materials and Methods

All reagents and solvents were purchased from commercial suppliers (Sigma-Aldrich, Fisher Scientific, and Merck) and used as received with the exception of the acetonitrile (ACN) used in synthesis, which was dried according to the usual method.^30^ Reactions were monitored by TLC (Fluka kiesegel, aluminum sheet). Flash chromatography was performed using Reveleris Silica (40 g, 80 g), Redisep Rf Gold High Performance (5.5 g), FlashPure Select C18 (4 g) columns with a CombiFlash Rf machine of Teledyne ISCO. Water was ultrapure (18.2 MΩ/cm at 25 °C, Milli-Q). ^1^H and ^13^C NMR spectroscopies were performed at RT on either Bruker AVANCE 500 MHz or Bruker AV III HD 400 MHz spectrometers at the “Servicios de Apoio á Investigación - SAI” of the Universidad da Coruña (Spain) or on Bruker AV400 and AV600 spectrometers at the University of British Columbia, Vancouver (Canada). Chemical shifts (δ) are quoted in ppm relative to residual solvent peaks as appropriate. Coupling constants (*J*) are provided in hertz (Hz). ^1^H NMR signals are designated as follows: s (singlet), d (doublet), t (triplet), q (quartet), quin (quintet), m (multiplet), or a combination of these, with br representing a broad signal. High-resolution electrospray ionization mass spectrometry (HR-ESI-MS) was performed on a Thermo LTQ-Orbitrap Discovery (SAI, Universidad da Coruña, Coruña, Spain) or using a Waters Micromass LCT TOF instrument at the University of British Columbia, Vancouver (Canada). Results are labeled with *m*/*z* values ([M + X]^±^).

### Synthesis and Characterization

Compounds *tert*-butyl 6-(bromomethyl)picolinate (**2**)^31^ and 1,2-bis[[[(6-methoxycarbonyl)pyridin-2-yl]methyl]amino]ethane (**4**)^32^ were prepared according to the literature.

#### 2,2′-[(1*E*,13*E*)-2,6,9,13-Tetraazatetradeca-1,13-diene-1,14-diyl]diphenol (**1**)

Salicylaldehyde (48.1 mmol, 5 mL) was added dropwise to a solution of 1,5,8,12-tatraazadodecane (21.8 mmol, 4 mL) in methanol (MeOH) at 0 °C. The yellow solution was stirred overnight at RT. Then, the solvent was removed under reduced pressure, and the crude was recrystallized in tetrahydrofuran to give **1** as a yellow solid (yield 50%). ^1^H NMR (400 MHz, CDCl_3_): δ 8.34 (s, 2H), 7.33–7.28 (m, 2H), 7.23 (d, *J* = 7.3 Hz, 2H), 6.94 (d, *J* = 8.2 Hz, 2H), 6.86 (t, *J* = 7.4 Hz, 2H), 3.65 (s, 4H), 2.73 (s, 8H), 1.87 (s, 4H). HR-ESI-MS. Calcd for C_22_H_31_N_4_O_2_ ([M + H]^+^): 383.2442. Found: 383.2436.

#### *N*,*N*′-Bis(3-aminopropyl)-*N*,*N*′-bis[6-(*tert*-butoxycarboxy)pyridin-2-yl]-1,2-diaminoethane (**3**)

Compound **1** (0.52 mmol, 0.201 g) was dissolved in 15 mL of dry ACN. *N*,*N*-Diisopropylethylamine (DIPEA; 1.14 mmol, 0.2 mL) was added, and the mixture was stirred 15 min at RT under argon. Then, compound **2** (1.14 mmol, 0.310 g) was added, and the mixture was stirred at reflux for 24 h under argon. When the reaction was finished [TLC; 9:1 dichloromethane (DCM)/MeOH], the solvent was removed under reduced pressure and the residue was dissolved in 25 mL of DCM. The organic layer was washed with water (3 × 10 mL) and extracted with 0.1 M HCl (3 × 10 mL). The acidic aqueous layer was washed with DCM (2 × 10 mL), and then a saturated aqueous solution of K_2_CO_3_ was added dropwise until pH = 9. The basic aqueous layer was extracted with DCM (2 × 10 mL). The organic layer was filtered, and the solvent was eliminated under reduced pressure to give an orange oil, 0.2316 g. The crude product was purified by column chromatography (Redisep Rf C18 4g, aqueous ammonium bicarbonate (1 g/L)/ACN: 30% ACN for 4 min, from 30% ACN to 40% ACN for 1 min, and 40% ACN for 3 min; *t*_R_ = 5.0 min). Yield: 40%. ^1^H NMR (400 MHz, CDCl_3_): δ 7.87 (dd, *J* = 7.7 and 1.1 Hz, 2H, Ha), 7.75 (t, *J* = 7.7 Hz, 2H, Hb), 7.56 (dd, *J* = 7.8 and 1.1 Hz, 2H, Hc), 3.83 (s, 4H, Hd), 2.80 (t, *J* = 6.7 Hz, 4H, Hh), 2.67 (s, 4H, He), 2.55 (t, *J* = 6.7 Hz, 4H, Hf), 1.70 (quin, *J* = 6.7 Hz, 4H, Hg), 1.61 (s, 18H, Hj). ^13^C NMR (101 MHz, CDCl_3_): δ 164.44 (Ck, C), 160.10 (Cm, C), 148.67 (Cl, C), 137.72 (Cb, CH), 126.37 (Cc, CH), 123.46 (Ca, CH), 82.77 (Cn, C), 60.36 (Cd, CH_2_), 51.68 (Ce, Cf, CH_2_), 40.07 (Ch, CH_2_), 28.23 (Cj, Cg, CH_3_) (see Figure S1 for labels). HR-ESI-MS. Calcd for C_30_H_49_N_6_O_4_ ([M + H]^+^): 557.3810. Found: 557.3802.

#### *N*,*N*′-Bis(propylphthalimide)-*N*,*N*′-bis[6-(*tert*-butoxycarboxy)pyridin-2-yl]-1,2-diaminoethane (**5**)

DIPEA (3.3 mmol, 0.575 mL) was added to a solution of **4** (1.32 mmol, 472 mg) in dry acetonitrile (40 mL), and the resulting yellow solution was stirred 15 min at RT. Then, (3-bromopropyl)phthalimide (2.89 mmol, 775 mg) was added in several portions. The mixture was stirred at reflux 48 h under argon. When the reaction is finished (TLC; 9:1 DCM/MeOH), the mixture was cooled at RT and the solvent concentrated *in vacuo* to give an orange oil, which was removed in DCM. The organic layer was extracted with water (3 × 20 mL) and then dried over anhydrous Na_2_SO_4_. After filtering, the solvent was removed under reduced pressure to give a yellow oil, which was purified by column chromatography (SiO_2_, DCM/MeOH from 0% to 10% MeOH; *t*_R_ = 5.2 min) to afford **5** as a pale-yellow oil (yield 70%). ^1^H NMR (400 MHz, CDCl_3_): δ 7.94 (dd, *J* = 6.5 and 2.3 Hz, 2H, Ha), 7.84–7.76 (m, 4H, Hi,l), 7.77–7.71 (m, 4H, Hb,c), 7.72–7.64 (m, 4H, Hj,k), 3.97 (s, 6H, Hm), 3.80 (s, 4H, Hd), 3.67 (t, *J* = 6.9 Hz, 4H, Hh), 2.63 (s, 4H, He), 2.55 (t, *J* = 6.9 Hz, 4H, Hf), 1.81 (quin, *J* = 6.9 Hz, 4H, Hg). ^13^C NMR (101 MHz, CDCl_3_): δ 168.26 (Cs,t, C), 165.90 (Cn, C), 161.16 (Cp, C), 147.08 (Co, C), 137.37 (Cb,c, CH), 133.86 (Cj,k, CH), 132.13 (Cq,r, C), 126.06 (Cb,c, CH), 123.54 (Ca, CH), 123.16 (Ci,j, CH), 60.82 (Cd, CH_2_), 52.86 (Cm, CH_3_), 52.54 (Ce, CH_2_), 52.38 (Cf, CH_2_), 36.20 (Ch, CH_2_), 26.48 (Cg, CH_2_) (see Figure S6 for labels). HR-ESI-MS. Calcd for C_40_H_41_N_6_O_8_ ([M + H]^+^): 733.2980. Found: 733.2975.

#### *N*,*N*′-Bis(3-aminopropyl)-*N*,*N*′-bis(6-carboxypyridin-2-yl)-1,2-diaminoethane, H_2_dedpa-*N*,*N*′-pram (H_2_L^1^)

Compound **3** was dissolved in a solution of HCl 6 M (5 mL) and stirred at RT for 1 h. After that time, the solvent was evaporated under reduced pressure and the residue was redissolved in water. The process was repeated three times in order to eliminate excess HCl. The residue was lyophilized, obtaining H_2_L^1^ as a dihydrochloride salt (yellowish solid). Yield: ca. 90%. ^1^H NMR (500 MHz, D_2_O): δ 8.12 (d, *J* = 8.0 Hz, 2H, Ha), 8.08 (t, *J* = 7.8 Hz, 2H, Hb), 7.69 (d, *J* = 7.5 Hz, 2H, Hc), 4.77 (s, 4H, Hd), 3.87 (s, 4H, He), 3.51 (t, *J* = 8.2 Hz, 4H, Hf), 3.07 (t, *J* = 7.6 Hz, 4H, Hh), 2.22 (m, 4H, Hg). ^13^C NMR (126 MHz, D_2_O): δ 166.98 (Ci, C), 149.82 (Ck C), 146.42 (Cj, C), 140.39 (Ca, C), 127.42 (Cc, CH), 125.90 (Cb, CH), 57.25 (Cd, CH_2_), 52.66 (Cf, CH_2_), 49.41 (Ce, CH_2_), 36.36 (Ch, CH_2_), 21.75 (Cg, CH_2_) (see Figure S11 for labels). HR-ESI-MS. Calcd for C_22_H_34_N_6_O_4_ ([M + 2H]^2+^): 223.1315. Found: 223.1315.

#### *N*,*N*′-Bis(propylphthalimide)-*N*,*N*′-bis(6-carboxypyridin-2-yl)-1,2-diaminoethane, H_2_dedpa-*N*,*N*′-prpta (H_2_L^2^)

Compound **5** (1.36 g, 1.86 mmol) was dissolved in 10 mL of a 6 M HCl solution in water. The mixture was stirred overnight at reflux, yielding H_2_L^2^ as a dihydrochloride salt (white solid), which was isolated by filtration, washed with a minimum amount of water, and dried under vacuum (yield 50%). ^1^H NMR (400 MHz, D_2_O): δ 8.04 (t, *J* = 7.8 Hz, 2H, Hb), 7.94 (d, *J* = 7.2 Hz, 2H, Ha), 7.81 (m, *J* = 7.0 and 3.6 Hz, 4H, Hj,k), 7.79–7.74 (m, 4H, Hi,j), 7.71 (d, *J* = 7.8 Hz, 2H, Hc), 4.72 (s, 4H, Hd), 3.87 (s, 4H, He), 3.68 (t, *J* = 6.3 Hz, 4H, Hh), 3.41–3.33 (m, 4H, Hf), 2.13 (dquin, *J* = 7.8, 4.9, and 3.4 Hz, 4H, Hg). ^13^C NMR (101 MHz, D_2_O): δ 170.17 (Cs,t, C), 166.99 (Cn, C), 150.21(Cp, C), 146.99 (Co, C), 140.56 (Cb, CH), 134.97 (Cj,k, CH), 130.84 (Cr,q, C), 127.23 (Cc, CH), 125.47 (Ca, CH), 123.48 (Ci,l, CH), 57.46 (Cd, CH_2_), 52.98 (Cf, CH_2_), 49.27 (Ce, CH_2_), 34.65 (Ch, CH_2_), 23.20 (Cg, CH_2_) (see Figure S16 for labels). HR-ESI-MS. Calcd for C_38_H_37_N_6_O_8_ ([M + H]^+^): 705.2667. Found: 705.2642. Single crystals of the formula H_2_L^2^·2HCl·2H_2_O suitable for X-ray diffraction were grown by the slow evaporation of an aqueous solution of H_2_L^2^ (1 × 10^–4^ M) in 1 M HCl.

##### [Cu(H_2_L^1^)]Cl_2_·*n*Sol

Blue single crystals suitable for X-ray diffraction were grown by recrystallization from isopropyl alcohol (*i*PrOH) of the crude solid obtained by the reaction of H_2_L^1^ (0.075 mmol, 50 mg), CuCl_2_ (0.083 mmol, 11.1 mg), and triethylamine (Et_3_N; 0.45 mmol, 63 μL) in MeOH.

##### [CuL^2^]·*n*Sol

Green single crystals suitable for X-ray diffraction were grown by recrystallization from *i*PrOH of the crude solid obtained by the reaction of H_2_L^2^ (0.051 mmol, 50 mg), CuCl_2_ (0.056 mmol, 7.5 mg), and Et_3_N (0.332 mmol, 46.3 μL) in *i*PrOH/MeOH.

##### [GaL^2^](NO_3_)·3.25H_2_O

Colorless X-ray-quality crystals were grown by the slow evaporation of a solution containing H_2_L^2^ (8.4 μmol, 7.22 mg), Ga(NO_3_)_3_·H_2_O (10.22 μmol, 2.8 mg), and H_2_O (2.2 mL). The sample was adjusted to pH ∼ 2 by the addition of HCl.

### X-ray Crystallography

The X-ray-intensity data of a blue platelike crystal of [Cu(H_2_L^1^)]Cl_2_·*n*Sol and of a green prism crystal of [CuL^2^]·*n*Sol were measured on a Bruker D8 VENTURE PHOTON-III C14 κ-geometry diffractometer system equipped with a Incoatec IμS 3.0 microfocus sealed tube (Mo Kα, λ = 0.71073 Å) and a multilayer mirror monochromator. Data were corrected for Lorentz and polarization effects and for absorption using a multiscan method (*SADABS*).^33^ Complex scattering factors were taken from the program *SHELX2019* running under the *WinGX* program system.^34^ For [Cu(H_2_L^1^)]Cl_2_·*n*Sol, the structure was solved and refined using the Bruker *SHELXT*(35) software package, whereas the structure of [CuL^2^]·*n*Sol was solved with *SIR2019*.^36^ Both structures were refined by full-matrix least squares on *F*^2^ with *SHELXL 2019*.^37^ The hydrogen atoms were included in calculated positions and refined in riding mode. In both crystals, we found heavily disordered solvent molecules (water and/or isopropyl alcohol), some of them close to special positions that made it difficult to get a good model for them. For that reason, we decided to perform the *SQUEEZE*(38) procedure under *PLATON*. This procedure takes care of the contribution of a heavily disordered solvent to the calculated structure factors by back-Fourier transformation of the continuous density found in a masked region of the difference map. The masked region is defined as the solvent-accessible region left by the ordered part of the structure. Moreover, the crystal of [CuL^2^]·*n*Sol shows positional disorder for all of the atoms of the complex except those belonging to one phthalimide arm [the occupational factor was 0.814(6) for atoms labeled with A]. Finally, the refinement converged in both crystals with anisotropic displacement parameters for all non-hydrogen atoms.

The X-ray-intensity data of a colorless blade-shaped crystal of [H_2_L^2^]·2HCl·2H_2_O and a colorless prism-shaped crystal of [GaL^2^](NO_3_)·3.25H_2_O were measured on a Bruker APEX II area detector diffractometer, using Cu Kα radiation (a microfocus sealed X-ray tube) for the former and Mo Kα radiation (TRIUMPH monochromator and a sealed X-ray tube) for the latter. The total number of runs and images was based on the strategy calculation from the program *APEX4*. The unit cell was refined using *SAINT*,^39^ and *SADABS*(33) was used for absorption correction. The structure was solved with the *SHELXT 2018/2* solution program^35^ using intrinsic phasing methods and by using *Olex2*, version 1.5, as the graphical interface.^40^ The model was refined with *SHELXL* using full-matrix least-squares minimization on *F*^2^. In both cases, all non-hydrogen atoms were refined anisotropically. For [H_2_L^2^]·2HCl·2H_2_O, most hydrogen-atom positions were calculated geometrically and refined using a riding model, but some N–H and O–H hydrogen atoms were located in difference maps and refined freely. The water hydrogen atoms were located in a difference map; however, they could not be refined; they were placed in calculated positions that appear to be reasonable because they fall on positions consistent with a hydrogen-bonded network. Meanwhile, [GaL^2^](NO_3_)·3.25H_2_O crystallizes with three fully occupied and one partially occupied water sites, forming an extended hydrogen-bonded network. All C–H hydrogen atom positions were calculated geometrically and refined using the riding model; however, all O–H hydrogen atoms were located in difference maps and refined freely. H15A and H15B were located in difference maps, but their isotropic displacement parameters were fixed at 1.5 times that of O15.

The crystal data and details on data collection and refinement are summarized in Table S2.

### Solution Thermodynamics

#### General Procedure

Protonation constants and metal stability constants were determined through a multitechnique approach. Combined potentiometric–spectrophotometric titrations were carried out using a Metrohm Titrando 809 equipped with a Ross combined electrode and a Metrohm Dosino 800. The glass cell containing the solutions to be titrated was maintained at a constant temperature, 25 °C, and connected with an inlet–outlet tube for nitrogen gas (purified through a 10% NaOH solution) to exclude CO_2_ prior to and during the titration. Daily electrode calibrations were carried out at proton ion concentration, and the results were analyzed with the Gran^41^ procedure to obtain the standard potential (*E*°) and the ionic product of water p*K*_w_ at *T* = 25 °C and *I* = 0.16 M NaCl. The calibrations involved HCl standard being titrated with carbonate-free titrant NaOH(aq) (0.15 M), and the ionic strength was maintained constant to 0.16 M by the addition of a NaCl solution. The NaOH was previously standardized against freshly dried potassium hydrogen phthalate crystals. Copper and gallium metal-ion solutions used in metal complexation experiments were prepared by the dilution of AA standards. The exact amount of acid present was determined by the titration of equimolar solutions of either Cu(II) or Ga(III) and Na_2_H_2_EDTA using Gran plotting.^41^

#### Protonation Constants of H_2_L^1^ and H_2_L^2^

Protonation equilibria of either H_2_L^1^ or H_2_L^2^ were studied by titrations of solutions containing [H_2_L^1^] = 9.56 × 10^–4^ M or [H_2_L^2^] = 5.87 × 10^–4^ M at 25 °C and 0.16 M NaCl ionic strength using a potentiometric–spectrophotometric procedure. In each titration (100–150 equilibrium points and pH range 2–11), the electromotive force values were recorded after 2 min of each NaOH addition and the spectrophotometer was synchronized to obtain a UV spectrum for each pH data point. Spectra were recorded in the 200–400 nm wavelength range with a 0.2-cm-path-length optic dip probe connected to a Varian Cary 60 UV–vis spectrophotometer. The obtained spectrophotometric and potentiometric data were analyzed with *HypSpec2014*(42) and *HyperQuad2013*(43) to obtain the protonation constants and molar absorptivities of the different absorbing species of the ligands (Table 3 and Figures S42–S49 and S51B). Note that H_2_L^2^ at pH > 9 was observed to hydrolyze, marked by the disappearance of the phthalimide band at λ = 300 nm; therefore, the data above that pH were excluded from the calculations. Additional ^1^H NMR titrations were carried out for H_2_L^1^ to better understand the protonation sequence and their assignments (Figures S45 and S46). A set of H_2_L^1^ solutions (4 × 10^–3^ M) in D_2_O were prepared by the addition of DCl or NaOD, and their ^1^H NMR spectra were recorded. The pH values of the samples were then measured with a microelectrode (Mettler Toledo), which was calibrated daily at H^+^ concentration as described above. The pH was corrected for the deuterium isotopic effect (pD = pH_reading_ + 0.4).^44^

#### Complex Formation Equilibria with Cu(II) and Ga(III)

Complex formation equilibria of either Ga(III) or Cu(II) with H_2_L^1^ were studied using two different methods. First, in-batch acidic UV–vis spectrophotometric measurements (*l* = 1 cm) were carried out on a set of aqueous solutions containing 1:1 metal-to-ligand molar ratios ([H_2_L^1^ = [Ga^3+^] = 1 × 10^–4^ M; [H_2_L^1^] = [Cu^2+^] = 6.37 × 10^–4^ M at 25 °C and *I* = 0.16 M (NaCl) when possible because the ionic strength was not constant in the samples that required higher acidities to show free metal in solution). The pH in the most acidic samples was calculated from the H^+^ concentration when the pH was below the electrode threshold. From very acidic pH, both Cu(II) and Ga(III) complexes formed, and the first protonated complex species ([Cu(H_3_L^1^)]^3+^ and [Ga(H_2_L^1^)]^3+^) were determined through the fitting of these experiments with the *HypSpec2014* program^42^ (Figures S50–S52 and S56–S58 and Table S3). Further potentiometric titrations were carried out ([H_2_L^1^ = [Ga^3+^] = 8.92 × 10^–4^ M; [H_2_L^1^] = [Cu^2+^] = 8.99 × 10^–4^ M at 25 °C and *I* = 0.16 M (NaCl)), allowing determination of the stability constants in Table S3 and the speciation plots in Figure S62 using the *HyperQuad2013*(43) and *HySS*(45) programs, respectively. Dissociation constants corresponding to the hydrolysis of Ga(III) and Cu(II) aqueous ions included in the calculations were taken from Baes and Mesmer.^46^ Complexation of H_2_L^1^ with Ga(III) was further corroborated by the findings of ^1^H NMR experiments at different pD values (Figures 3 and S26). Similarly, complexation of H_2_L^2^ with Cu(II) or Ga(III) was studied by in-batch acidic UV experiments in a set of solutions prepared with the experimental conditions [H_2_L^2^] = [Ga^3+^] = 1 × 10^–4^ M; [H_2_L^2^] = [Cu^2+^] = 7 × 10^–4^ M, and the pH was adjusted by the addition of different amounts of standardized HCl. The ionic strength was maintained constant at 0.16 M when possible by the addition of NaCl. The first-formed complex species ([Cu(HL^2^)]^+^, [CuL^2^], and [GaL^2^]^+^) were determined through fitting of the experimental data (Figures S53–S55 and S59–S61 and Table S3). The main difference from the procedure used for the complexation studies of H_2_L^1^ is that, although we also performed combined potentiometric–spectrophotometric experiments with both Cu(II) and Ga(III) and H_2_L^2^, fitting of the potentiometric–spectrophotometric data was not possible because the H_2_L^2^ ligand hydrolyzes at basic pH and, therefore, it is not an equilibrium reaction anymore. Nonetheless, our experiments (Figures S61 and S63) show that, in the case of Cu(II), even though the phthalimide moieties in the [CuL^2^] species hydrolyze from pH 9.57, metal complexation is maintained at least up to pH 11, most likely as the [CuL^3^]^2–^ species; for Ga(III) complexation with H_2_L^2^, the phthalimide groups in [GaL^2^]^+^ start to hydrolyze at pH > 7.9 (Figures S55 and S63), and through ^1^H NMR titrations, it is clear that the predominant species at pD = 8.4 is [GaL^3^]^−^ (Figures 2 and S31 and S36).

#### Proton-Assisted Dissociation Kinetics

Proton-assisted dissociation experiments were carried out by spectrophotometric measurements of two sets of solutions containing either H_2_L^1^ or H_2_L^2^ and Ga(III) in a 1:1 molar ratio ([Ga^3+^] = [L] = 1 × 10^–4^ M) incubated in 5 M HCl. The decrease of the bands at λ = 270 nm was followed over 24 h at 15 min time intervals (25 °C and *l* = 1 cm).

### ^68^Ga and ^64^Cu Radiolabeling

#### Materials

[^68^Ga]GaCl_3_ was obtained at BC Cancer from an Eckert & Ziegler IGG100 ^68^Ga generator constructed of a titanium dioxide sorbent that was charged with ^68^Ge and purified according to published procedures.^47^ [^64^Cu]CuCl_2_ was purchased from the University of Alabama as a 0.05 M HCl solution and used without any further purification. The human serum was purchased frozen from Sigma-Aldrich. Analysis of the radiolabeled compounds was performed with either instant thin-layer chromatography (iTLC)-SA (silicic acid-impregnated) or iTLC-SG (silica gel-impregnated) paper plates.

#### General Radiolabeling Procedure

H_2_L^1^ and H_2_L^2^ were dissolved in ultrapure deionized water to obtain a 10^–3^ M stock solution of each ligand. An aliquot of each stock solution was used to give dilutions ranging from 10^–4^ to 10^–6^ M of both H_2_L^1^ and H_2_L^2^. Radiolabeling studies with [^68^Ga]Ga^3+^ were performed in duplicate at final ligand concentrations of 10^–4^–10^–7^ M (20 mL) in NaOAc buffer (2 M, pH = 7.4, 160 mL) using 7.4 MBq of [^68^Ga]Ga^3+^ (20 mL). Radiolabeling studies with [^64^Cu]Cu^2+^ were performed in duplicate at final ligand concentrations of 10^–4^–10^–7^ M (5 mL) in NaOAc buffer (0.5 M, pH = 7, 43 mL) using 0.66 MBq of [^64^Cu]Cu^2+^ (2 mL). The RCC (%) for each reaction was determined after 15 min at RT using radio-TLC with 50 mM EDTA (pH 5) as the mobile phase and iTLC-SG plates for [^68^Ga]Ga^3+^ reactions and iTLC-SA plates for [^64^Cu]Cu^2+^ reactions.

#### Human Serum Stability

An aliquot of either a H_2_L^1^ or H_2_L^2^ stock solution (10^–3^ M for H_2_L^1^ and 10^–5^ M for H_2_L^2^, 40 μL) was added to NaOAc buffer (2 M, pH = 7.4, 320 μL), followed by an aliquot of [^68^Ga]Ga^3+^ (40 μL, 11 MBq). For ^64^Cu human serum stability assay, an aliquot of either a H_2_L^1^ or H_2_L^2^ stock solution (10^–4^ M, 10 μL) was added to NaOAc buffer (0.5 M, pH = 7, 86 μL), followed by an aliquot of [^64^Cu]Cu^2+^ (4 μL, 1.32 MBq). The reactions were left for 15 min at RT before being split into two vials, to each of which was added an equal volume of human serum (200 μL for [^68^Ga]Ga^3+^ and 50 μL for [^64^Cu]Cu^2+^). Serum stability reactions were incubated at 37 °C for 1 h for [^68^Ga]Ga^3+^ and for 1, 2, and 18 h for [^64^Cu]Cu^2+^ before an aliquot was taken for analysis. The percent of intact complex for both ligands was determined via radio-TLC using the same conditions as those above.

## Supporting Information Available

X-ray crystallographic data and selected angles of the coordination spheres of [GaL^2^]^+^, [CuL^2^], and [Cu(H_2_L^1^)]^2+^, NMR spectra (^1^H, ^13^C, DEPT, ^1^H–^1^H COSY, HSQC, HMBC) of intermediates **3** and **5**, chelators H_2_L^1^, H_2_L^2^, and (L^3^)^4–^, and Ga(III) complexes, HR-ESI-MS spectra of intermediates **3** and **5** and chelators H_2_L^1^, H_2_L^2^, and (L^3^)^4–^, solution titration studies, proton-assisted dissociation kinetics, concentration-dependent radiolabeling, and human serum stability data (PDF)
