# Supplementary material for: Toward 68Ga and 64Cu Positron Emission Tomography Probes: Is H2dedpa-N,N′-pram the Missing Link for dedpa Conjugation?
Source: Inorg Chem. 2023 Jan 20;62(50):20593–607. doi: 10.1021/acs.inorgchem.2c04123 (PMC10731644; doi:10.1021/acs.inorgchem.2c04123)
Supplement: Supplementary file 1 — ic2c04123_si_001.pdf [file ic2c04123_si_001.pdf]

Supporting Information

Toward ^{68}Ga, ^{64}Cu PET Probes: Is H_2dedpa-*N*,*N'*-pram the Missing Link for dedpa Conjugation?

Celia Pena-Bonhome,[†] *Desiree Fiaccabrino,*[‡,§] *Tamara Rama,*[†]

Daniel Fernández-Pavón,[†] *Lily Southcott,*[‡,§] *Zhengxing Zhang,*[¥] *Kuo-Shyan Lin,*[¥,⊥]

Andrés de Blas,[†] *Brian O. Patrick,*[¬] *Paul Schaffer,*[§,⊥,#] *Chris Orvig,*[‡]

María de Guadalupe Jaraquemada-Peláez[‡,*] *and Teresa Rodríguez-Blas*[†,*]

[†] Grupo METMED, Departamento de Química, Universidade da Coruña, Campus da Zapateira s/n 15071 A Coruña, Spain.

[‡] Medicinal Inorganic Chemistry Group, Department of Chemistry, University of British Columbia, Vancouver BC, V6T 1Z1, Canada.

[§] Life Sciences Division, TRIUMF, 4004 Wesbrook Mall, Vancouver BC, V6T 2A3, Canada.

[¥] Department of Molecular Oncology, BC Cancer Research Institute, Vancouver, BC, V5Z 1L3, Canada.

[⊥] Department of Radiology, University of British Columbia, Vancouver, BC V5Z 1M9, Canada.

[¬] Department of Chemistry, University of British Columbia, Vancouver BC, V6T 1Z1, Canada.

[#] Department of Chemistry, Simon Fraser University, Burnaby, BC V5A 1S6, Canada.

Table of Contents

X-ray Crystallography Data **S3**

NMR Spectroscopy **S5**

NMR spectra of compound 3 .... S5

NMR spectra of compound 5 .... S8

NMR spectra of H_2L^1 .... S11

NMR spectra of H_2L^2 .... S14

NMR spectra of $(L^3)^{4-}$ S17

NMR Gallium (III) complexation with H_2L^1 .... S20

NMR Gallium (III) complexation with H_2L^2 .... S23

Mass Spectrometry of Compounds 3, 5, H_2L^1 and H_2L^2 .... **S26**

Solution Thermodynamics **S29**

Protonation Constants of H_2L^1 and H_2L^2 .... S29

Stability constants of H_2L^1 and H_2L^2 with Cu(II) and Ga(III) S38

Proton Assisted Dissociation Kinetics S51

^{68}Ga and ^{64}Cu Radiolabeling Data **S53**

Concentration Dependent Radiolabeling S53

Human Serum Stability Data S57

References **S60**

X-ray Crystallography Data

Table S1. Selected angles of the coordination spheres of $[GaL^2]^+$, $[CuL^2]$, and $[Cu(H_2L^1)]^{2+}$.

	$[GaL^2]^+$	$[CuL^2]$	$[Cu(H_2L^1)]^{2+}$
N4- M -O3	79.92(4)	79.85(9)	79.60(15)
N4- M -N3	77.78(4)	77.47(13)	78.86(16)
N1- M -N3	102.67(4)	102.62(19)	109.12(15)
N4- M -O1	97.85(4)	101.30(13)	100.84(14)
N1- M -O1	80.23(4)	80.20(9)	77.97(14)
N1- M -N2	77.92(4)	77.37(9)	78.44(14)
O1- M -N2	156.40(3)	155.05(11)	153.65(13)
O3- M -N3	155.41(3)	154.13(11)	156.99(13)
N1- M -N4	178.03(4)	178.49(14)	171.97(17)

Table S2. Selected crystallographic data.

Data	$H_2L^2 \cdot 2HCl \cdot 2H_2O$	$[Cu(H_2L^1)]Cl_2 \cdot nSol$[a]	$[Cu(L^2)] \cdot nSol$[a]	$[Ga(L^2)](NO_3) \cdot 3.25H_2O$
**Empirical formula**	$C_{38}H_{42}Cl_2N_6O_{10}$	$C_{22}H_{34}Cl_2CuN_6O_5$	$C_{38}H_{34}CuN_6O_8$	$C_{38}H_{40.49}GaN_7O_{14.24}$
**Formula weight**	813.67	596.99	766.25	892.90
**Temperature/K**	90(2)	100(2)	100.0(1)	90(2)
**Crystal system**	Triclinic	Monoclinic	Triclinic	Triclinic
**Space group**	P-1	C2/c	P-1	P-1
**a/Å**	7.4281(4)	26.7132(18)	11.8957(8)	11.0844(7)
**b/Å**	7.9034(4)	13.0377(9)	13.6968(9)	11.4079(7)
**c/Å**	16.6148(7)	19.4697(12)	14.9527(8)	16.8994(10)
**α/°**	93.226(3)	90	94.749(3)	108.031(2)
**β/°**	90.841(3)	101.164(3)	110.105(3)	101.385(2)
**γ/°**	108.035(3)	90	113.393(3)	98.023(2)
**Volume/Å^3**	925.49(8)	6652.6(8)	2030.7(2)	1945.3(2)
**Z**	1	8	2	2
**ρ_{calc} g/cm^3**	1.460	1.192	1.253	1.524
**μ/mm^{-1}**	2.161	0.853	0.592	0.788
**F(000)**	426	2488	794	925
**Crystal size/mm^3**	0.21 × 0.05 × 0.02	0.06 x 0.05 x 0.01	0.34 x 0.24 x 013	0.17 × 0.16 × 0.07
**Θ range for data collection/°**	2.665 to 59.978	2.132 to 27.176	2.047 to 33.142	1.311 to 30.668
**Index ranges**	-8<=h<=8, -8<=k<=8, -18<=l<=18	-34<=h<=34, -16<=k<=16, -24<=l<=24	-18<=h<=18, -21<=k<=21, -23<=l<=23	-15<=h<=15, -16<=k<=16, -24<=l<=24
**Reflections collected**	13805	80362	263641	72132
**Independent reflections**	2672 [R(int) = 0.0602]	7368 [R(int) = 0.1224]	15467 [R(int) = 0.0532]	11963 [R(int) = 0.0283]
**Data/restraints/ parameters**	2672/0/264	7368/20/333	15467/285/798	11963/2/582
**Goodness-of-fit on F^2**	1.056	1.063	1.028	1.032
**Final R indexes [I>=2σ (I)]**	R1 = 0.0475, wR2 = 0.1226	R1 = 0.0806, wR2 = 0.1878	R1 = 0.0317, wR2 = 0.0829	R1 = 0.0277, wR2 = 0.0653
**Final R indexes [all data]**	R1 = 0.0629, wR2 = 0.1336	R1 = 0.0982, wR2 = 0.1975	R1 = 0.0426, wR2 = 0.0875	R1 = 0.0355, wR2 = 0.0685
**Largest diff. peak/hole / e Å^{-3}**	0.620 and -0.344	1.260 and -1.312	0.576 and -0.601	0.631 and -0.255

[a] In both crystals heavily disordered solvent molecules (water and/or isopropanol) were found, some of them close to special positions which made difficult to get a good model for them and the squeeze procedure under Platon was performed.

NMR Spectroscopy

NMR spectra of compound 3

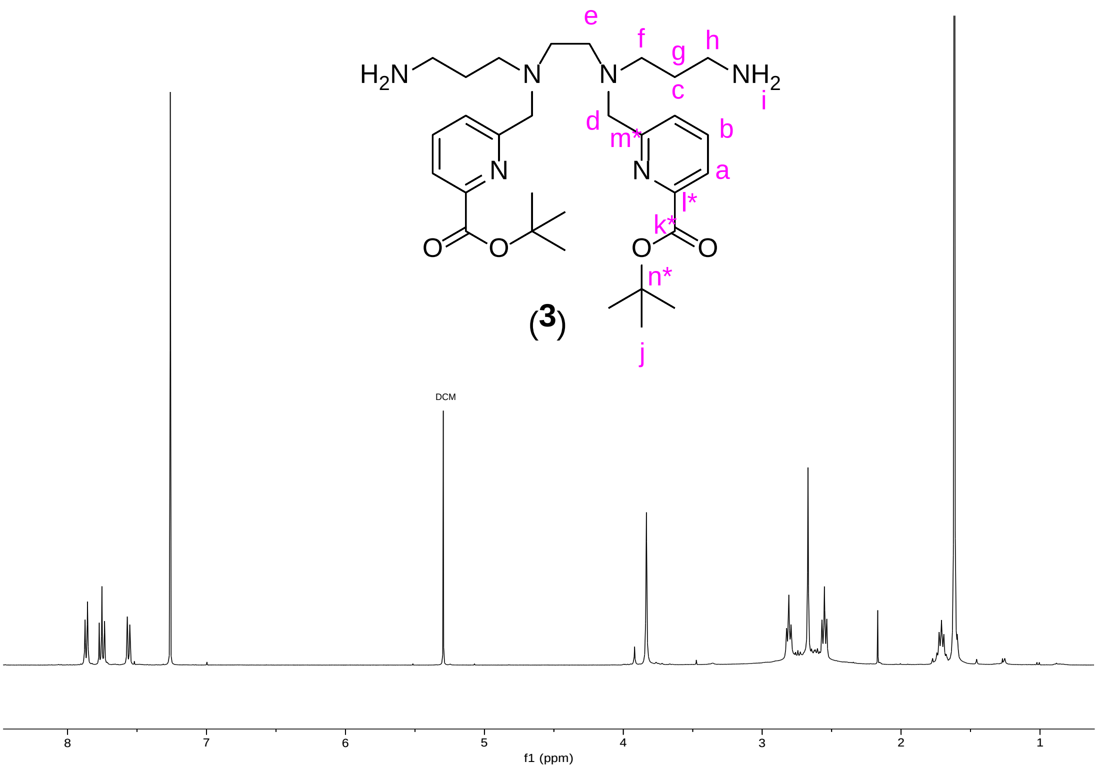

Figure S1. ^{1}H NMR spectrum of compound **3** (400 MHz, 25 °C, $CDCl_3$).

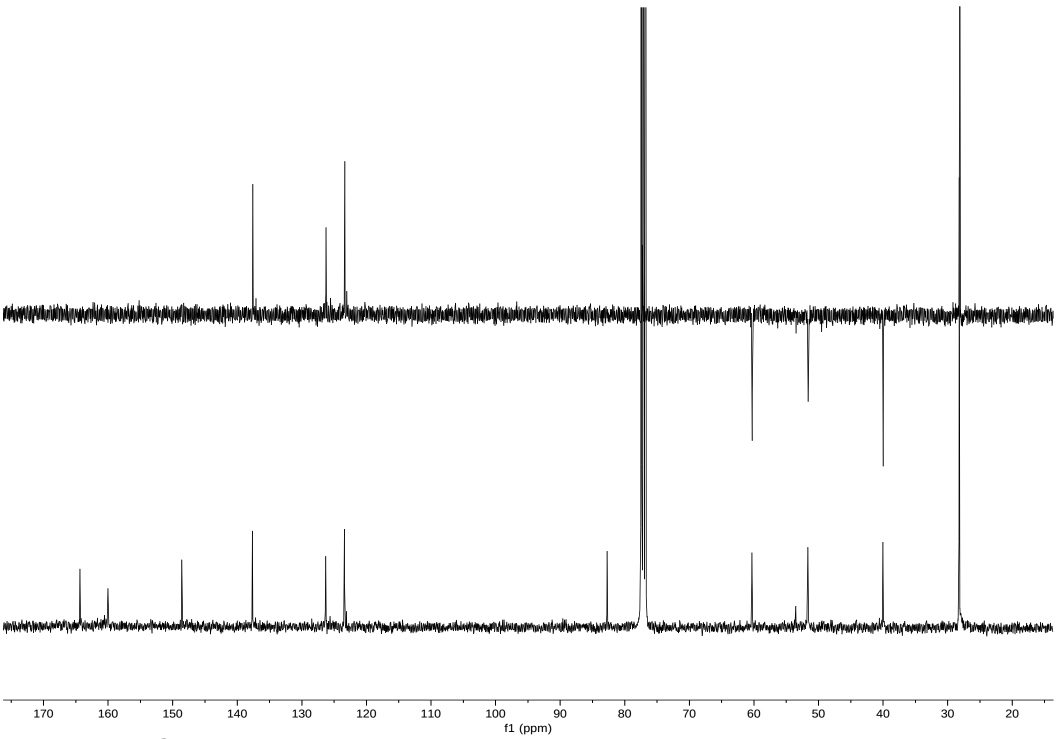

Figure S2. ^{13}C NMR and DEPT spectra of compound **3** (101 MHz, 25 °C, $CDCl_3$).

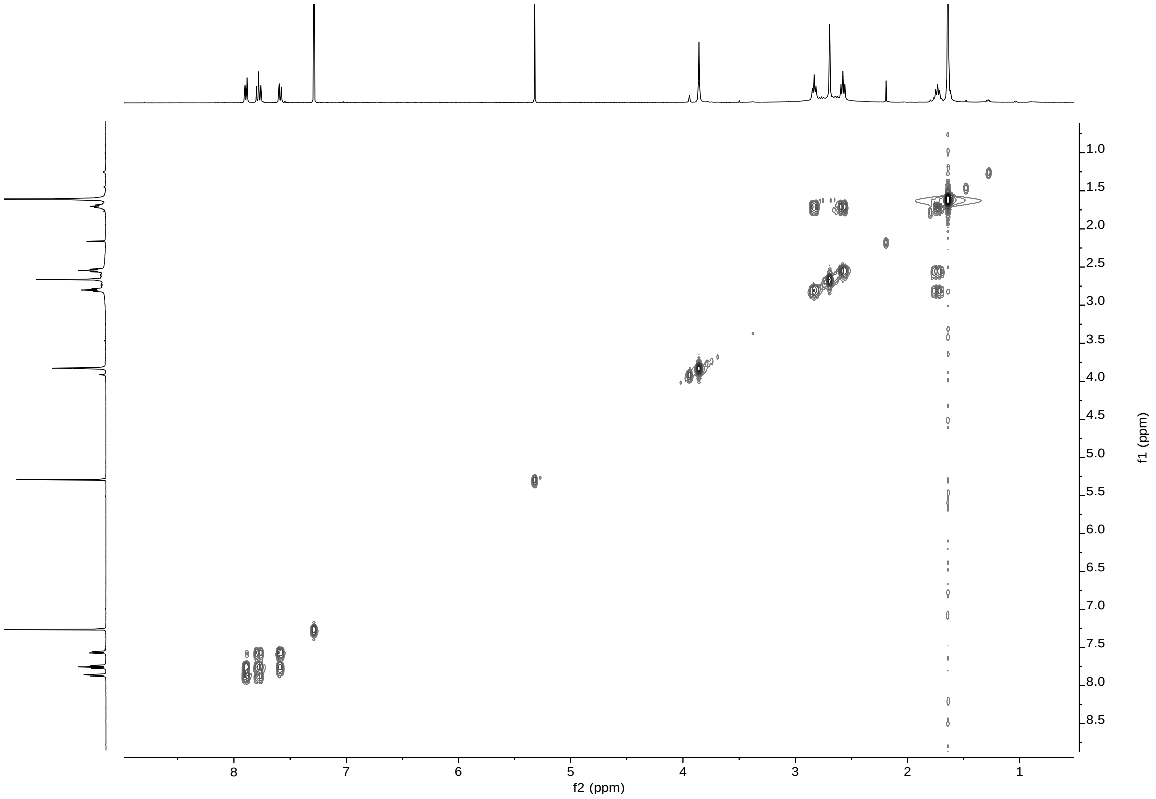

Figure S3. ^{1}H-^{1}H COSY NMR of compound **3** (400 MHz, 25 °C, $CDCl_3$).

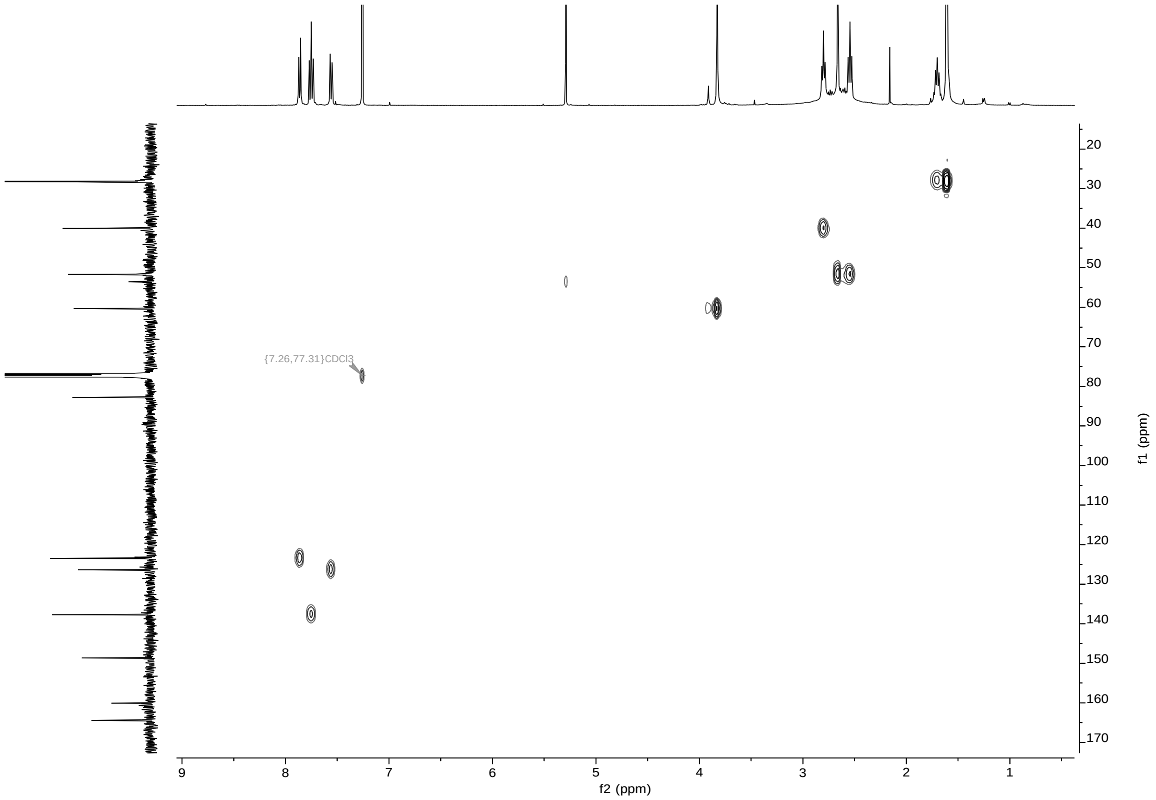

Figure S4. HSQC NMR spectrum of compound **3** (400 MHz, 25 °C, $CDCl_3$).

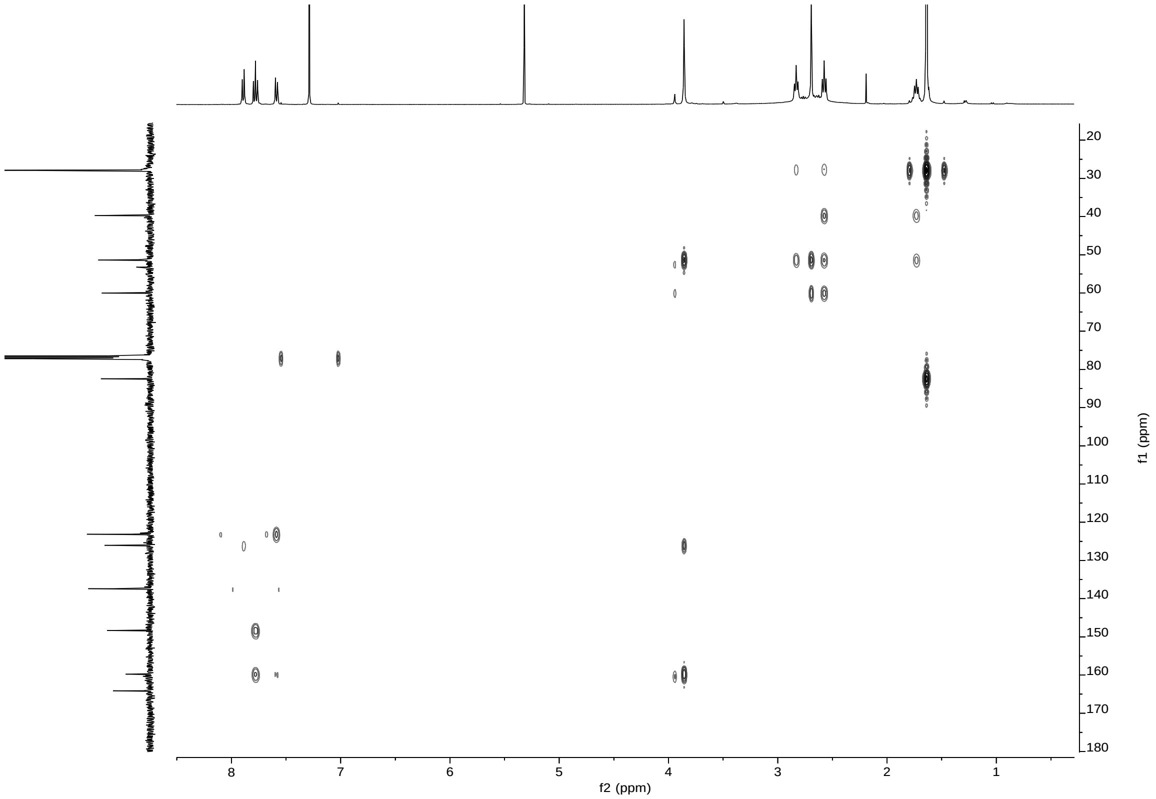

Figure S5. HMBC NMR spectrum of compound **3** (400 MHz, 25 °C, $CDCl_3$).

NMR spectra of compound 5

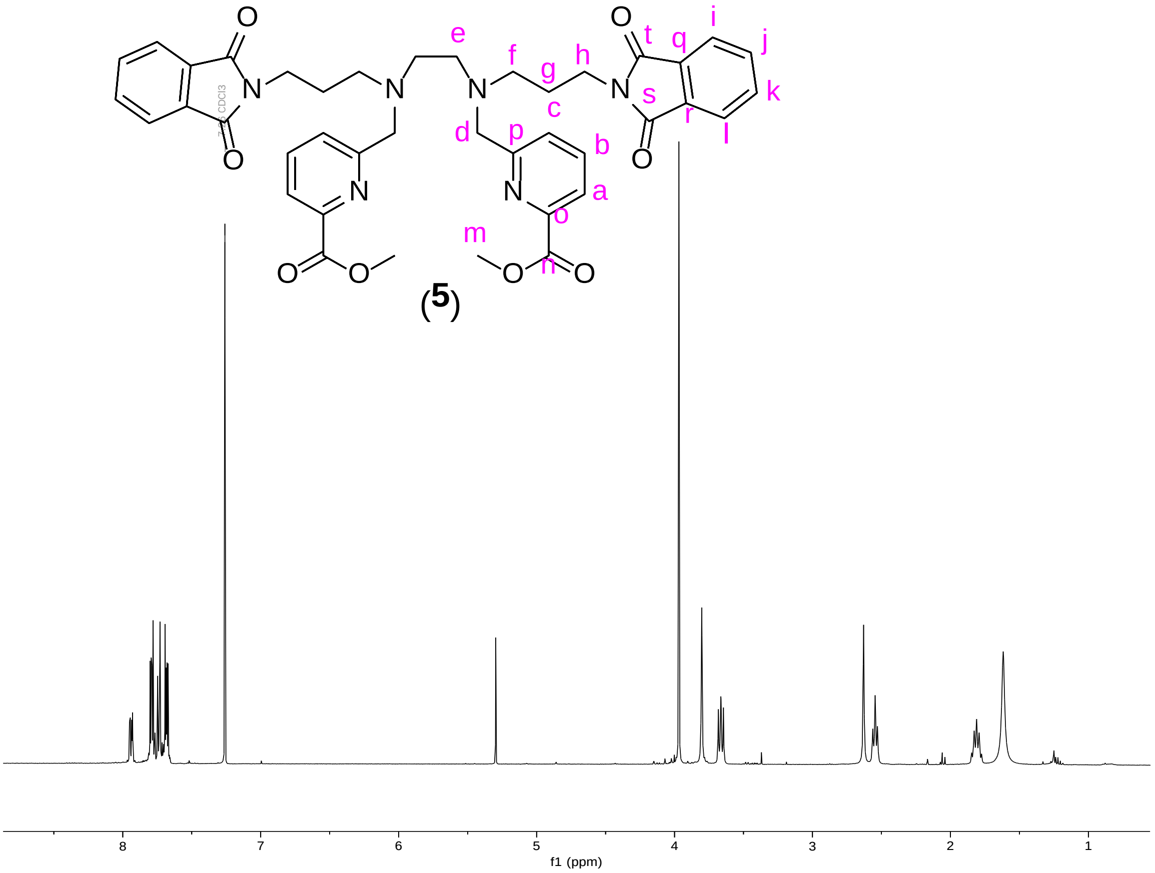

Figure S6. ^{1}H NMR spectrum of compound **5** (400 MHz, 25 °C, $CDCl_3$).

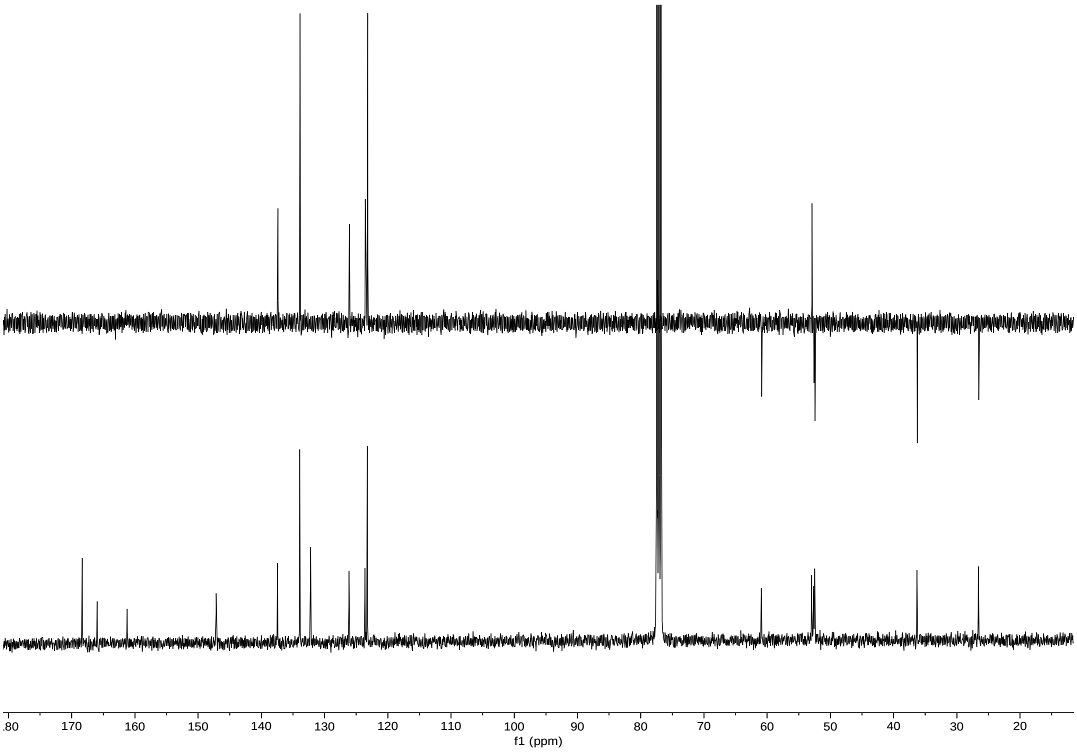

Figure S7. ^{13}C NMR and DEPT spectra of compound **5** (101 MHz, 25 °C, $CDCl_3$).

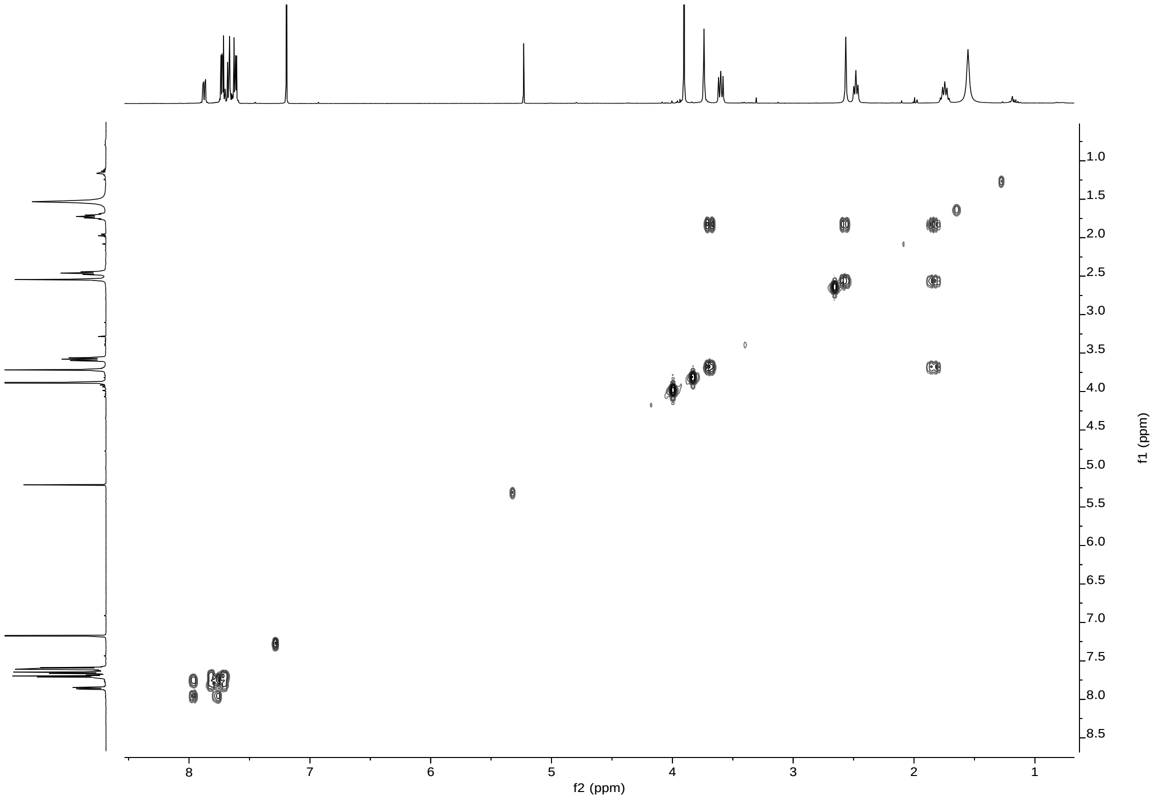

Figure S8. ^{1}H-^{1}H COSY NMR of compound **5** (400 MHz, 25 °C, D_2O).

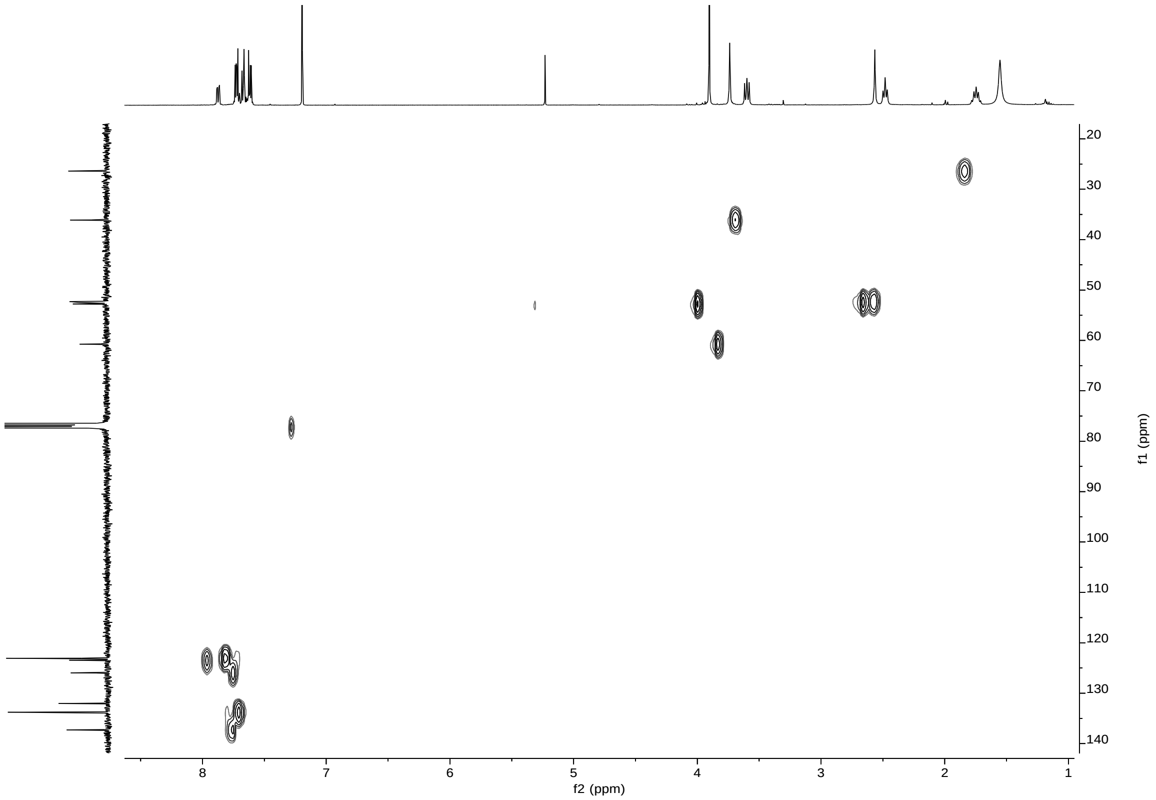

Figure S9. HSQC NMR spectrum of compound **5** (400 MHz, 25 °C, $CDCl_3$).

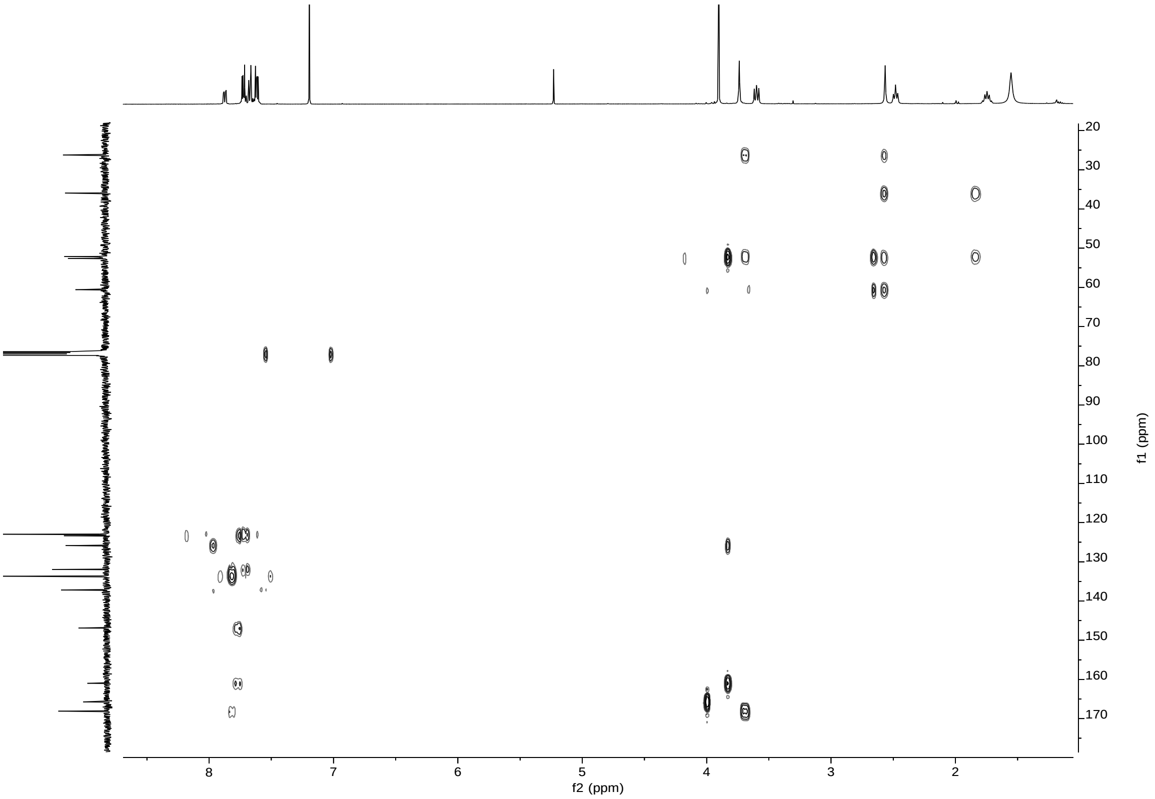

Figure S10. HMBC NMR spectrum of compound **5** (400 MHz, 25 °C, $CDCl_3$).

NMR spectra of H_2L^1

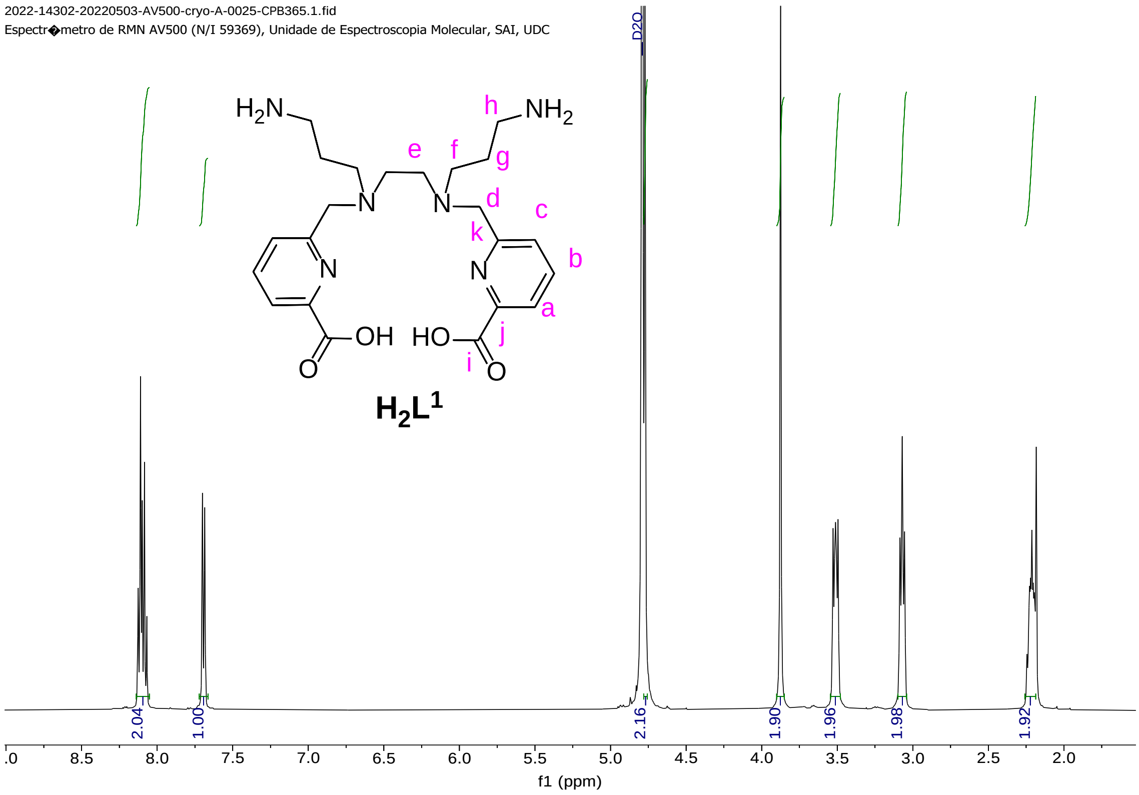

Figure S11. ^{1}H NMR spectrum of $\mathbf{H_2L^1}$ (500 MHz, 25 °C, D_2O).

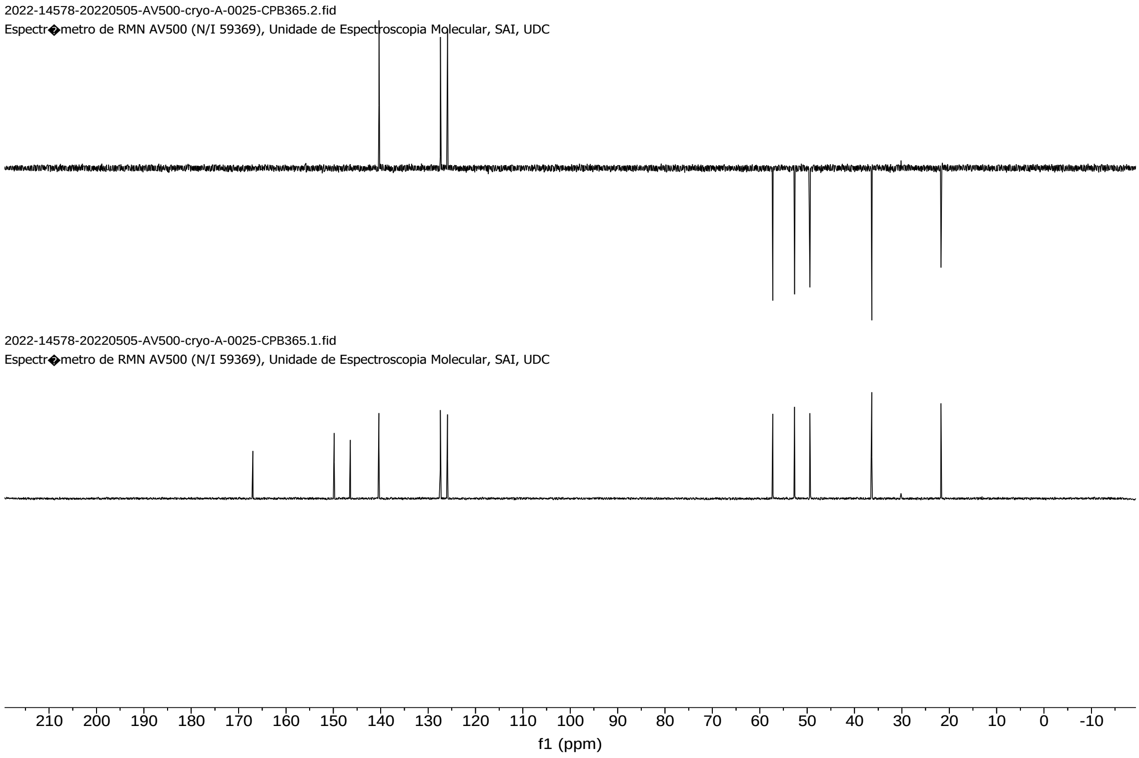

Figure S12. ^{13}C NMR and DEPT spectra of $\mathbf{H_2L^1}$ (126 MHz, 25 °C, D_2O).

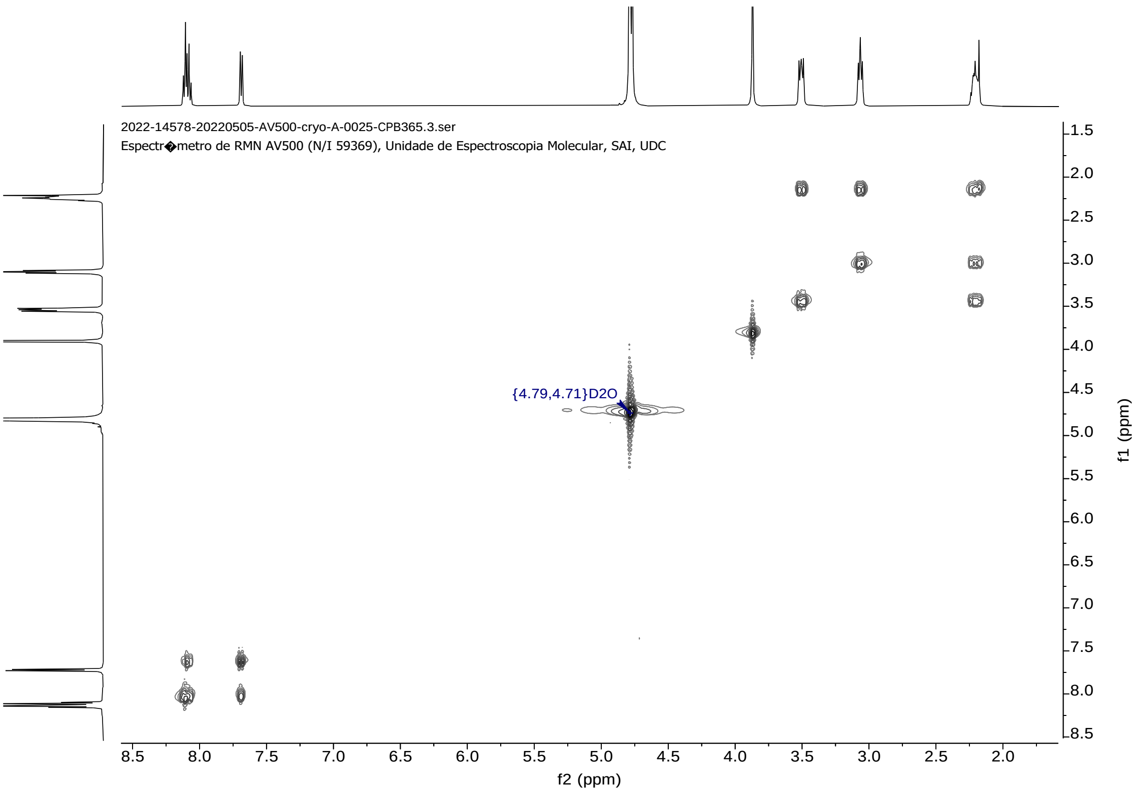

Figure S13. ^{1}H-^{1}H COSY NMR of $\mathbf{H_2L^1}$ (500 MHz, 25 °C, D_2O).

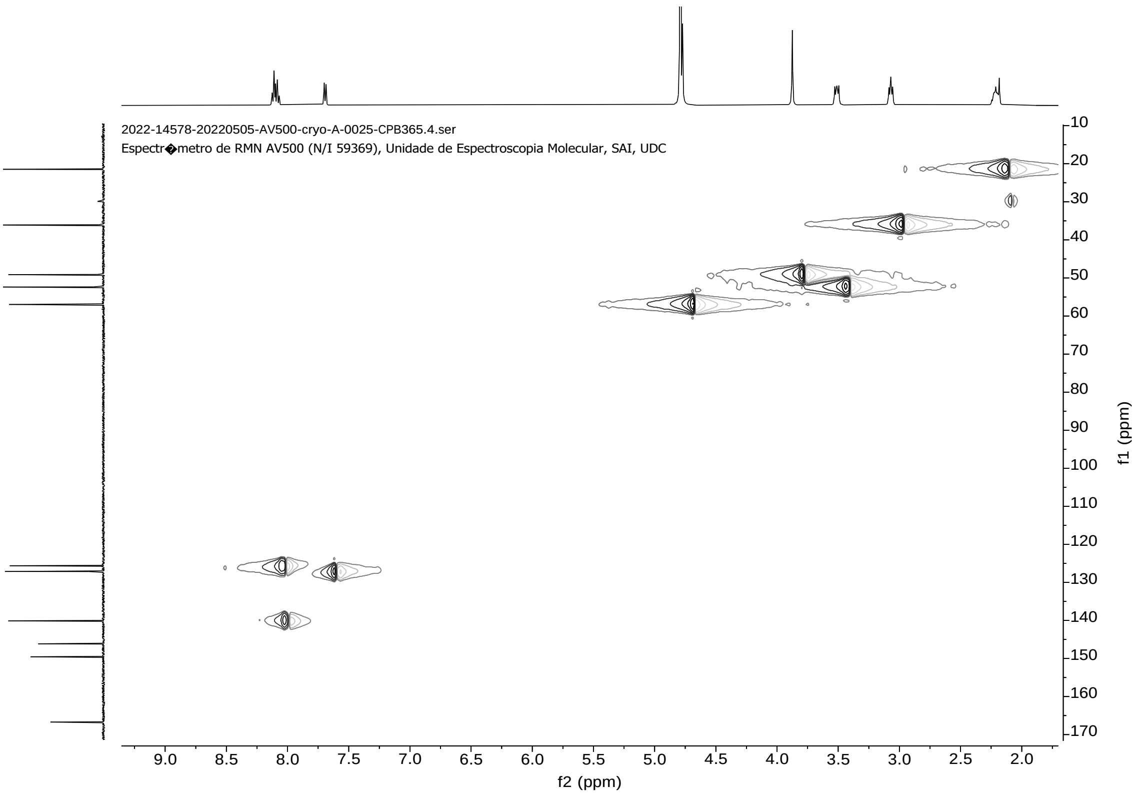

Figure S14. HSQC NMR spectrum of $\mathbf{H_2L^1}$ (500 MHz, 25 °C, D_2O).

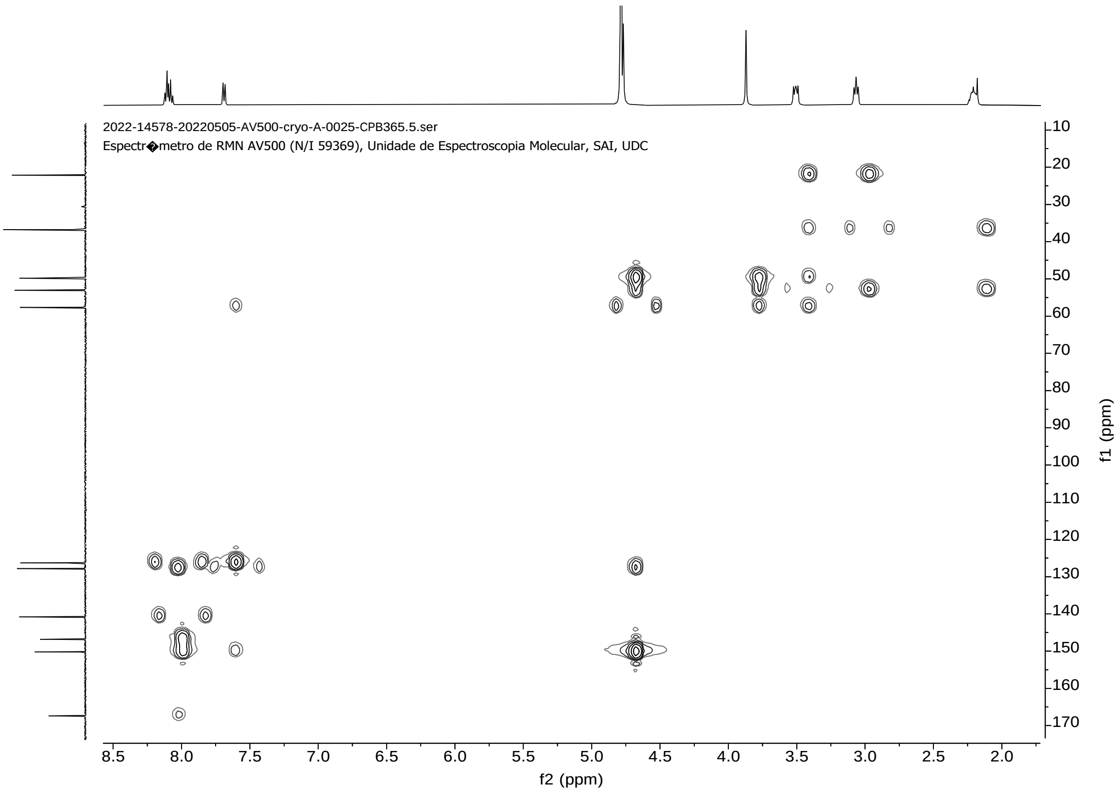

Figure S15. HMBC NMR spectrum of $\mathbf{H_2L^1}$ (500 MHz, 25 °C, D_2O).

NMR spectra of H_2L^2

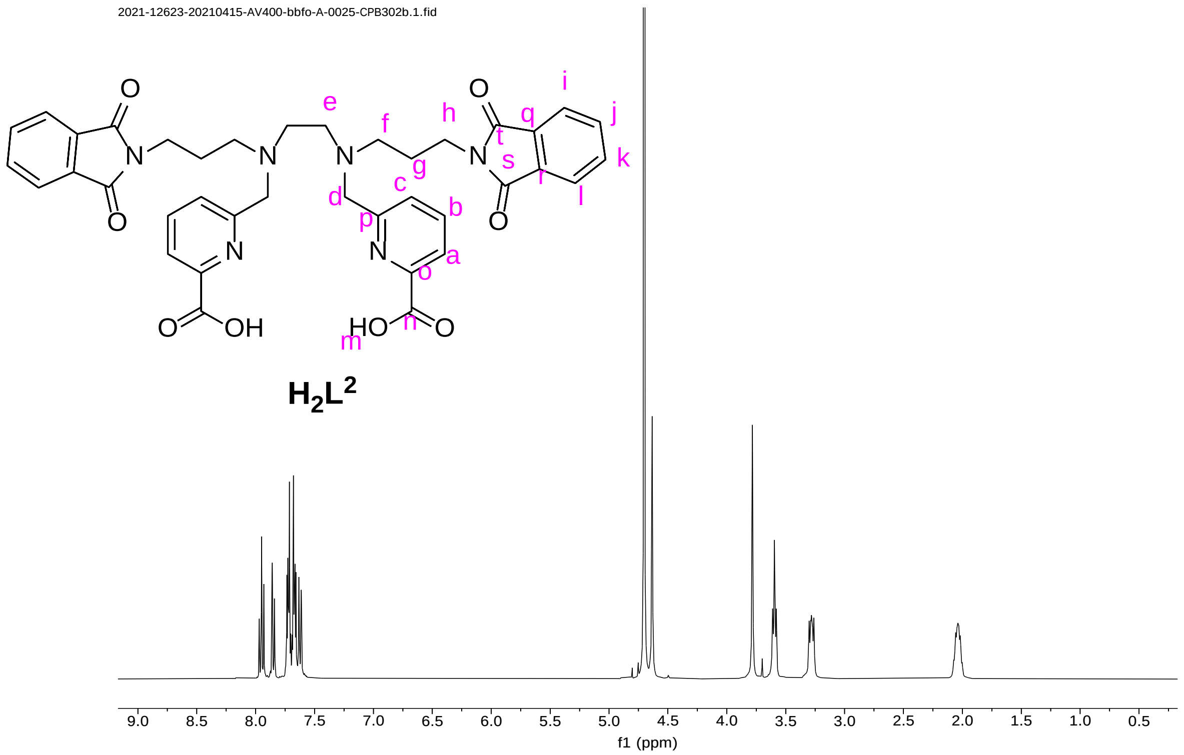

Figure S16. ^{1}H NMR spectrum of $\mathbf{H_2L^2}$ (400 MHz, 25 °C, D_2O).

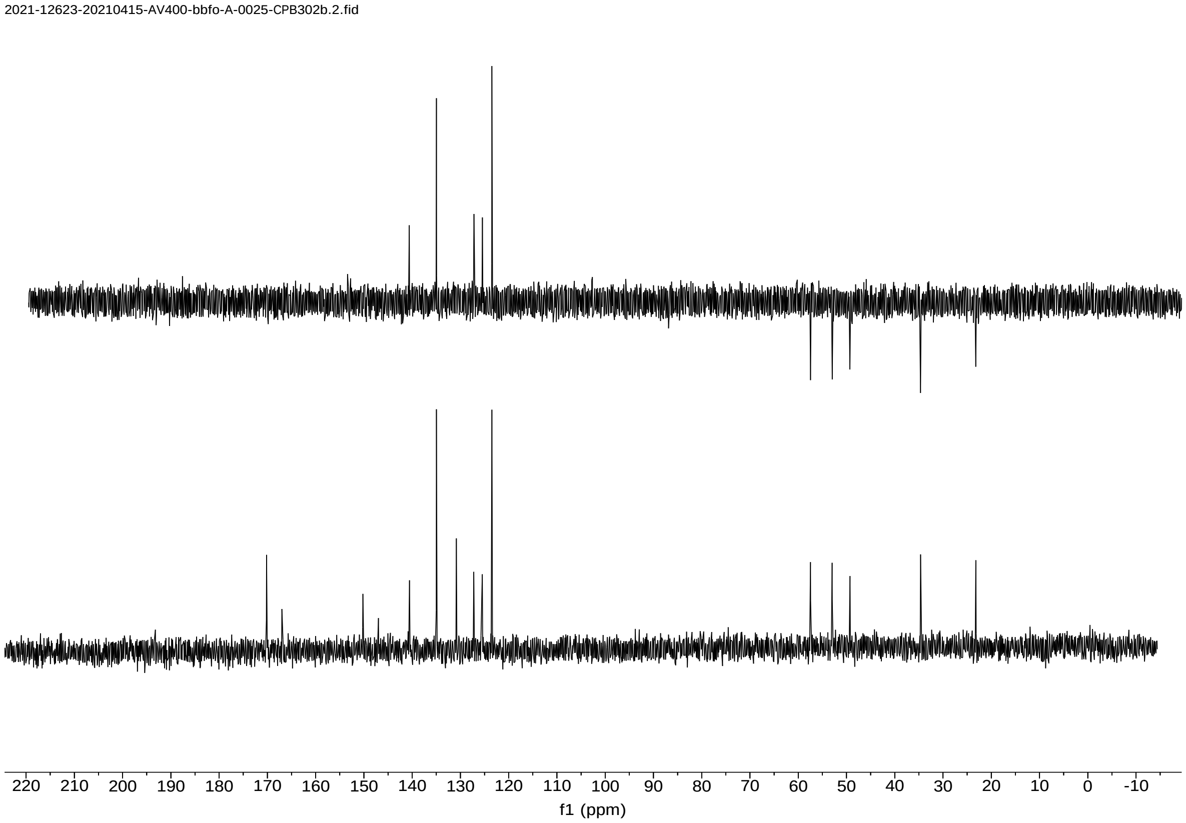

Figure S17. ^{13}C NMR and DEPT spectra of $\mathbf{H_2L^2}$ (101 MHz, 25 °C, D_2O).

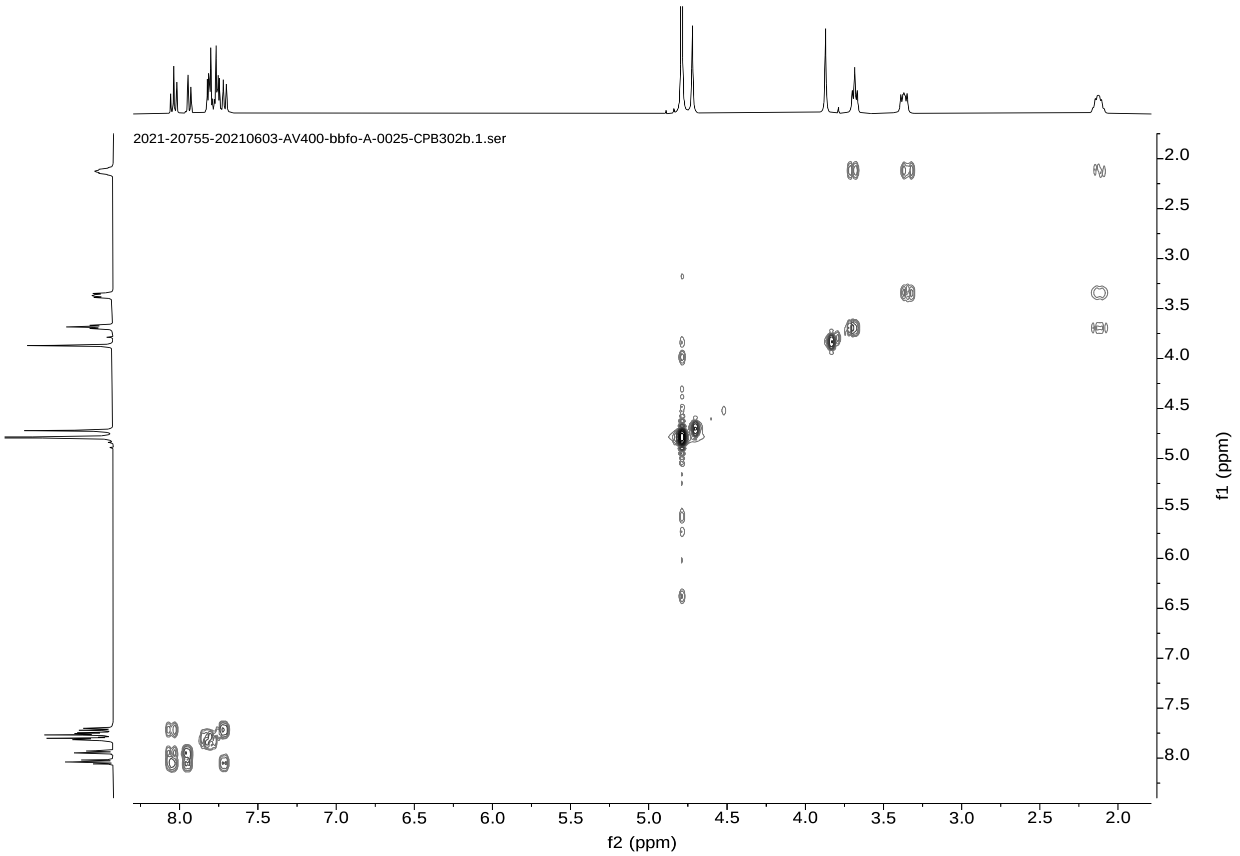

Figure S18. ^{1}H-^{1}H COSY NMR of $\mathbf{H_2L^2}$ (400 MHz, 25 °C, D_2O).

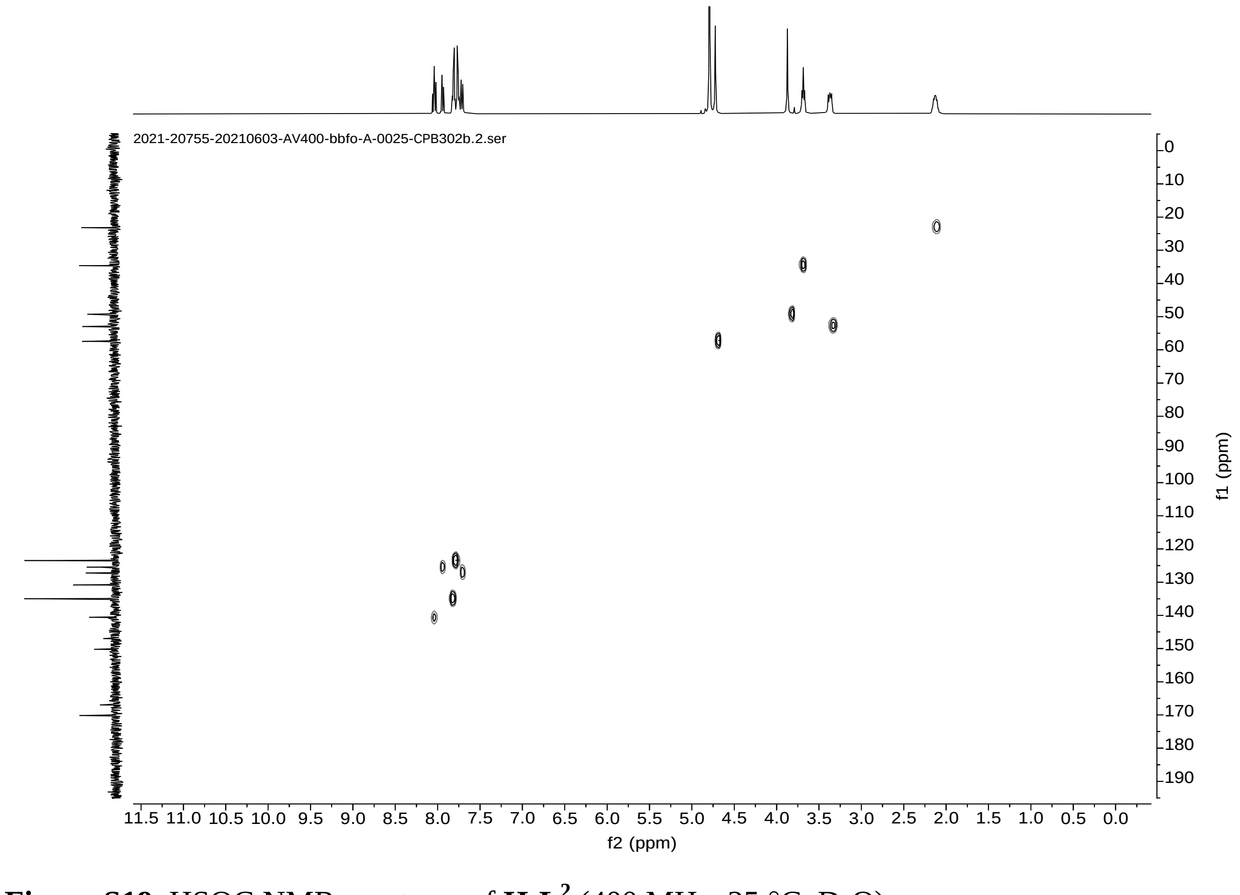

Figure S19. HSQC NMR spectrum of $\mathbf{H_2L^2}$ (400 MHz, 25 °C, D_2O).

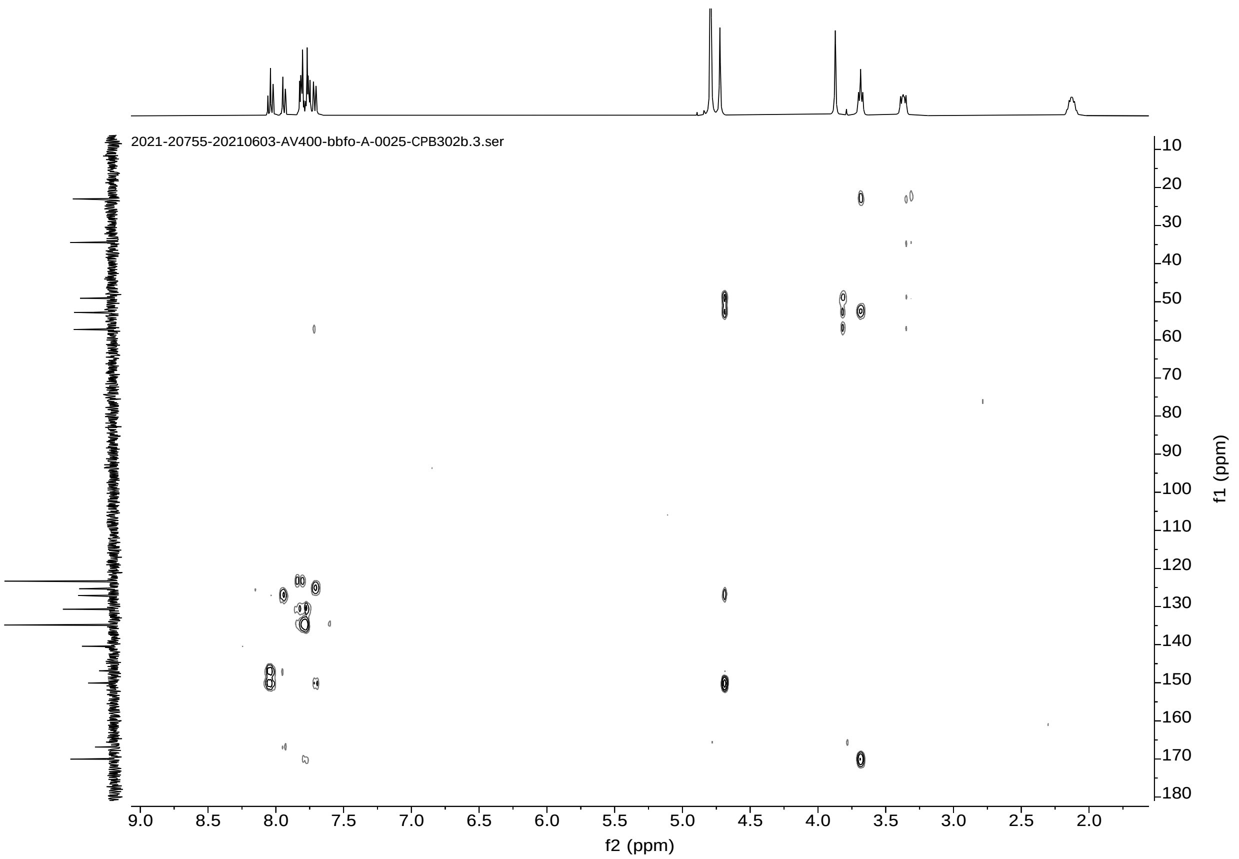

Figure S20. HMBC NMR spectrum of $\mathbf{H_2L^2}$ (400 MHz, 25 °C, D_2O).

NMR spectra of $(L^3)^{4-}$

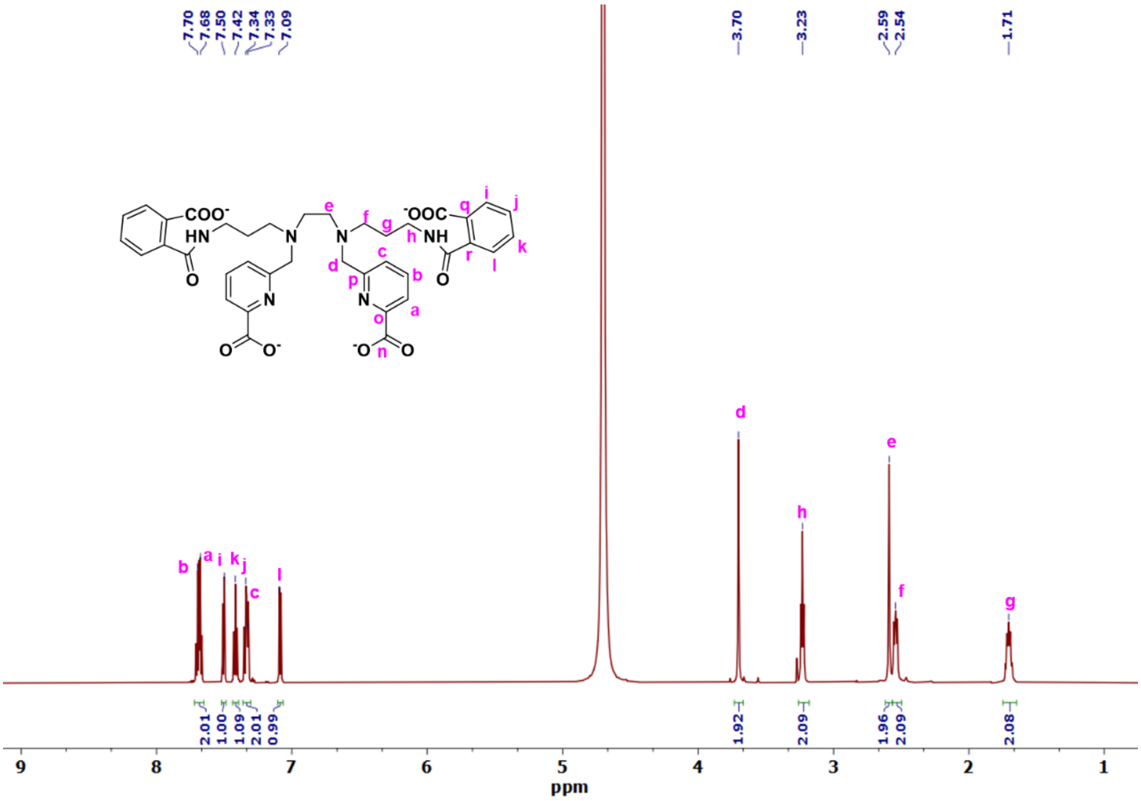

Figure S21. ^{1}H NMR spectrum of $(\mathbf{L^3})^{4-}$ (H_2L^2, pD = 12) (600 MHz, 25 °C, D_2O).

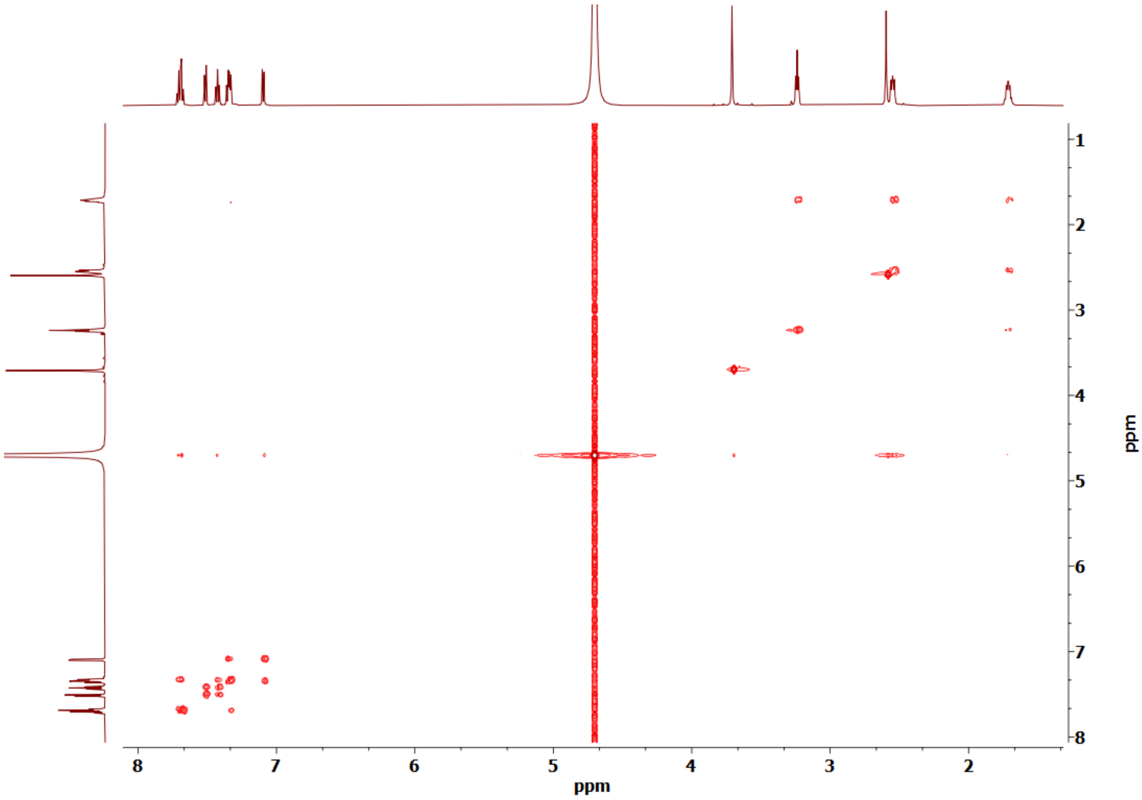

Figure S22. ^{1}H-^{1}H COSY NMR spectrum of $(\mathbf{L^3})^{4-}$ (H_2L^2, pD = 12) (600 MHz, 25 °C, D_2O).

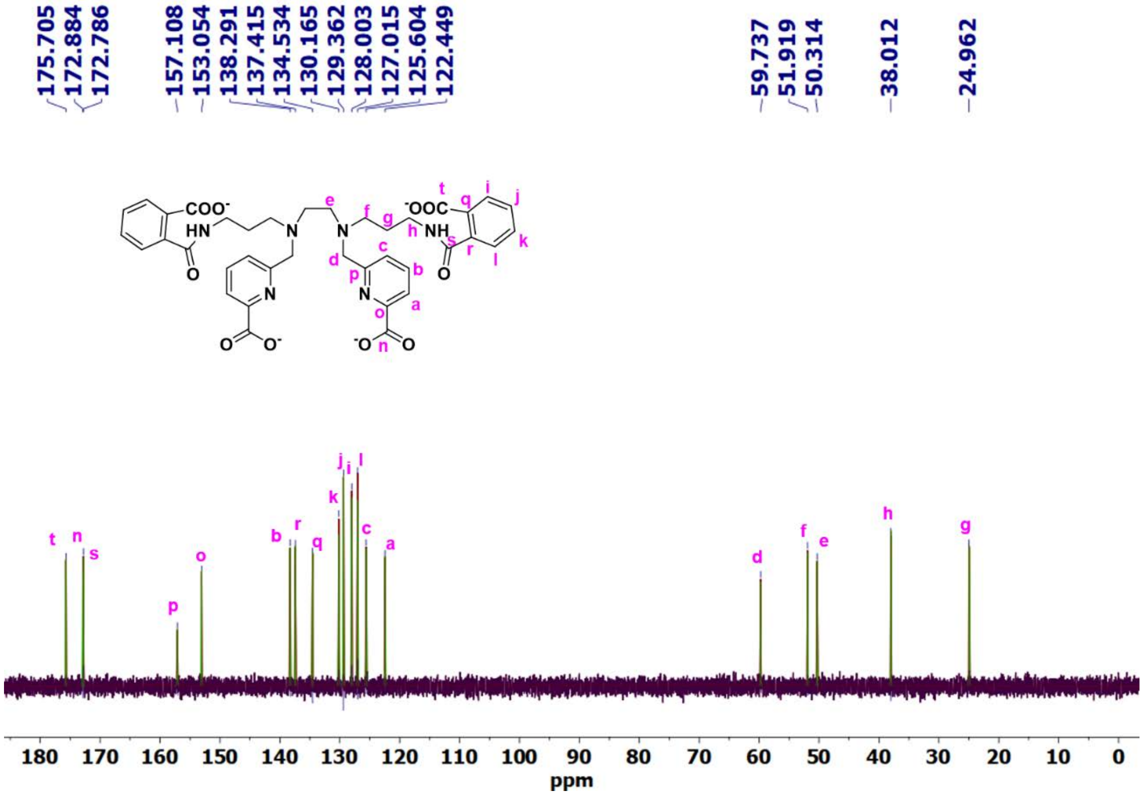

Figure S23. ^{13}C NMR spectrum of **$(L^3)^{4-}$** (H_2L^2, pD = 12) (600 MHz, 25 °C, D_2O).

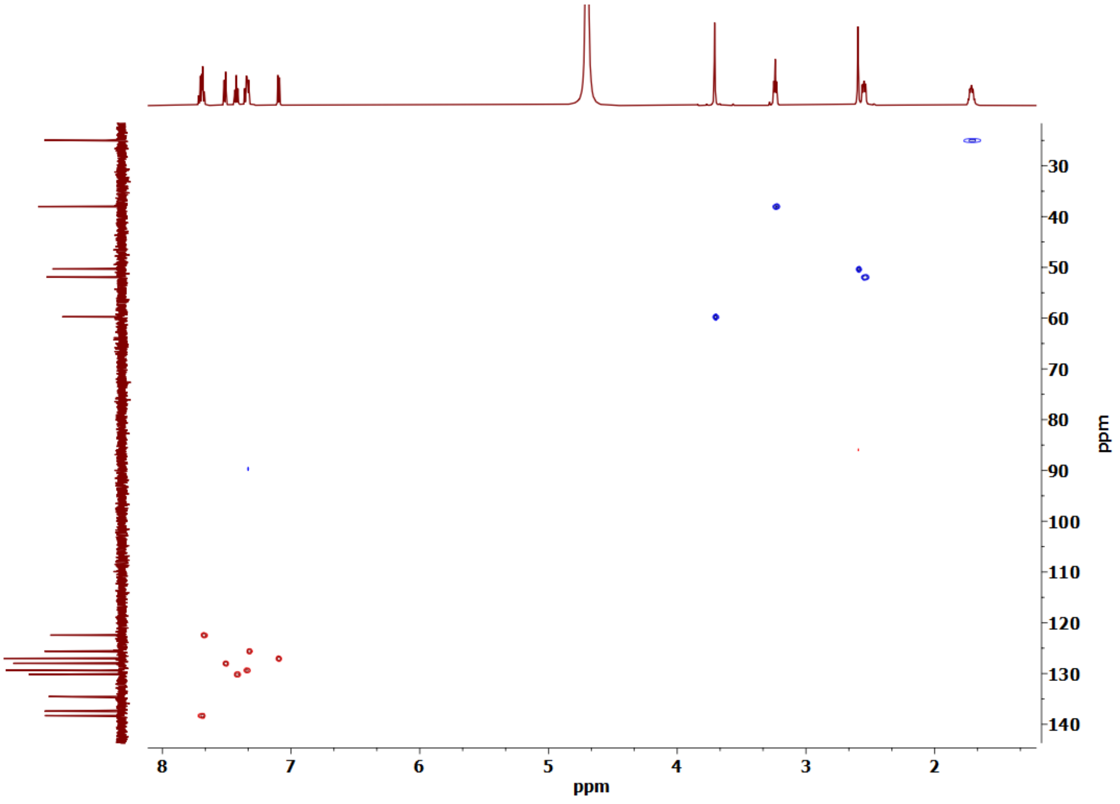

Figure S24. HSQC NMR spectrum of **$(L^3)^{4-}$** (H_2L^2, pD = 12) (600 MHz, 25 °C, D_2O).

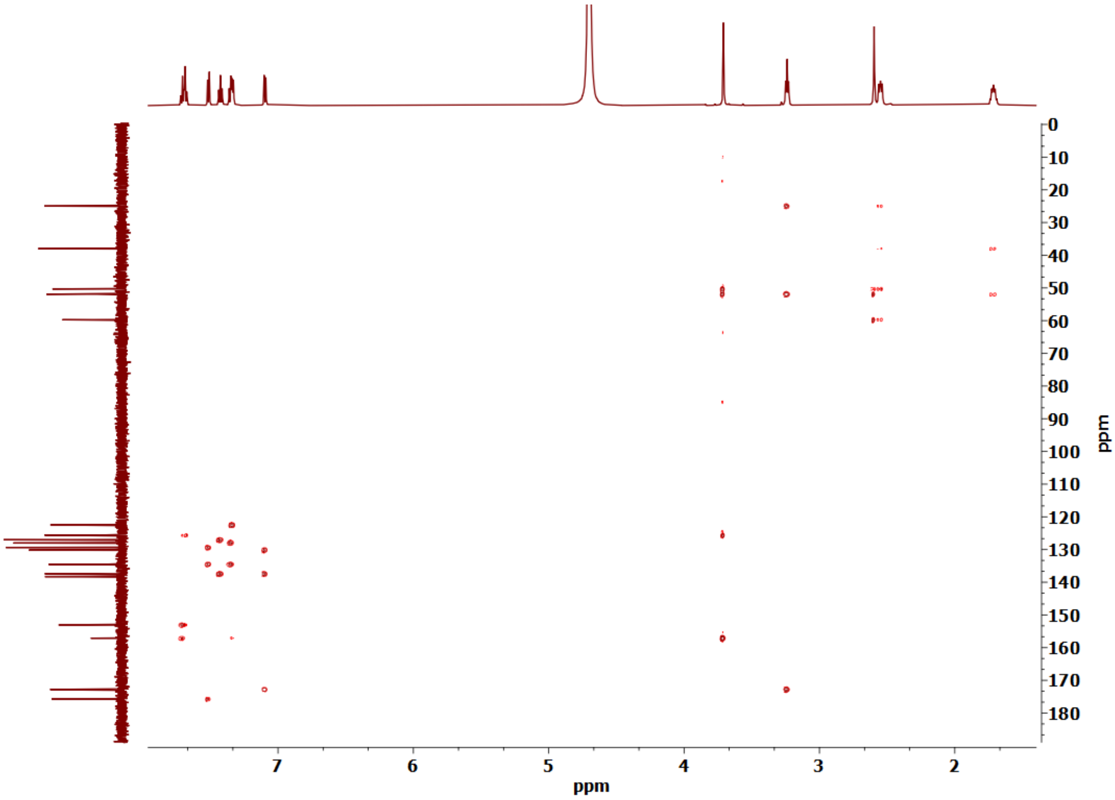

Figure S25. HMBC NMR spectrum of **$(L^3)^{4-}$** (H_2L^2, pD = 12) (600 MHz, 25 °C, D_2O).

NMR Gallium (III) complexation with H_2L^1

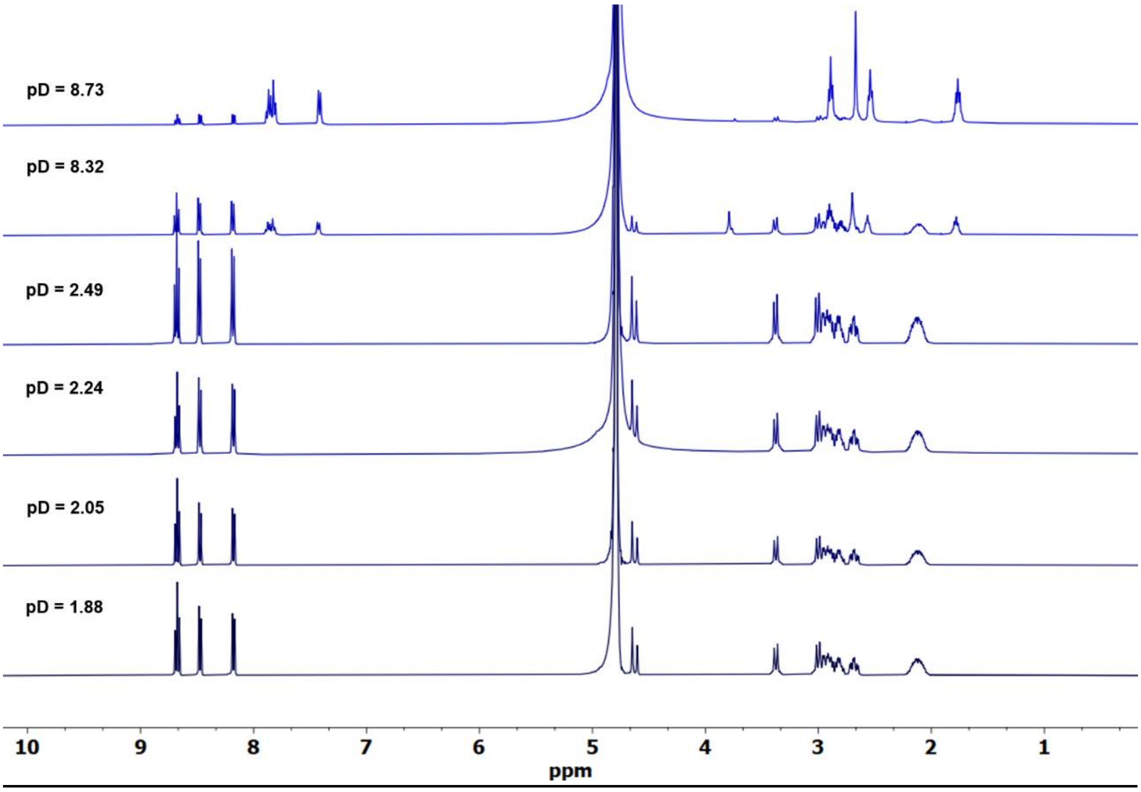

Figure S26. ^{1}H NMR spectra of the evolution of Ga^{3+} complexation with $\mathbf{H_2L^1}$ ligand at various pD values. $\mathbf{[Ga(H_2L^1)]^{3+}}$ (pD = 1.88, 2.05, 2.24, 2.49), and $\mathbf{H_2L^1}$ + $\mathbf{[GaL^1]^+}$ (pD = 8.32 and 8.73) (400 MHz, 25 °C, D_2O).

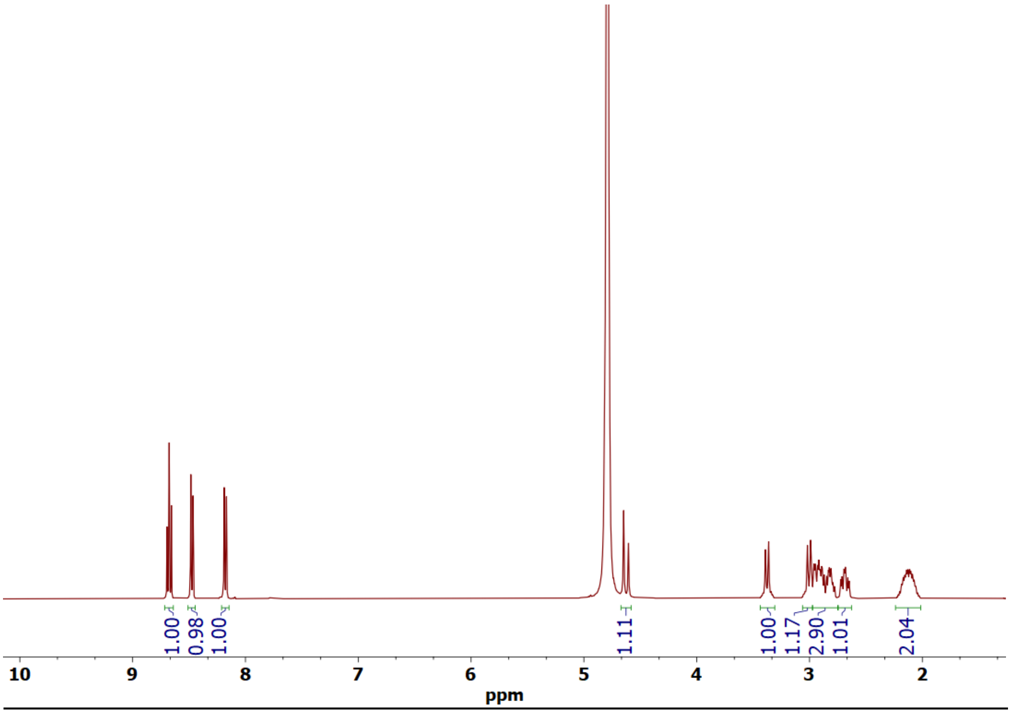

Figure S27. ^{1}H NMR spectrum of $\mathbf{[Ga(H_2L^1)]^{3+}}$ (pD = 2.1) (400 MHz, 25 °C, D_2O).

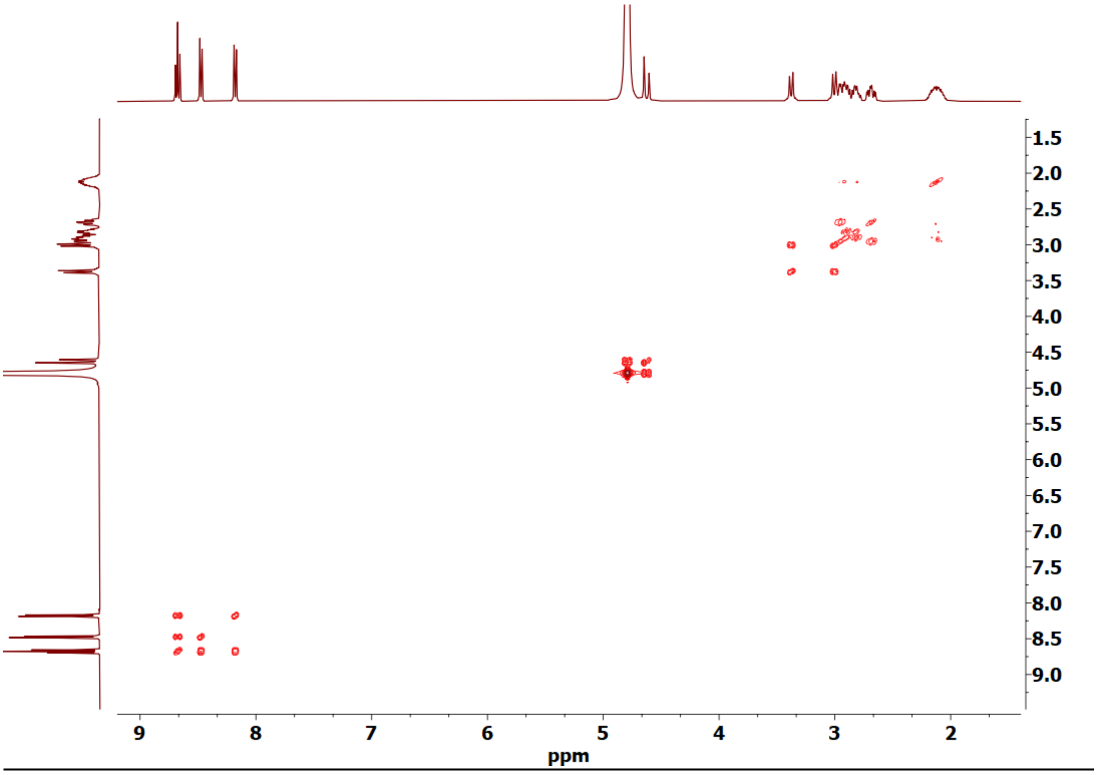

Figure S28. ^{1}H-^{1}H COSY NMR spectrum of $\mathbf{[Ga(H_2L^1)]^{3+}}$ (pD = 2.1) (400 MHz, 25 °C, D_2O).

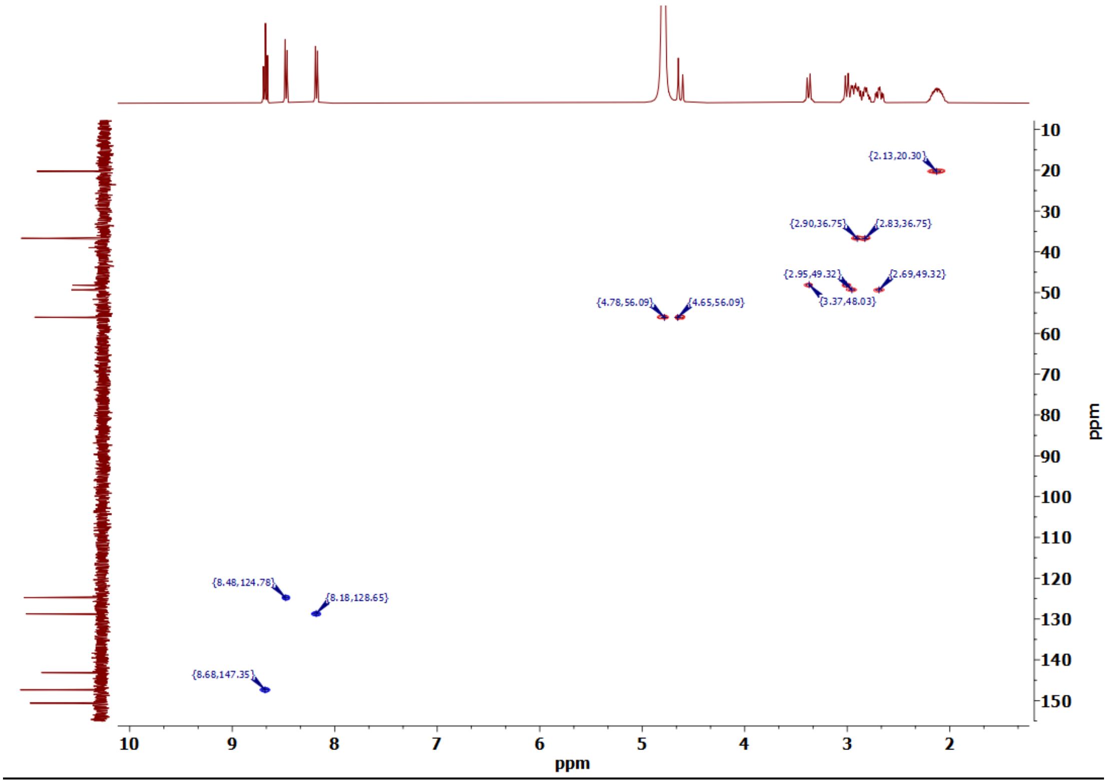

Figure S29. HSQC NMR spectrum of $\mathbf{[Ga(H_2L^1)]^{3+}}$ (pD = 2.1) (400 MHz, 25 °C, D_2O).

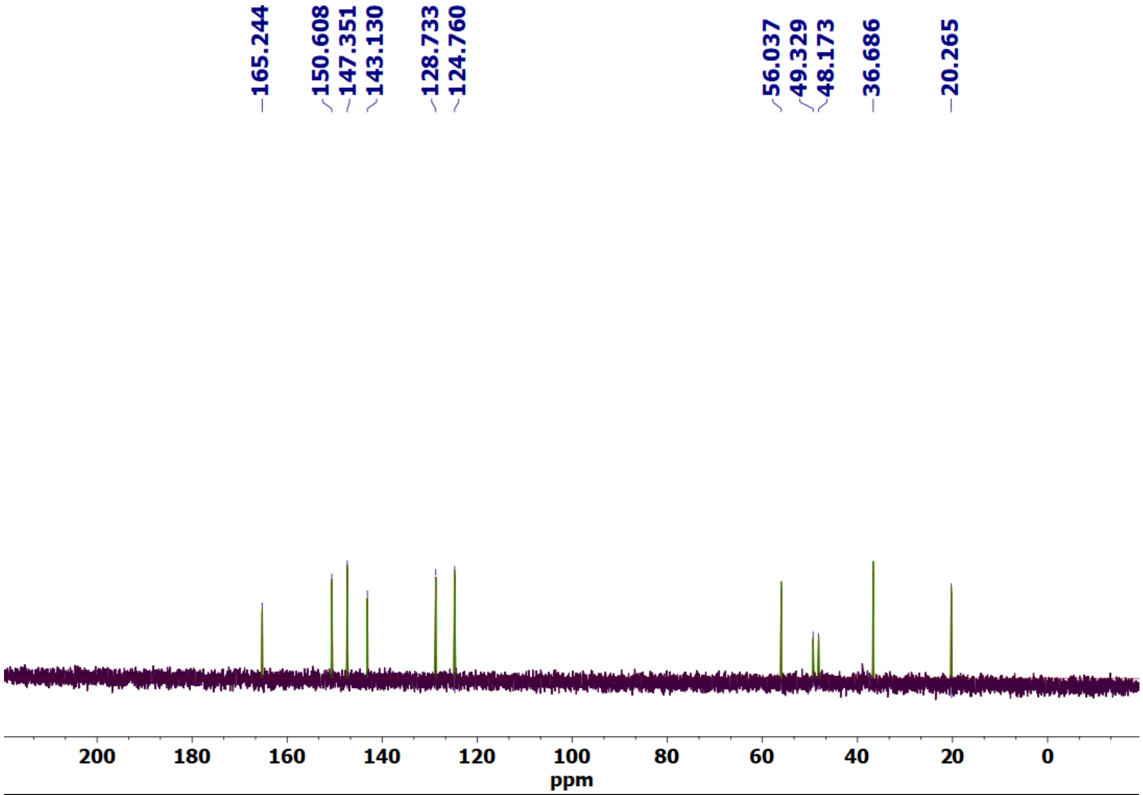

Figure S30. ^{13}C NMR spectrum of **$[Ga(H_2L^1)]^{3+}$** (pD = 2.1) (400 MHz, 25 °C, D_2O).

NMR Gallium (III) complexation with H_2L^2

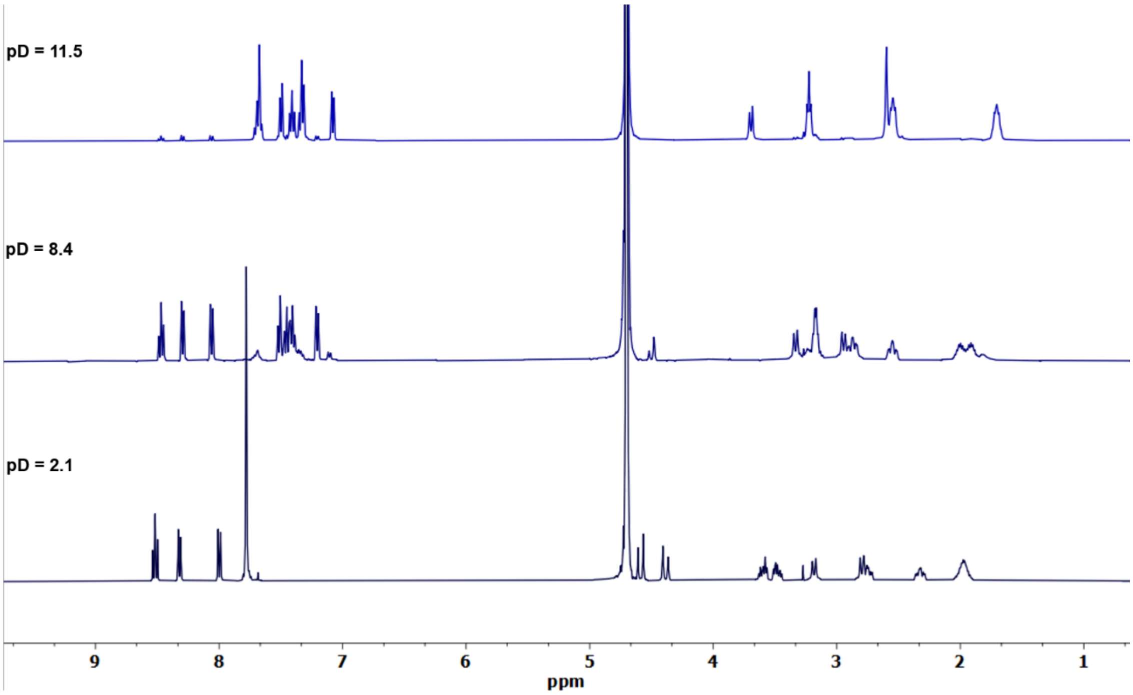

Figure S31. ^{1}H NMR spectra of the evolution of Ga^{3+} complexation with **H_2L^2** ligand at various pD values. **$[GaL^2]^+$** (pD = 2.1), **$[GaL^3]^-$** (pD = 8.4) and **$(L^3)^{4-}$ + $[GaL^3]^-$** (pD = 11.5) (400 MHz, 25 °C, D_2O).

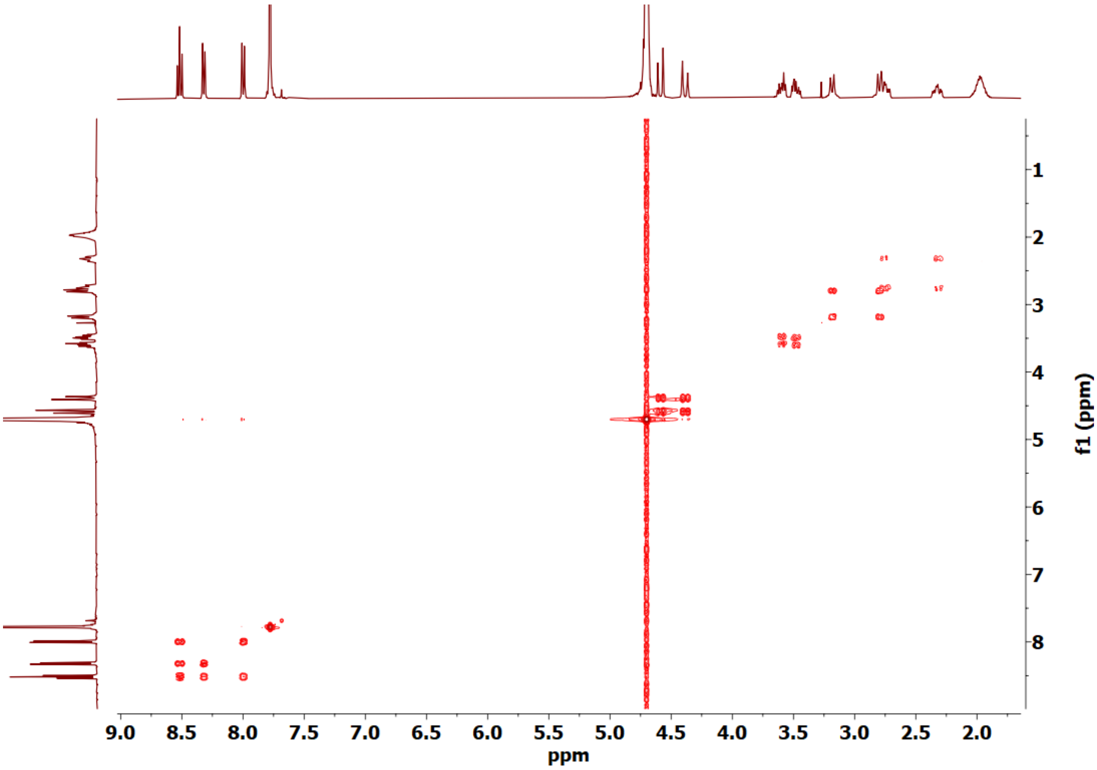

Figure S32. ^{1}H-^{1}H COSY NMR spectrum of **$[GaL^2]^+$** (400 MHz, 25 °C, D_2O, pD = 2.1).

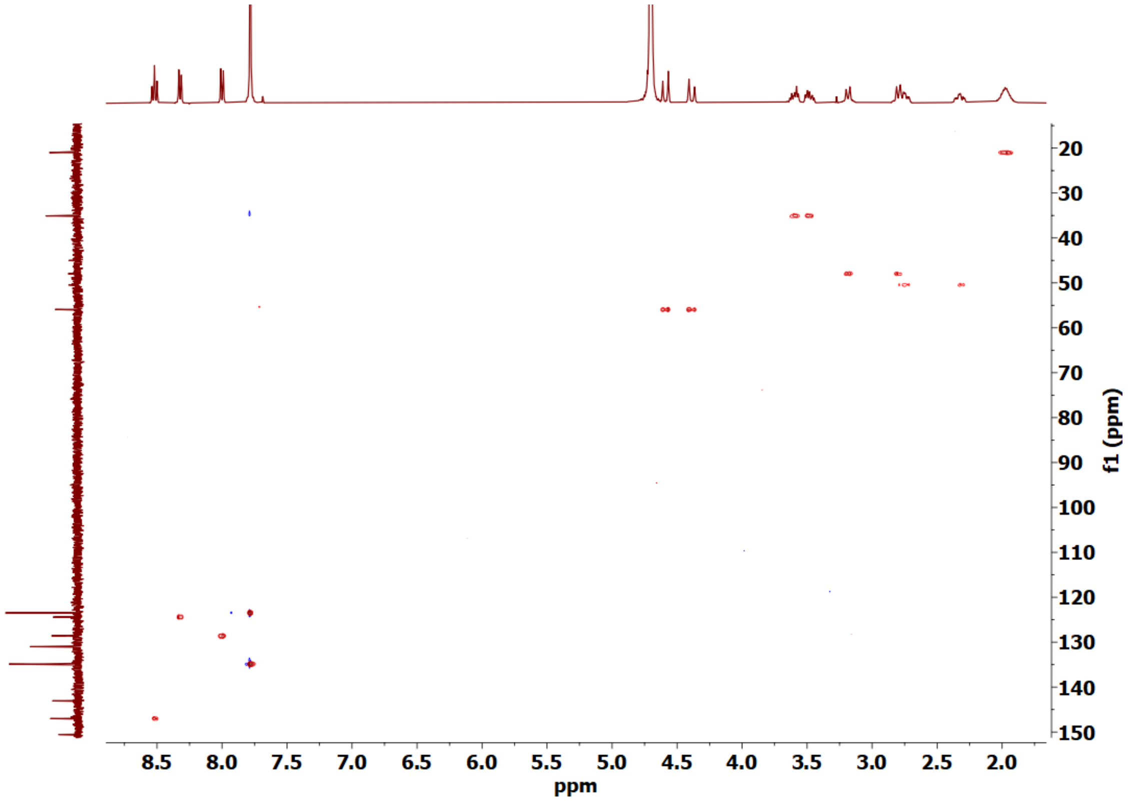

Figure S33. HSQC NMR spectrum of **[GaL²]⁺** (400 MHz, 25 °C, D_2O, pD = 2.1).

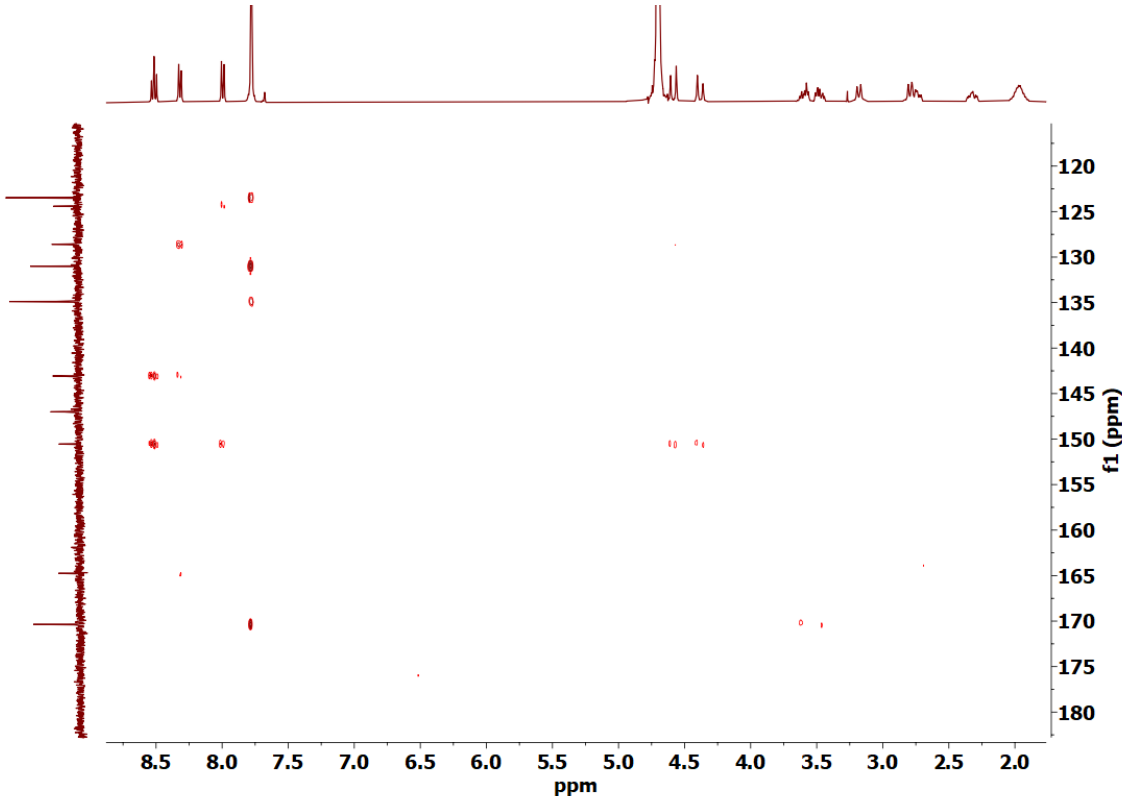

Figure S34. HMBC NMR spectrum of **[GaL²]⁺** (400 MHz, 25 °C, D_2O, pD = 2.1).

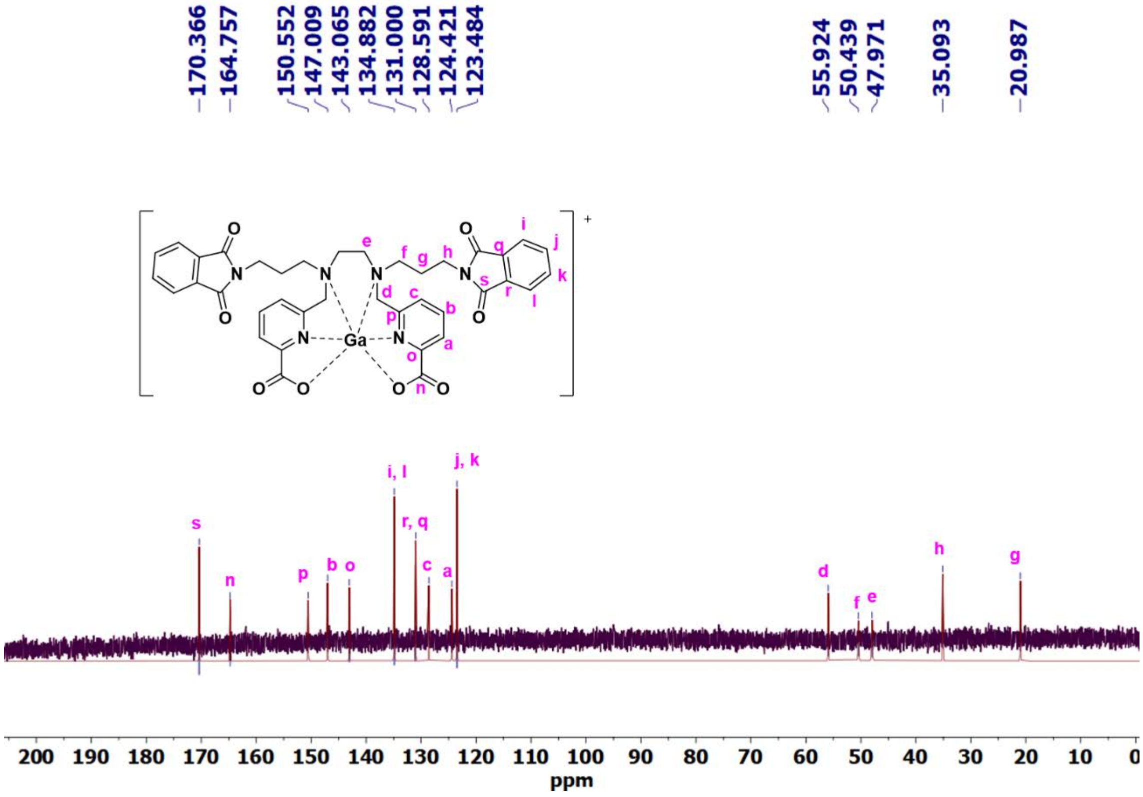

Figure S35. ^{13}C NMR spectrum of **[GaL^2]$^+$** (400 MHz, 25 °C, D_2O, pD = 2.1).

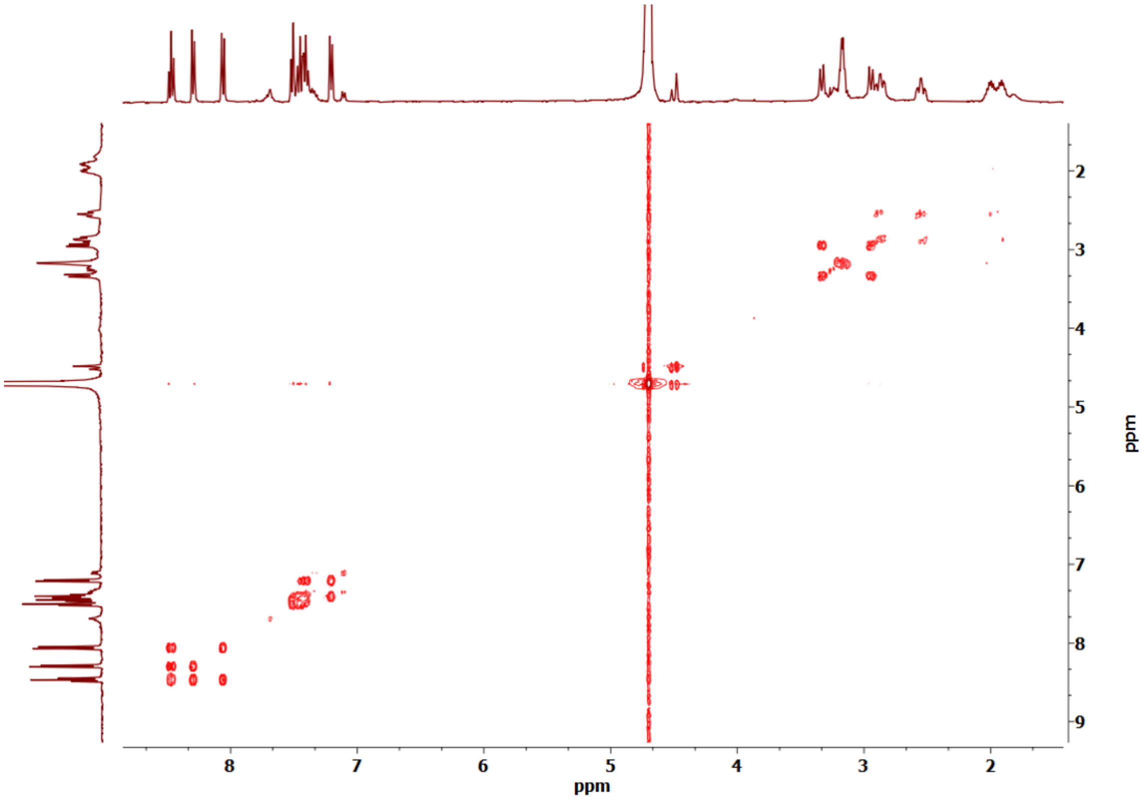

Figure S36. ^{1}H-^{1}H COSY NMR spectrum of **[GaL^3]$^-$** (400 MHz, 25 °C, D_2O, pD = 8.4).

Mass Spectrometry of Compounds 3, 5, H_2L^1 and H_2L^2.

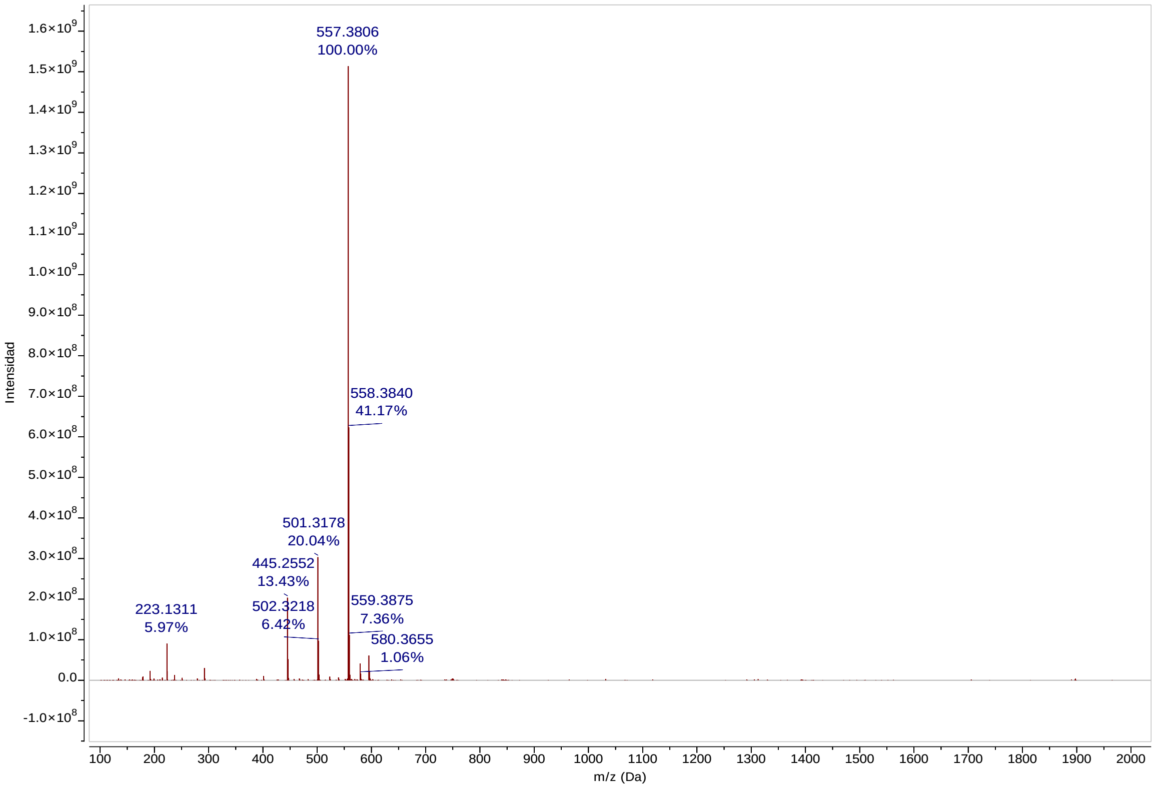

Figure S37. HR-ESI-MS of **compound 3**

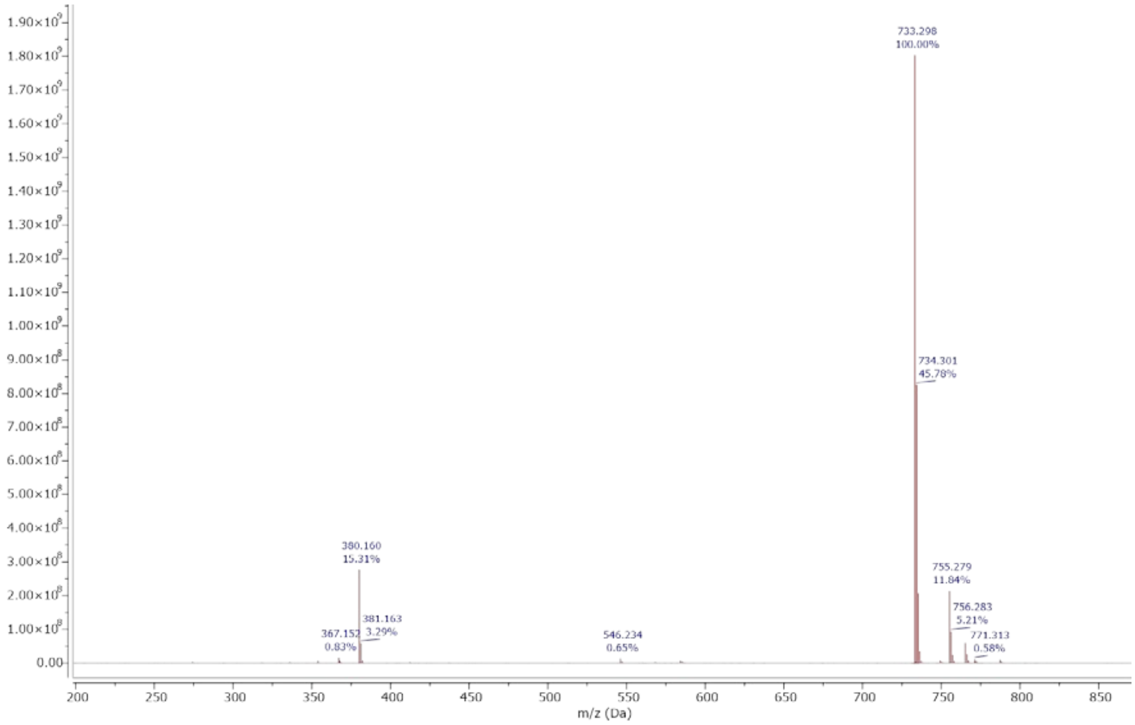

Figure S38. HR-ESI-MS of **compound 5**

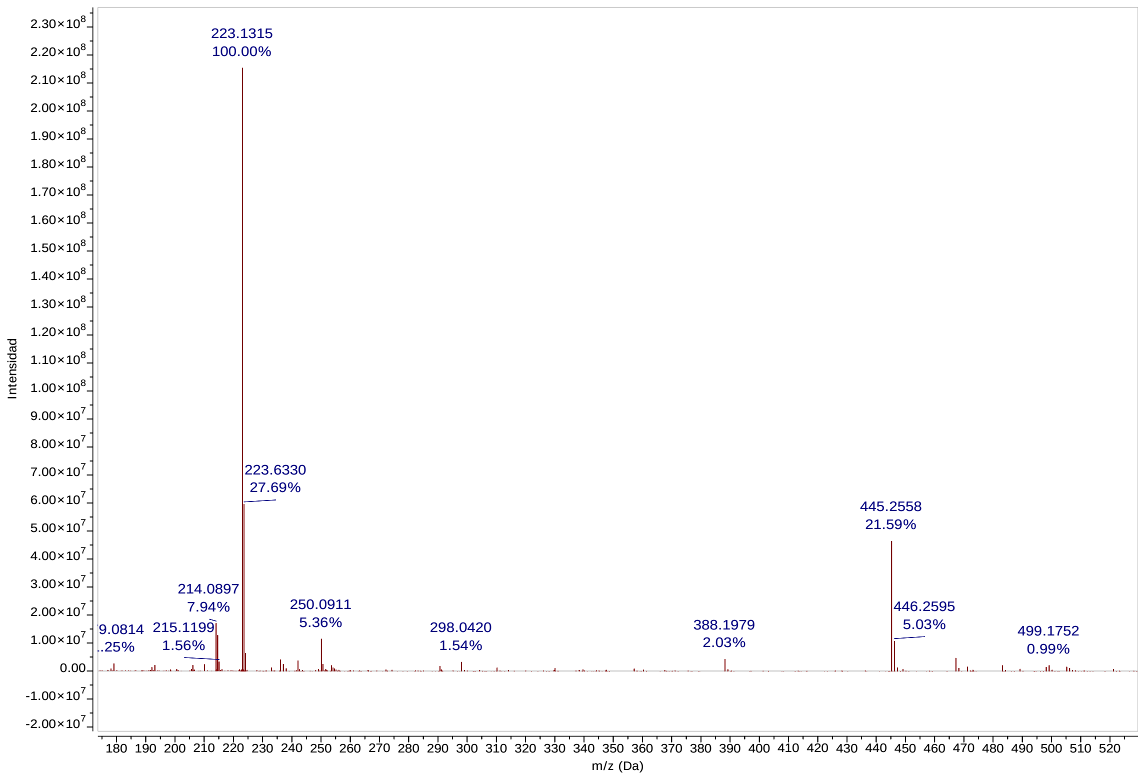

Figure S39. HR-ESI-MS of **H_2L^1** $[C_{22}H_{32}N_6O_4+2H]^{+2}$ calcd.: 223.1315; found: 223.1315

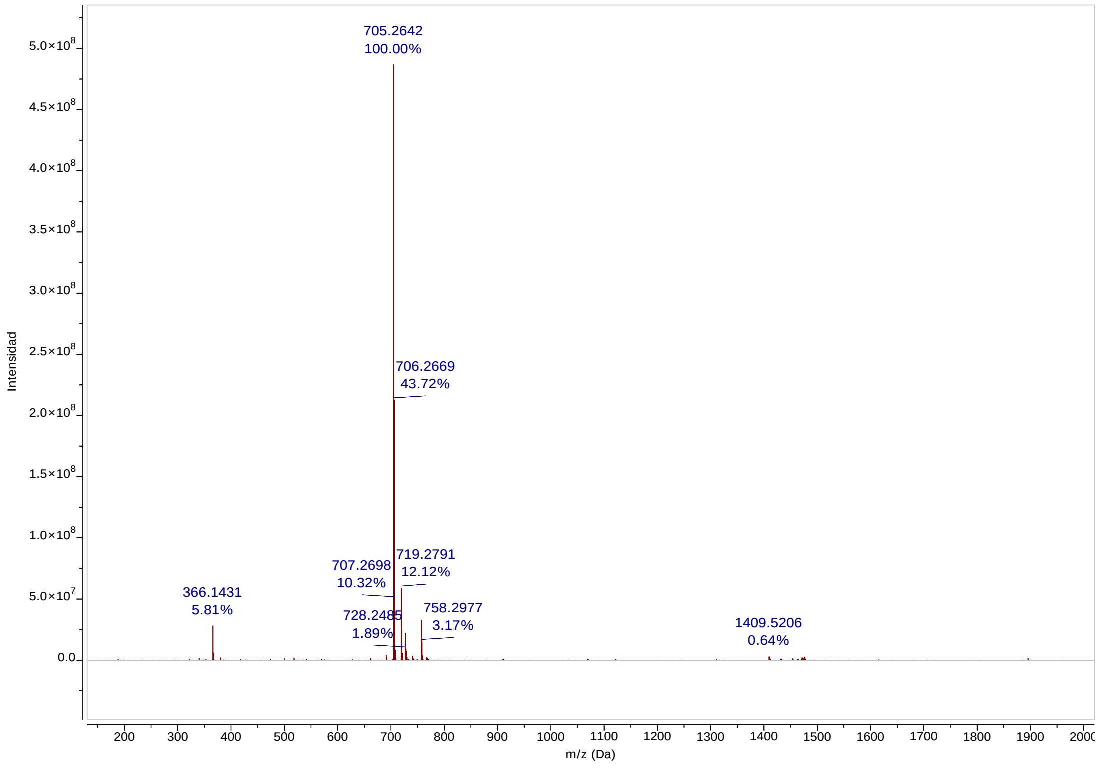

Figure S40. HR-ESI-MS of **H_2L^2**

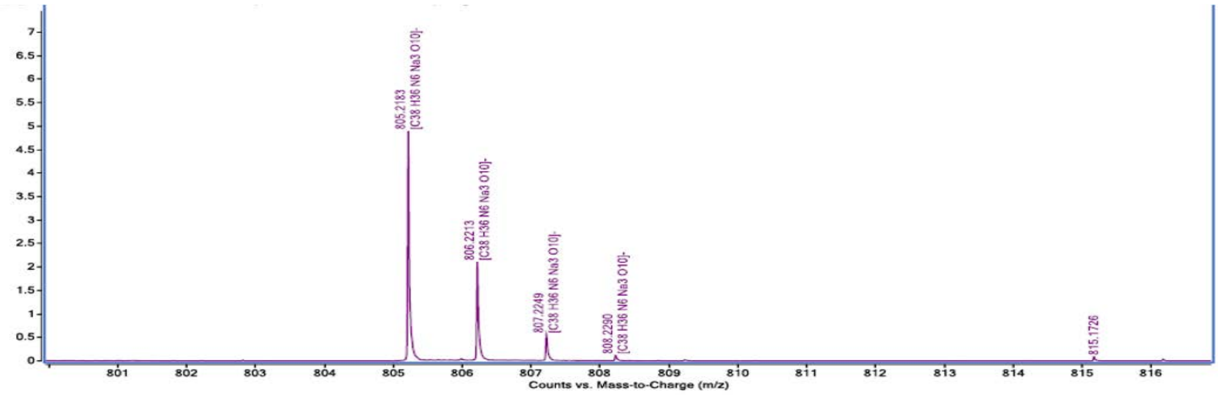

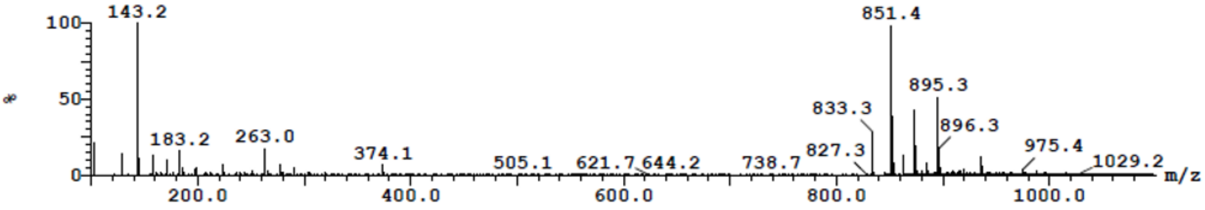

Figure S41. Mass spectra of $\mathbf{H_2L^2}$ at **pH = 12**. Top: HR-ESI-MS (ESI^-); bottom: ESI-MS (ESI^+). [At this pH, the phthalimide groups are hydrolysed and the only species present is $\mathbf{(L^3)^{4-}}$].

Solution Thermodynamics

Protonation Constants of H_2L^1 and H_2L^2

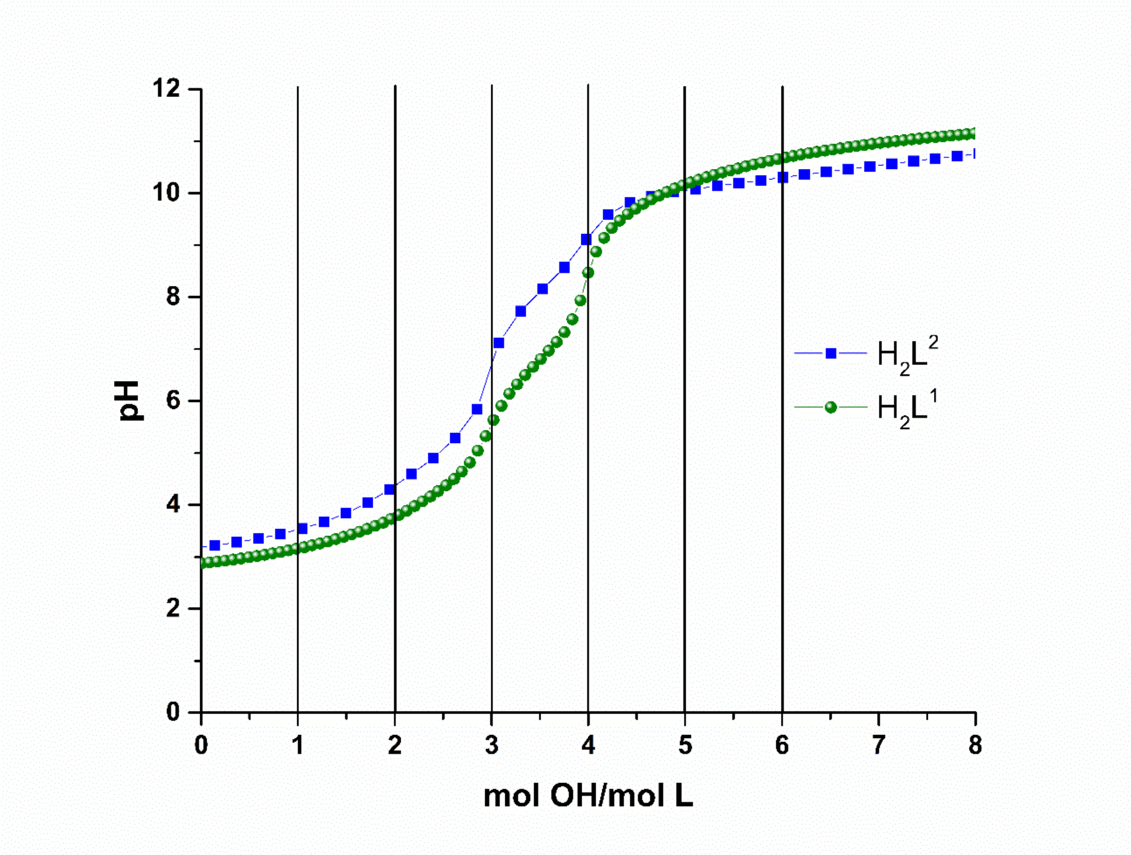

Figure S42. Titration curves of acidic solutions of H_2L^1 and H_2L^2 ligands. $[H_2L^1]$ = 9.56×10^{-4} M, $[H_2L^2] = 5.22 \times 10^{-4}$ M at 25 °C and $I = 0.16$ M NaCl.

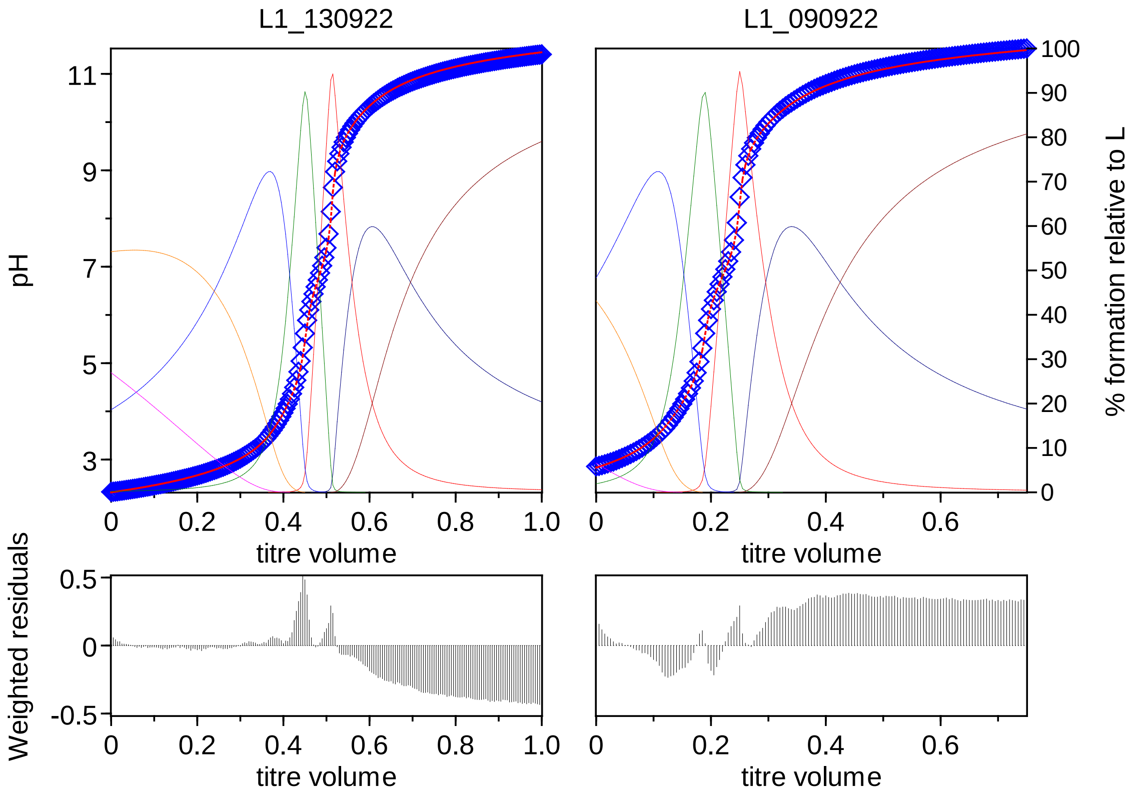

Figure S43. Potentiometric titration plots of aqueous solutions containing H_2L^1, $[H_2L^1]$ = 9.56×10^{-4} M, at 25 °C and I = 0.16 M (NaCl).

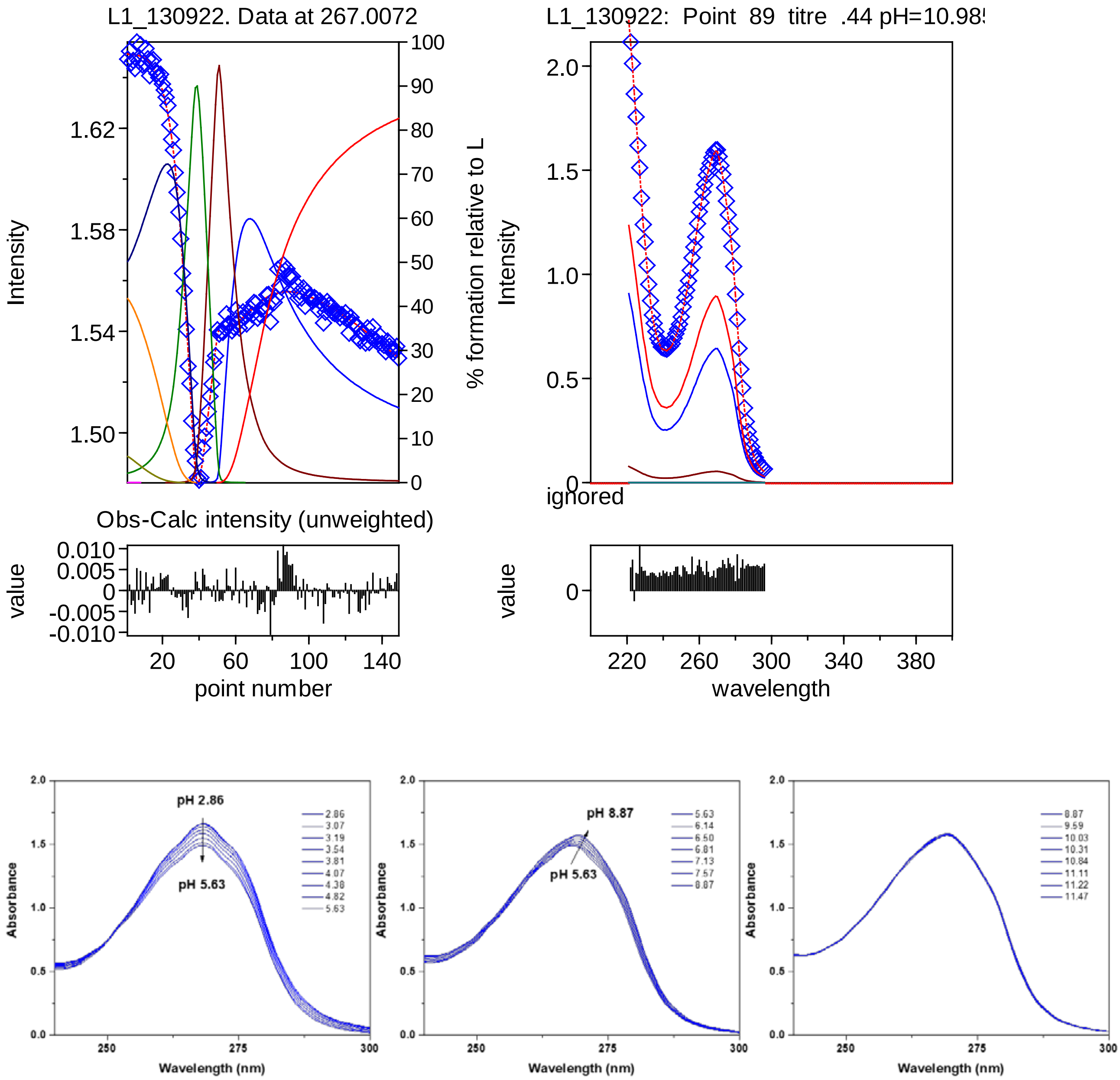

Figure S44. (Top) Change of absorbance (267 nm) during UV-potentiometric titration of $[H_2L^1]$ = 9.56 × 10^{-4} M, 25 °C, l = 0.2 cm, I = 0.16 M NaCl (left); blue dots are experimental data points and red dotted line represents the fit versus data points. Obtained (blue) and fitted (red) spectra for pH = 10.98 (right). (Bottom) Representative spectra of the UV-potentiometric titration.

^{1}H NMR titration of H_2L^1

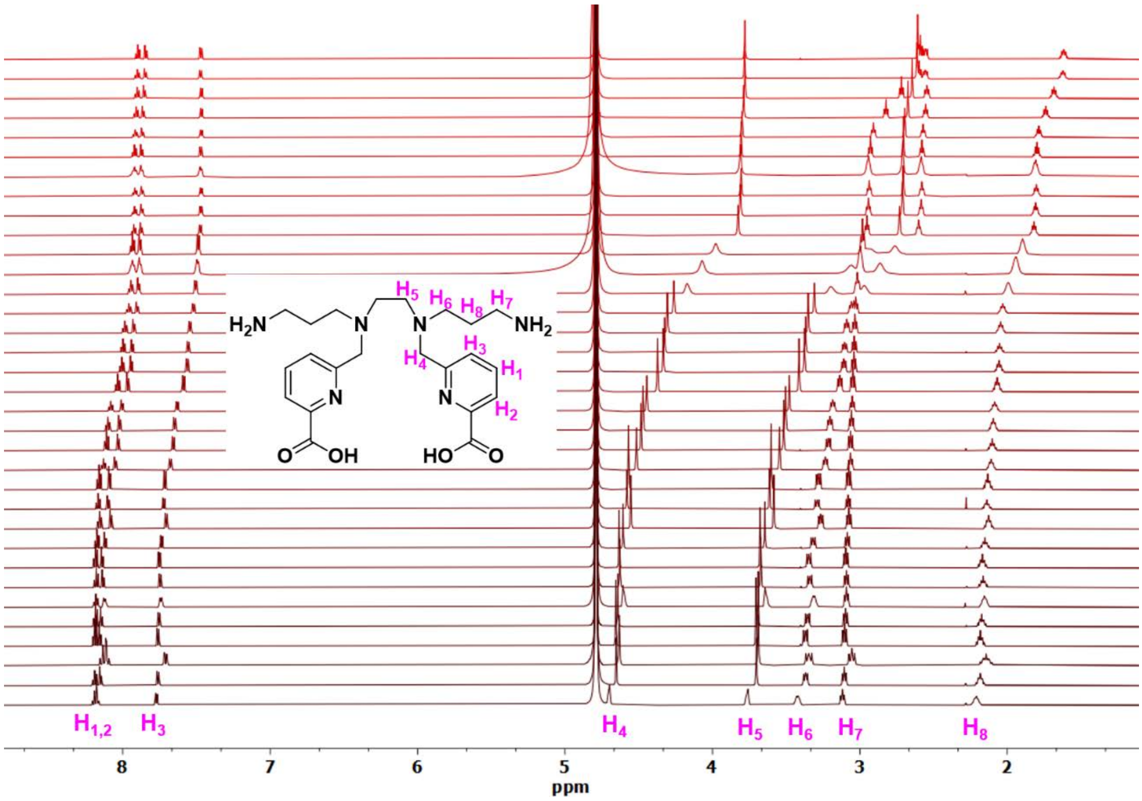

Figure S45. Progression of the ^{1}H NMR titration of H_2L^1 (pD 1.77 (bottom) to pD 12.3 (top)), (600 MHz, 25 °C, D_2O). For consistency, note that: H_1 = Hb; H_2 = Ha; H_3 = Hc; H_4 = Hd; H_5 = He; H_6 = Hf; H_7 = Hh; H_8 = Hg.

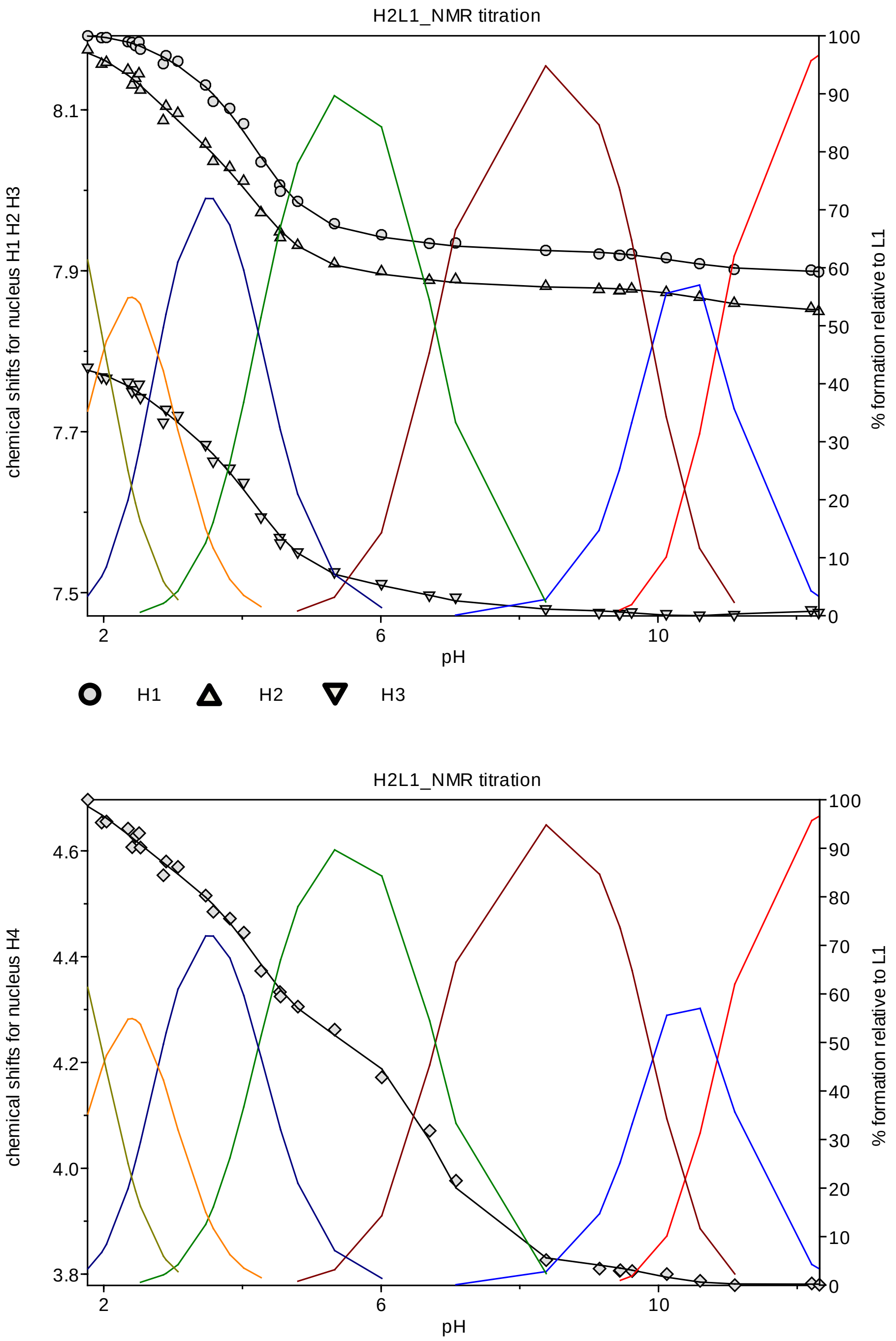

Figure S46. Experimental data points for the ^{1}H NMR titration of H_2L^1 at different pD values (600 MHz, 25 °C, D_2O). The experimental data points follow the calculated line using the HypNMR software[1] with the protonation constants from potentiometric titrations in Table 3. For consistency, note that: H_1 = Hb; H_2 = Ha; H_3 = Hc; H_4 = Hd.

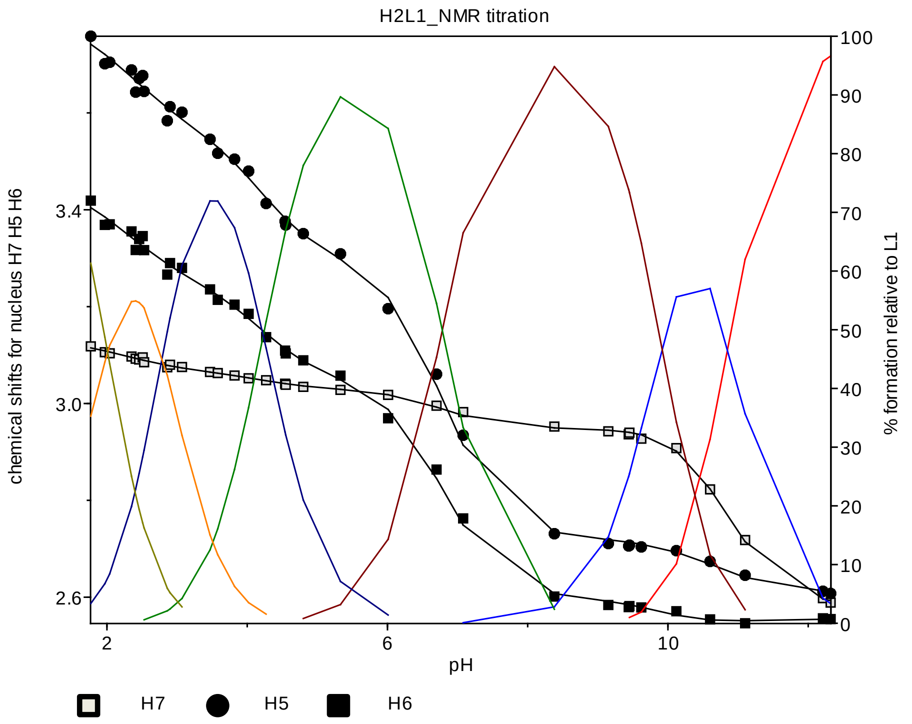

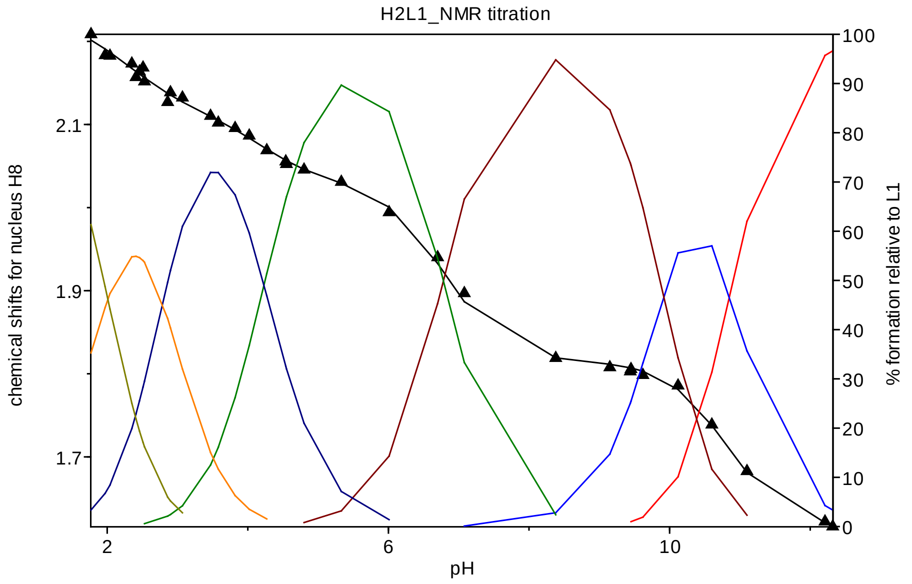

Figure S46 (continuation). Experimental data points for the ^{1}H NMR titration of H_2L^1 at different pD values (600 MHz, 25 °C, D_2O). The experimental data points follow the calculated line using the HypNMR software[1] with the protonation constants from potentiometric titrations in Table 3. For consistency, note that: H_5 = He; H_6 = Hf; H_7 = Hh; H_8 = Hg.

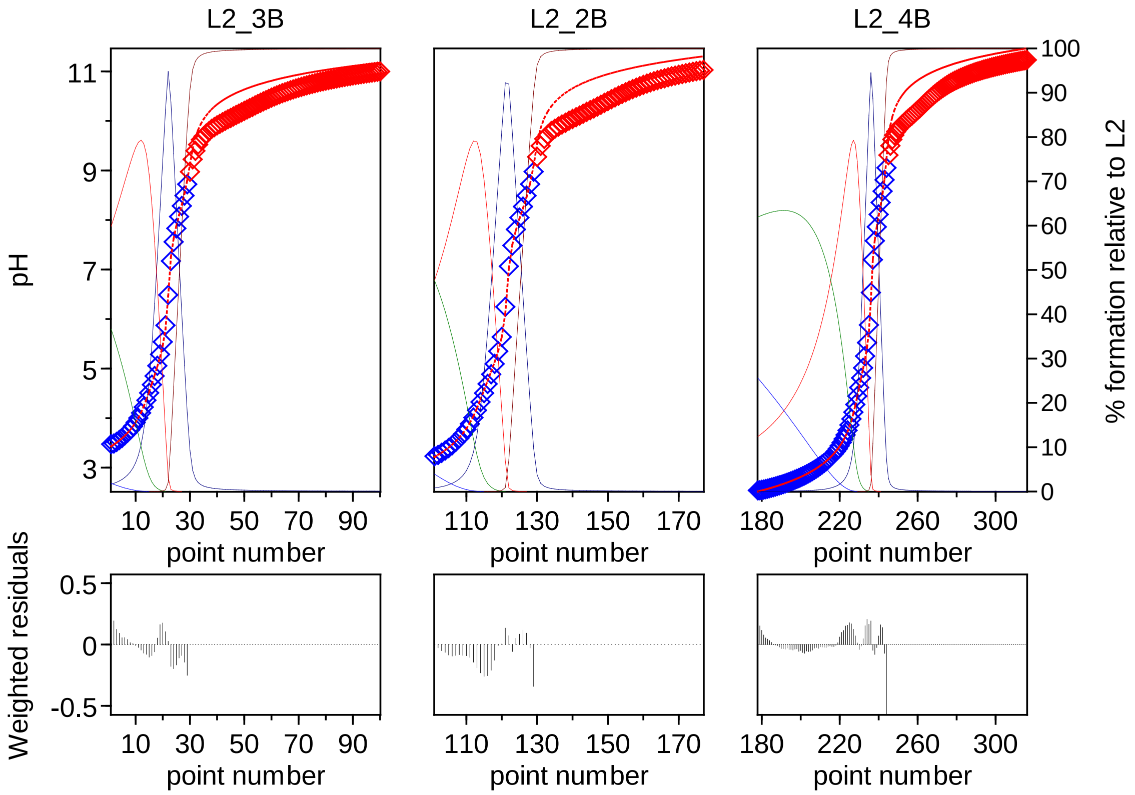

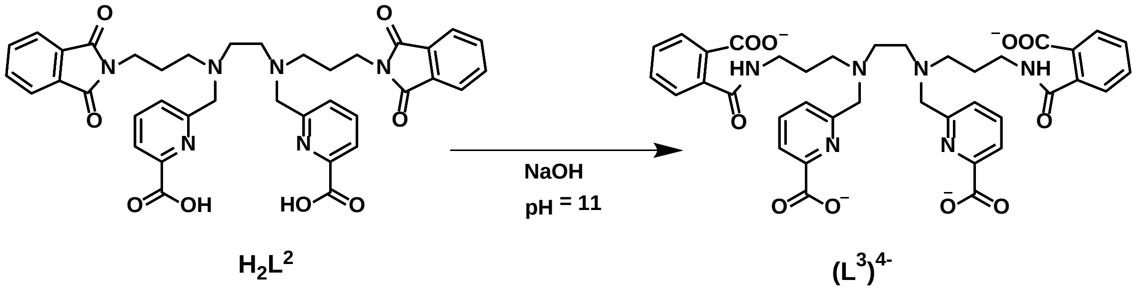

Figure S47. Potentiometric titration plots of aqueous solutions containing H_2L^2, $[H_2L^2]$ = 5.87 × 10^{-4} M, at 25 °C and I = 0.16 M (NaCl). Red experimental points were not included in the calculation of protonation constants as are related to the partial hydrolysis of the phthalimide groups in H_2L^2. Protonation constants are reported in Table 3.

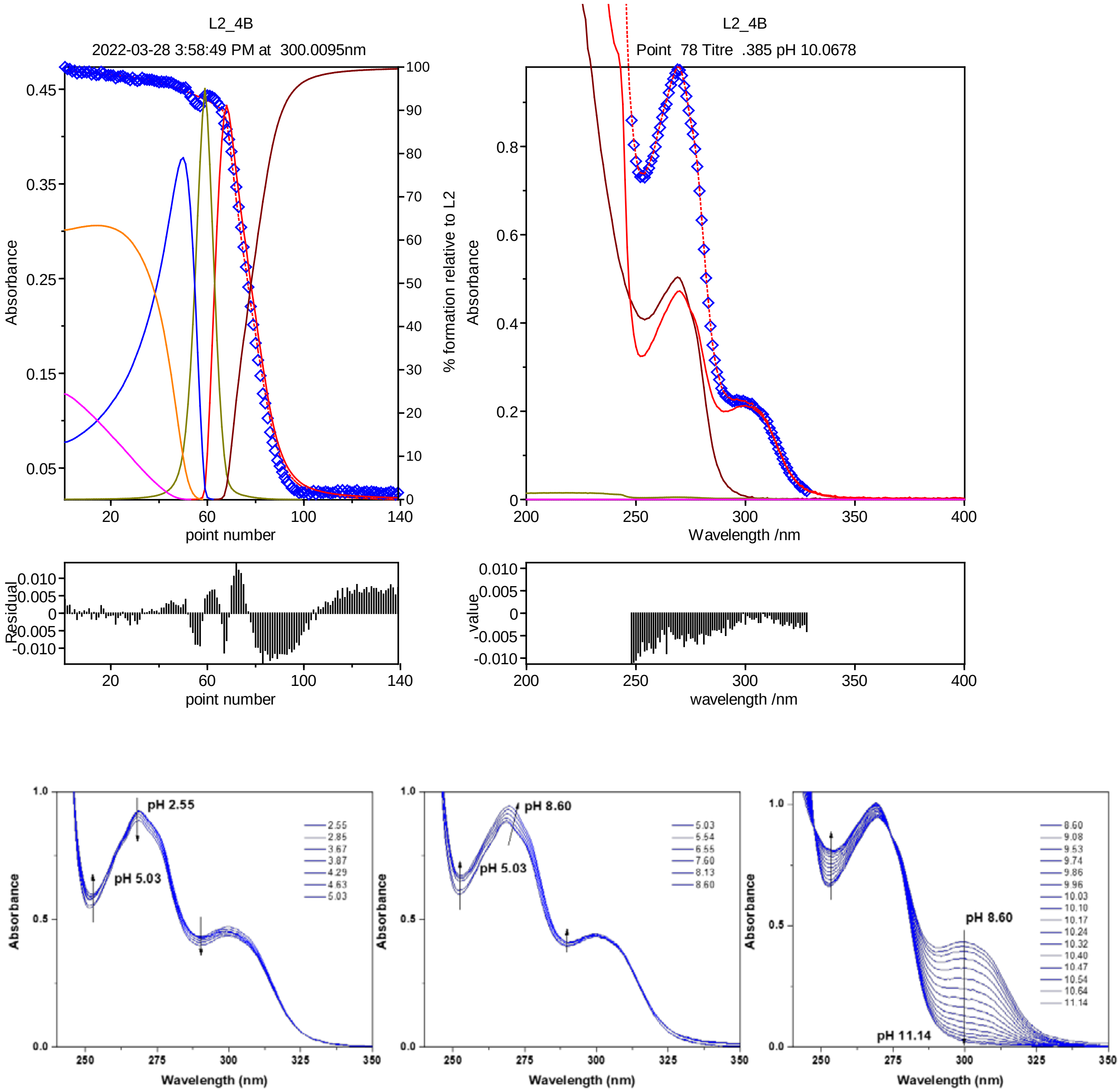

Figure S48. (Top) Change of absorbance (300 nm) during UV-potentiometric titration of $[H_2L^2]$ = 5.87 × 10^{-4} M, 25 °C, l = 0.2 cm, *I* = 0.16 M NaCl (left); blue dots are experimental data points and red dotted line represents the fit versus data points. The fitting included protonation constants in Table 3 and a value of 10 was given for the reaction of partial hydrolysis of phthalimide groups in H_2L^2 where two equivalents of base were consumed to form the product $(L^3)^{4-}$. Obtained (blue) and fitted (red) spectra for pH = 10.00 (right). (Bottom) Representative spectra of the UV-potentiometric titration.

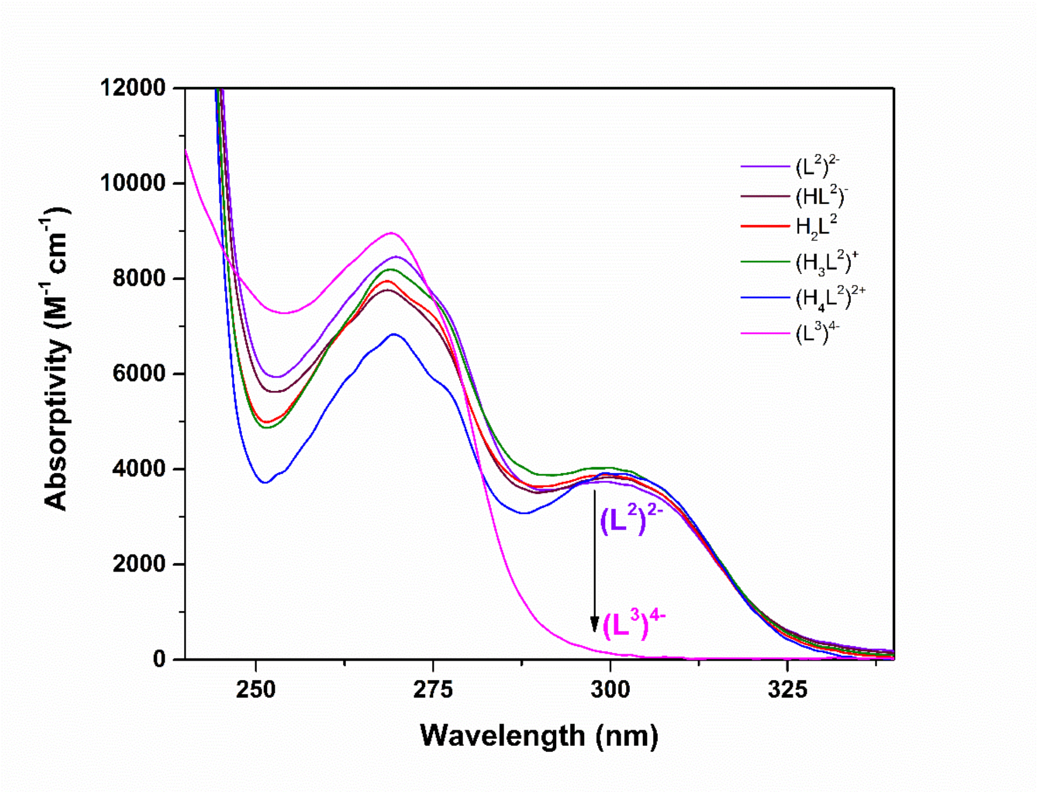

Figure S49. Electronic spectra of the fifth absorbing species of H_2L^2 and the hydrolysis product $(L^3)^{4-}$ (pink). The absorption band at 300 nm of the fully deprotonated species $(L^2)^{2-}$ disappears with the partial hydrolysis of the phthalimide functional groups at pH 10 giving the product $(L^3)^{4-}$ (pink).

Stability constants of H_2L^1 and H_2L^2 with Cu(II) and Ga(III)

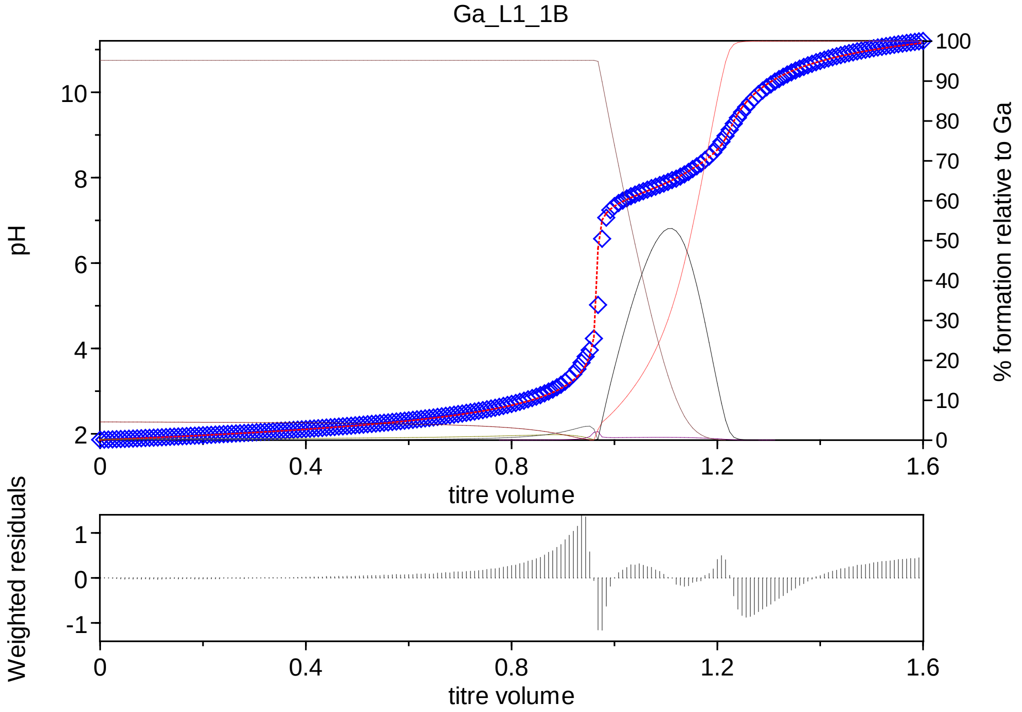

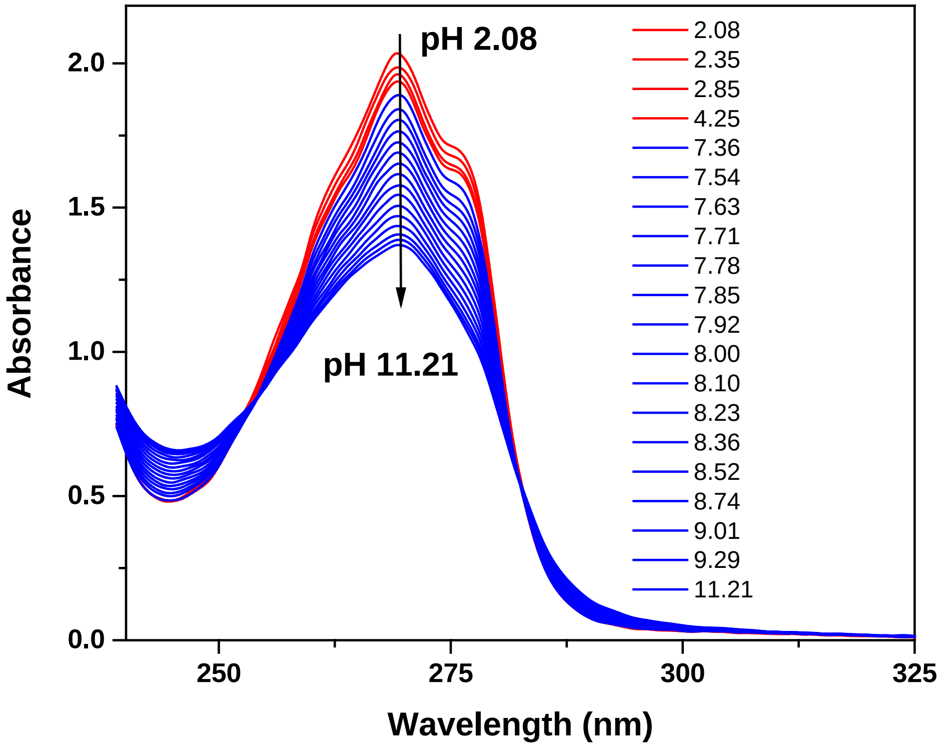

Figure S50. Potentiometric titration plots (top) of aqueous solutions containing H_2L^1 and Ga^{3+}; $[H_2L^1] = [Ga^{3+}] = 8.92 \times 10^{-4}$ M, at 25 °C and $I = 0.16$ M (NaCl). Blue dots are experimental data points and red dotted line is the fitting using stability constants in Table 3 and Table S3. Selected UV spectra (bottom) of the Ga(III)-H_2L^1 potentiometric titration showing the decomplexation of the $[Ga(L^1)]^+$ species as the $[Ga(OH)_4]^-$ complex is formed (l = 0.2 cm).

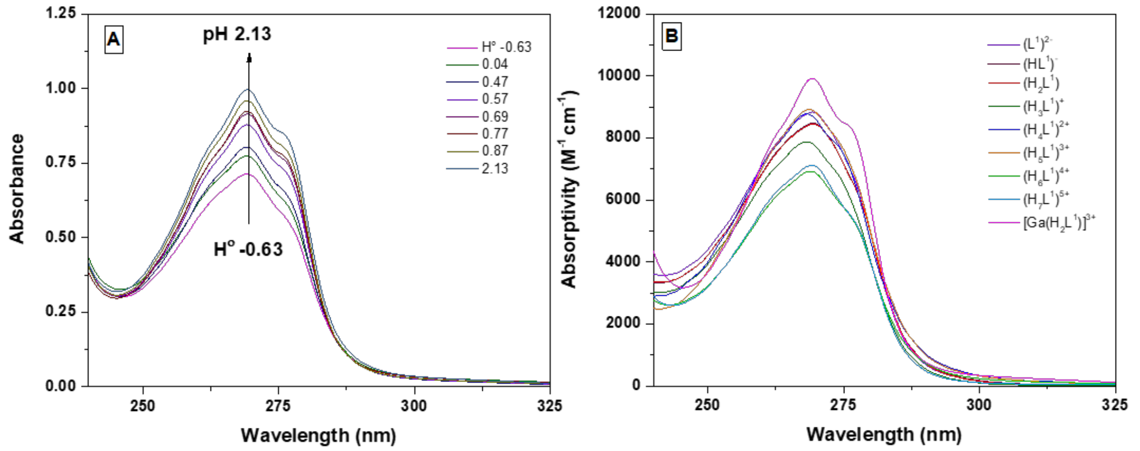

Figure S51. A) Spectra of the Ga^{3+}/H_2L^1 UV-acidic batch titration; [Ga^{3+}] = [H_2L^1] = 1 × 10^{-4} M, at 25 °C, l = 1 cm and *I* = 0.16 M (NaCl) (when possible). B) Molar absorptivities of H_2L^1 ligand and the $[Ga(H_2L^1)]^{3+}$ metal complex.

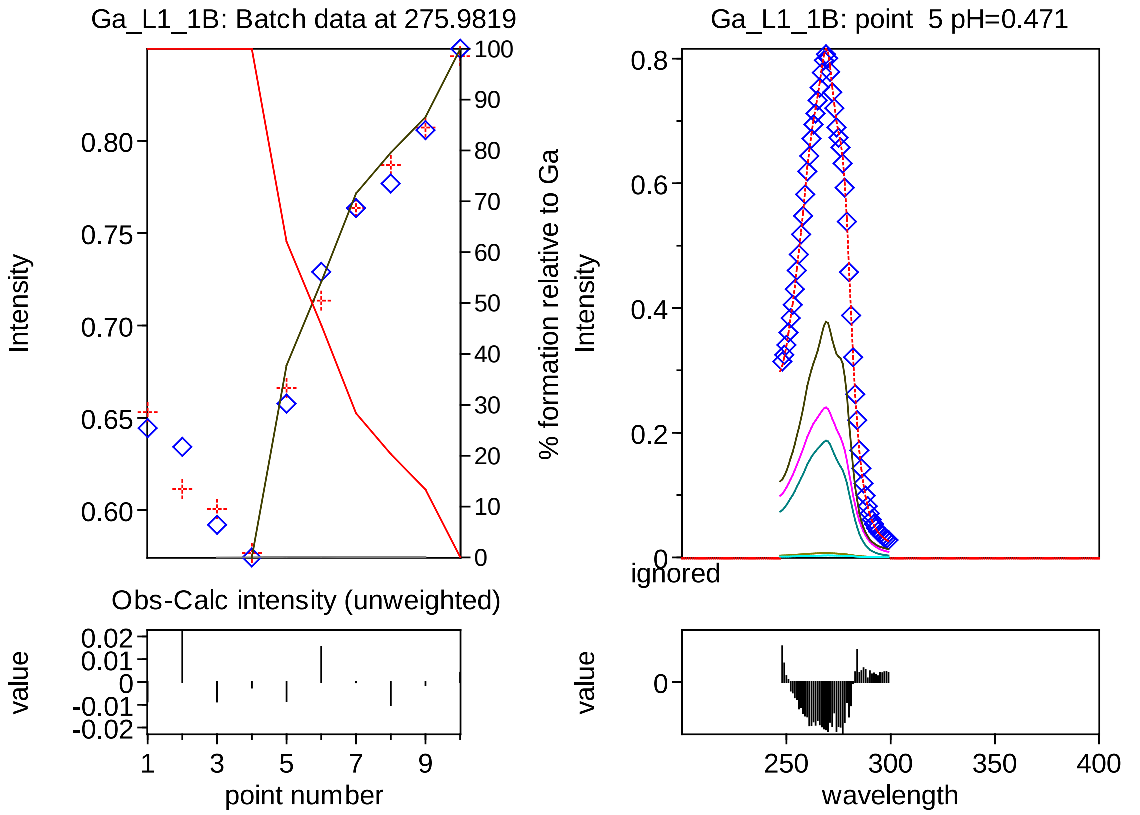

Figure S52. Fitting of the UV-acidic batch experiments (λ = 276 nm) of the Ga^{3+}-H_2L^1 system; [Ga^{3+}] = [H_2L^1] = 1 × 10^{-4} M at various pH values at 25 °C and *I* = 0.16 M NaCl (when possible) (left). Fitted spectrum of the Ga^{3+}- H_2L^1 UV-acidic sample (pH = 0.47) considering the molar absorptivities of the free ligand (H_2L^1) (right). The graph on the left contains the calculated speciation plot as a function of gallium(III) concentration.

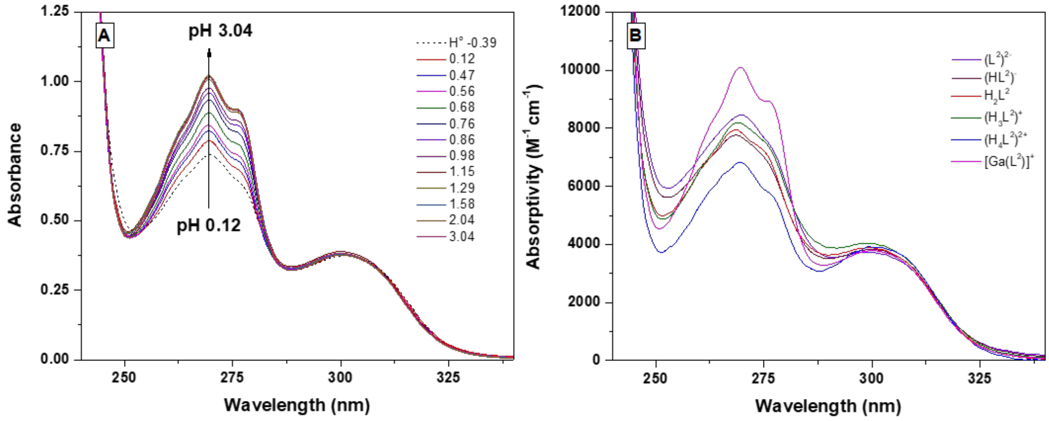

Figure S53. A) Spectra of the Ga^{3+}/H_2L^2 UV-acidic batch titration; $[Ga^{3+}] = [H_2L^2] = 1 \times 10^{-4}$ M, at 25 °C, l = 1 cm and *I* = 0.16 M NaCl (when possible). B) Molar absorptivities of H_2L^2 ligand and the $[Ga(L^2)]^+$ metal complex.

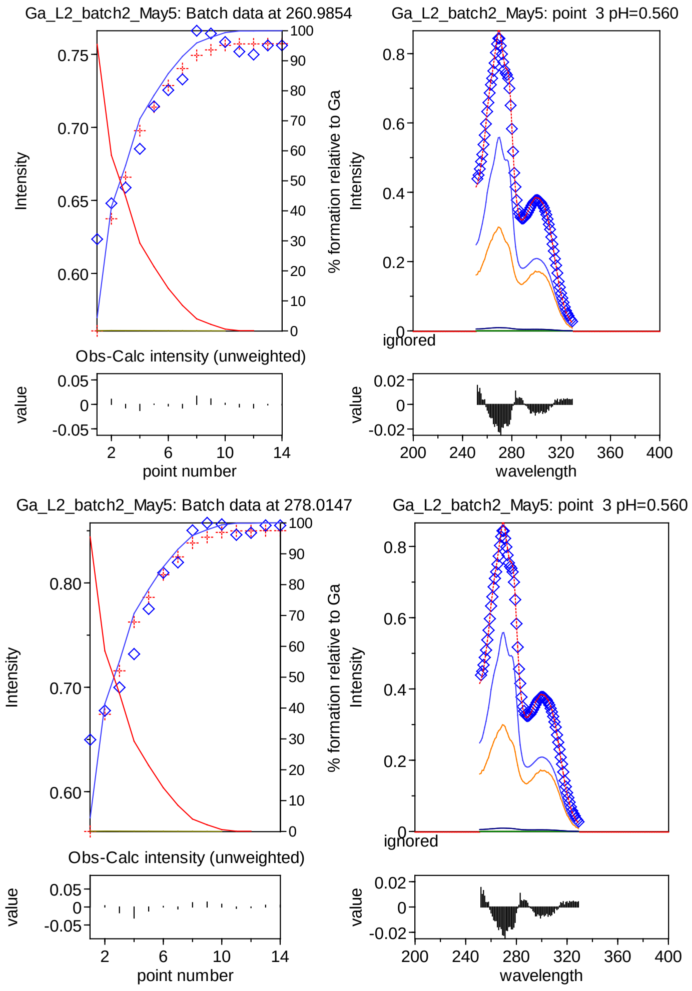

Figure S54. Fitting of the UV-acidic batch experiments (λ = 261 (top) and 278 nm (bottom)) of the Ga^{3+}-H_2L^2 system; $[Ga^{3+}] = [H_2L^2] = 1 \times 10^{-4}$ M at various pH values at 25 °C, *I* = 0.16 M NaCl, and l = 0.2 cm (left). Fitted spectra of the Ga^{3+}- H_2L^2 UV-acidic sample (pH = 0.56) considering the molar absorptivities of the free ligand (H_2L^2). The graphs on the left contain the calculated speciation plot as a function of gallium(III) concentration.

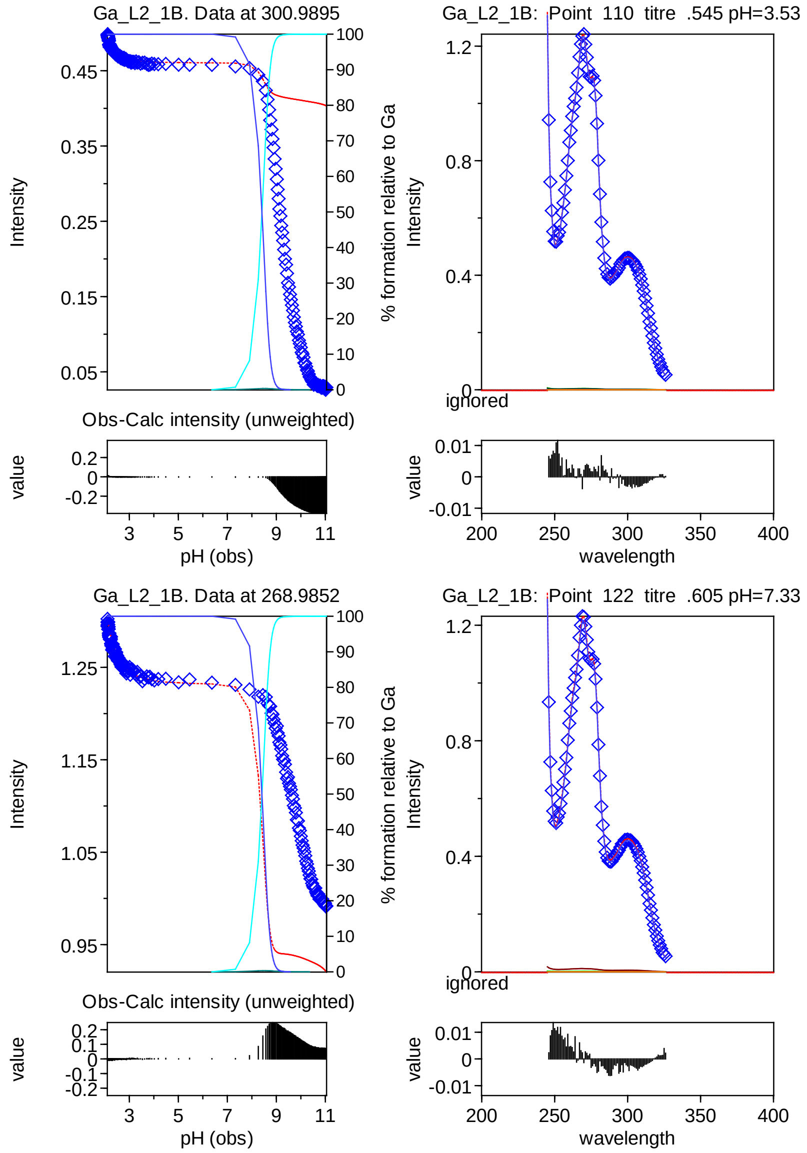

Figure S55. Fitting of the UV-potentiometric titration experiments (λ = 301 (top) and 269 nm (bottom)) of the Ga^{3+}-H_2L^2 system; [Ga^{3+}] = [H_2L^2] = 5.99 × 10^{-4} M at 25 °C, I = 0.16 M NaCl and l = 0.2 cm (left). Fitted spectra of the Ga^{3+}- H_2L^2 samples (pH = 3.53 and 7.33) considering the molar absorptivities of the free ligand (H_2L^2). The graph on the left contains the calculated speciation plot as a function of gallium(III) concentration. Graphs on the top and bottom left at pH > 7.9 show that the fitting of the UV-potentiometric titration is not possible due to the partial hydrolysis of the ligand, however as shown in the NMR section, the coordination of the Ga(III) ion is maintained as the $[GaL^3]^-$ species.

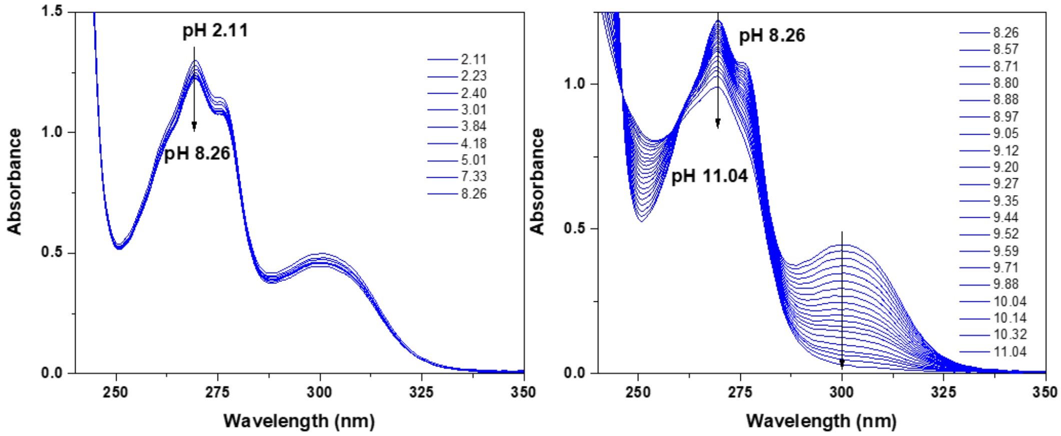

Figure S55 (continuation). Representative spectra of the UV-potentiometric titration of the Ga^{3+}-H_2L^2 system; $[Ga^{3+}] = [H_2L^2] = 5.99 \times 10^{-4}$ M at 25 °C, I = 0.16 M NaCl and l = 0.2 cm.

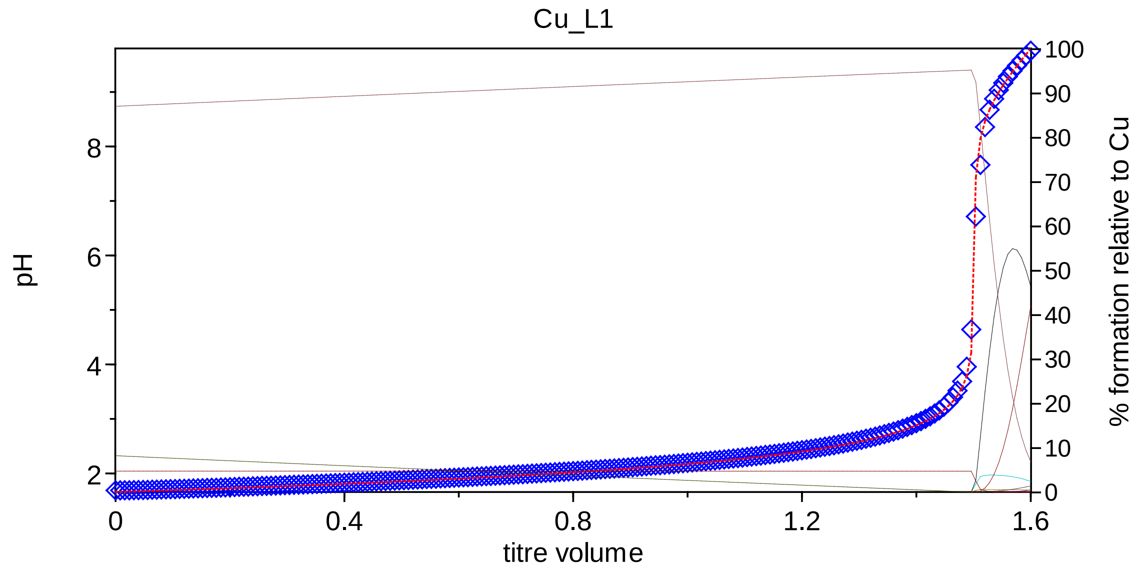

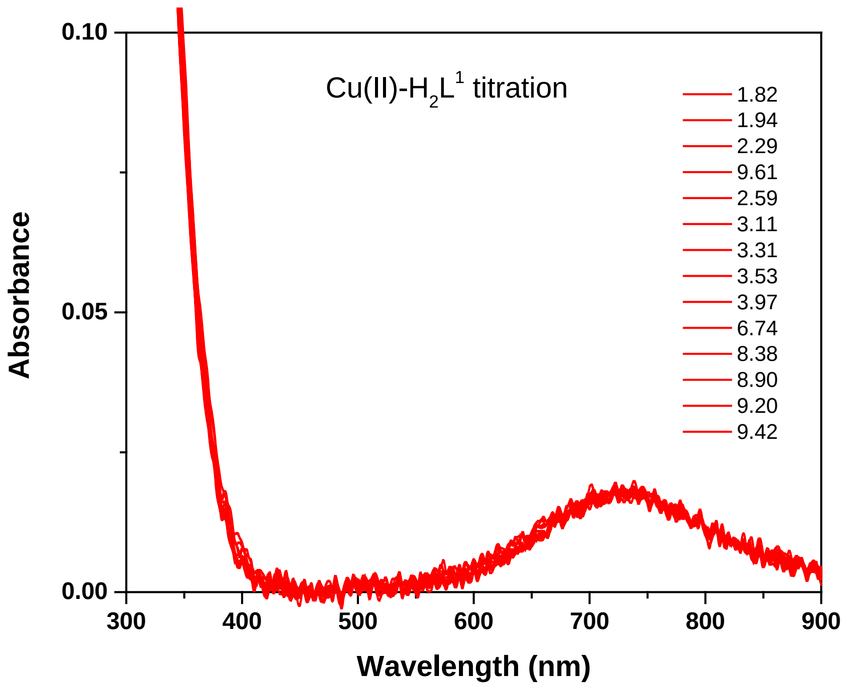

Figure S56. Potentiometric titration plots of aqueous solutions containing H_2L^1 and Cu^{2+}; $[H_2L^1] = [Cu^{2+}] = 8.99 \times 10^{-4}$ M, at 25 °C and I = 0.16 M (NaCl). Blue dots are experimental data points and red dotted line is the fitting using stability constants in Table 3 and Table S3. Selected UV spectra (bottom) of the Cu(II)-H_2L^1 potentiometric titration showing that the complexation of Cu(II) is maintained throughout the titration (pH 1.82-9.42, l = 0.2 cm).

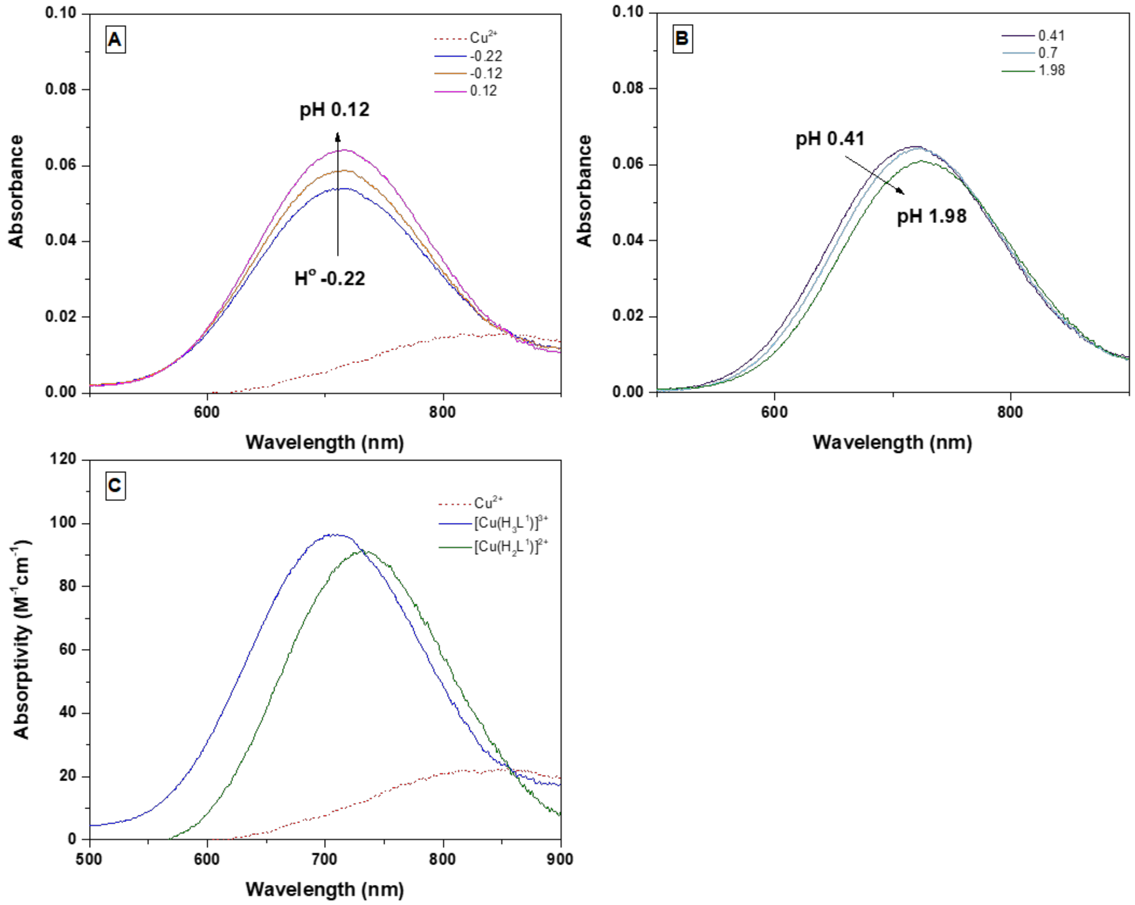

Figure S57. A) and B) Selected spectra of the Cu^{2+}/H_2L^1 UV-acidic batch titration; $[Cu^{2+}] = [H_2L^1] = 6.37 \times 10^{-4}$ M, at 25 °C, l = 1 cm and I = 0.16 M NaCl (when possible). C) Molar absorptivity of the Cu^{2+} complexes with H_2L^1 ligand calculated with stability constants in Table 3 and Table S3 using the HypSpec2014 software[2].

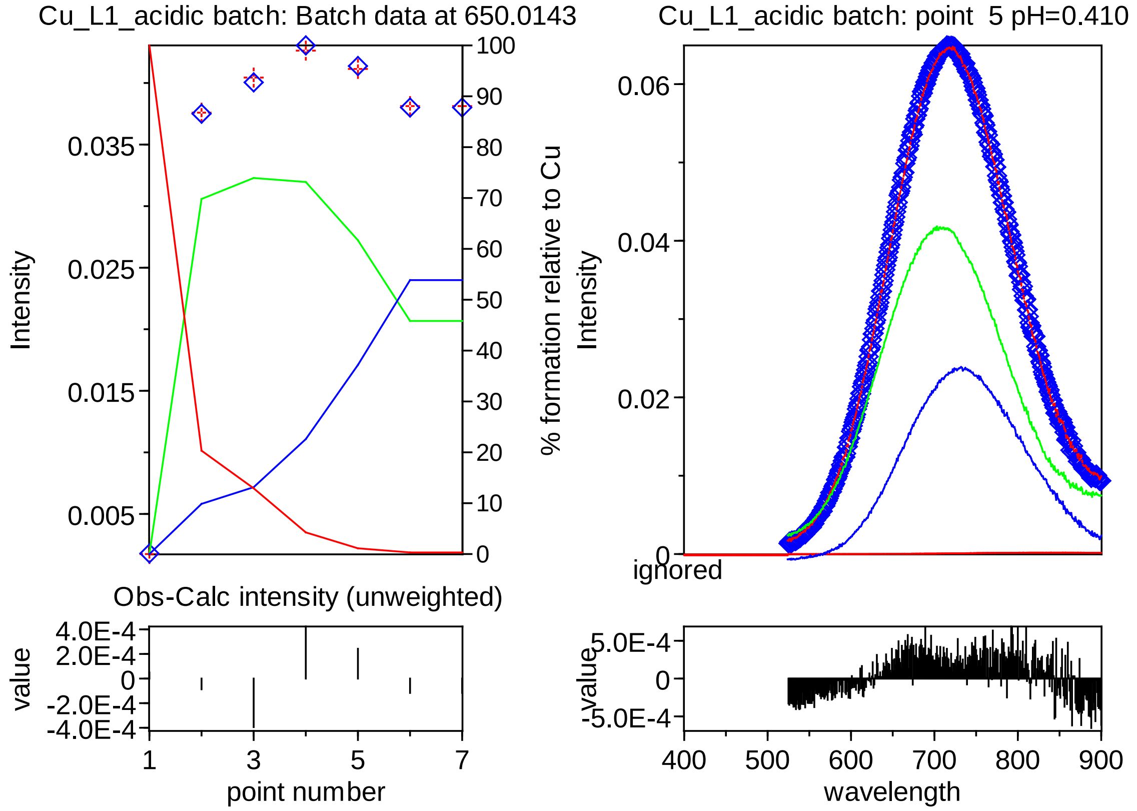

Figure S58. Fitting of the UV-acidic batch experiments (λ = 650 nm) of the Cu^{2+}-H_2L^1 system; [Cu^{2+}] = [H_2L^1] = 6.37 × 10^{-4} M at various pH values at 25 °C and *I* = 0.16 M NaCl (when possible) (left). Fitted spectrum of the Cu^{2+}- H_2L^1 UV-acidic sample (pH = 0.41) considering the molar absorptivities of the Cu^{2+} species at pH 0. The graph on the left contains the calculated speciation plot as a function of copper(II) concentration.

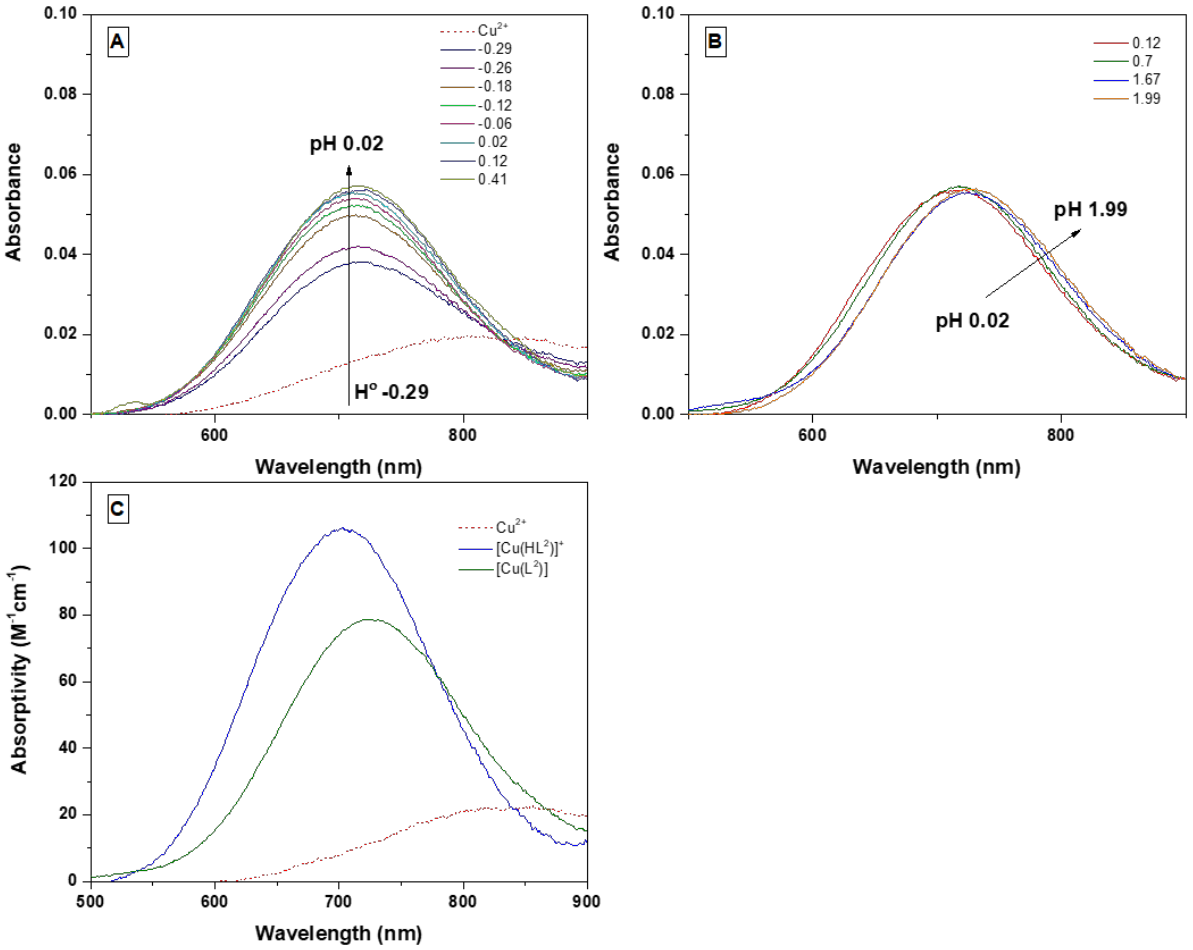

Figure S59. A) and B) Selected spectra of the Cu^{2+}/H_2L^2 UV-acidic batch titration $[Cu^{2+}] = [H_2L^2] = 7 \times 10^{-4}$ M, at 25 °C, l = 1 cm and I = 0.16 M NaCl (when possible). C) Molar absorptivity of the Cu^{2+} complexes with H_2L^2 ligand calculated with stability constants in Table 3 and Table S3 using the HypSpec2014 software[2].

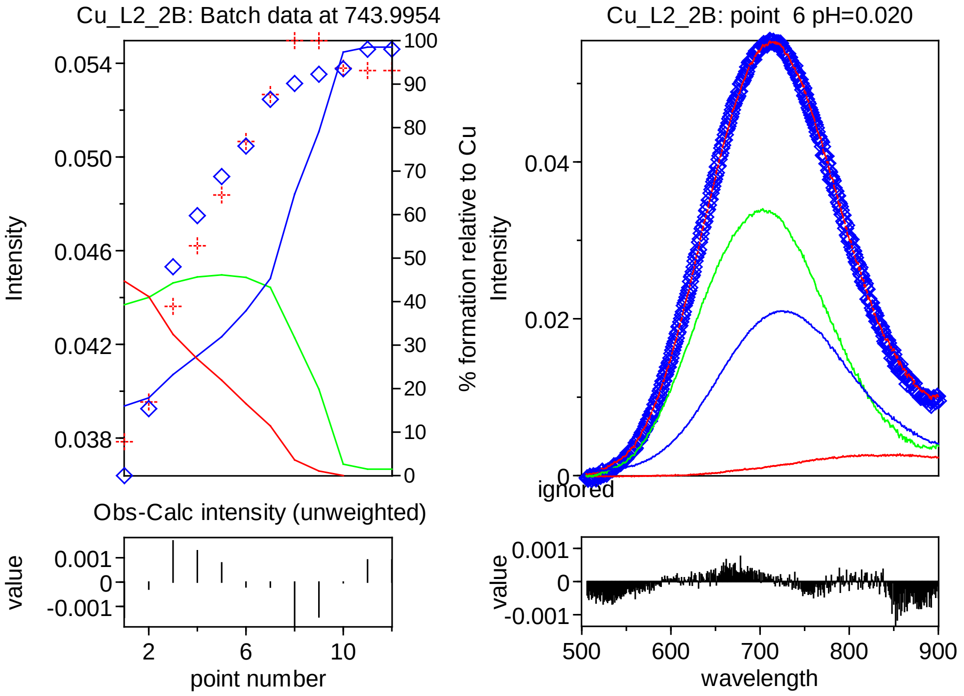

Figure S60. Fitting of the UV-acidic batch experiments (λ = 744 nm) of the Cu^{2+}-H_2L^2 system; $[Cu^{2+}] = [H_2L^2] = 7 \times 10^{-4}$ M at various pH values at 25 °C and I = 0.16 M NaCl (when possible) (left). Fitted spectrum of the Cu^{2+}- H_2L^2 UV-acidic sample (pH = 0.02) considering the molar absorptivities of the Cu^{2+} species at pH 0. The graph on the left contains the calculated speciation plot as a function of copper(II) concentration.

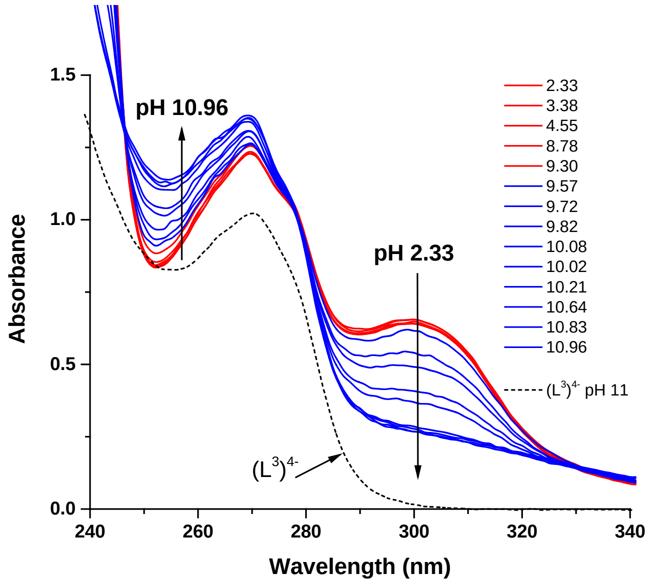

Figure S61. Selected UV spectra from combined potentiometric-spectrophotometric titrations of the Cu^{2+}-H_2L^2 system; $[Cu^{2+}] = [H_2L^2] = 5.96 \times 10^{-4}$ M at various pH values at 25 °C and I = 0.16 M NaCl, l = 0.2 cm. The decrease of the band at 300 nm from pH 9.57 marks the start of the formation of the $[CuL^3]^{2-}$ species. The spectra of the free $(L^3)^{4-}$ species is shown for reference.

Table S3. Formation constants of H_2L^1 and H_2L^2 with Cu^{2+} and Ga^{3+} metal ions[d].

H_2L^1			H_2L^2		
species	log β	pK	species	log β	pK
$[Cu(H_3L^1)]^{3+}$	42.50(2)[b]	0.63			
$[Cu(H_2L^1)]^{2+}$	41.87(2)[a]	8.98			
$[Cu(HL^1)]^{+}$	32.89(3)[a]	9.84	$[Cu(HL^2)]^{+}$	22.80(1)[b]	0.10
$[Cu(L^1)]$	23.05(2)[a]		$[Cu(L^2)]$	22.70(2)[b]	
**pCu**[c]	**21.96**		**pCu**	**22.8**	
$[Ga(H_2L^1)]^{3+}$	39.10(2)[b]	7.65;7.65			
$[Ga(HL^1)]^{2+}$	-				
$[Ga(L^1)]^{+}$	23.80(2)[a]		$[Ga(L^2)]^{+}$	20.69(1)[b]	
**pGa**[c]	**19.5**		**pGa**	**20.8**	

[a] Values obtained from UV-potentiometric titrations (25 °C, I = 0.16 M NaCl). log β_{pqr} values refer to the overall equilibria pM + qH + rL ↔ $M_pH_qL_r$. [b] Values obtained from UV-acidic batch titrations (25 °C, I = 0.16 M NaCl, when possible). [c] pM defined as –log[M_{free}] when [L] = 10 μM; [M] = 1 μM at pH 7.4. [d] Hydrolysis constants of metal ions used in the refinement at 25 °C and 0.16 M ionic strength: Ga(III) (log K $GaOH^{2+}$ = -3.12; log K $Ga(OH)_2^+$ = -6.01; log K $Ga(OH)_3$ = -10.44; log K $Ga(OH)_4^-$ = -16.7); Cu(II) (log K $CuOH^+$ = -8.25; log K $Cu(OH)_2$ = -17.56; log K $Cu(OH)_3^-$ = -27.81; log K $Cu(OH)_4^{2-}$ = -39.04; log K $Cu_2(OH)_2^{2+}$ = -10.64).

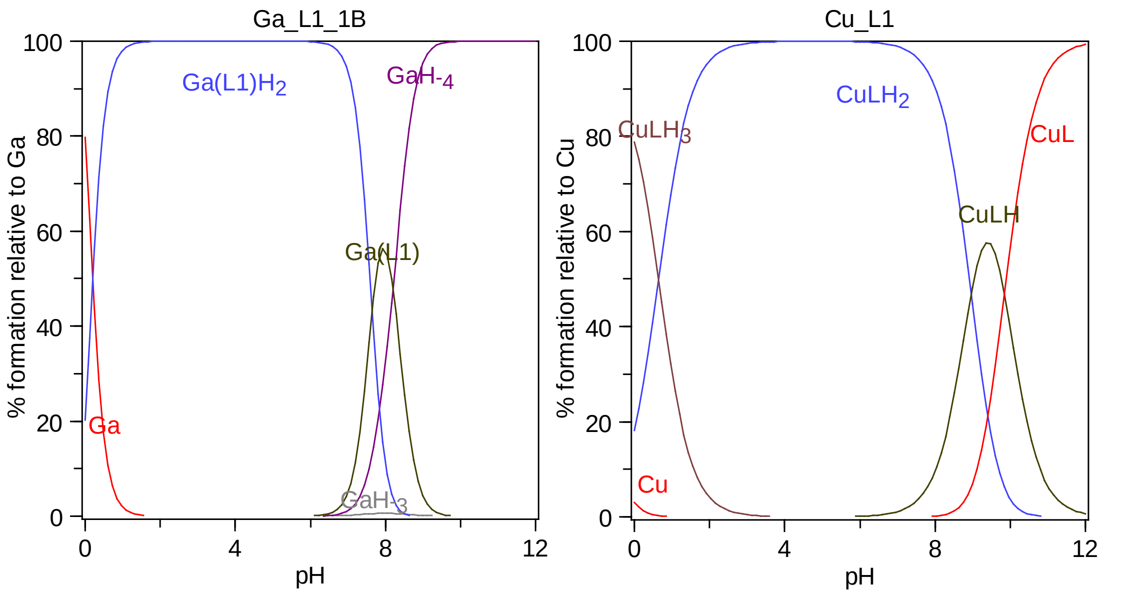

Figure S62. Speciation plot of H_2L^1 with Ga(III) (left) and Cu(II) (right) ([H_2L^1] = [Ga(III)] = [Cu(II)] = 0.001 M) calculated with protonation constants in Table 3 and stability constants in Table S3 using the Hyss software.[3] Metal hydrolysis constants were taken from the literature.[4]

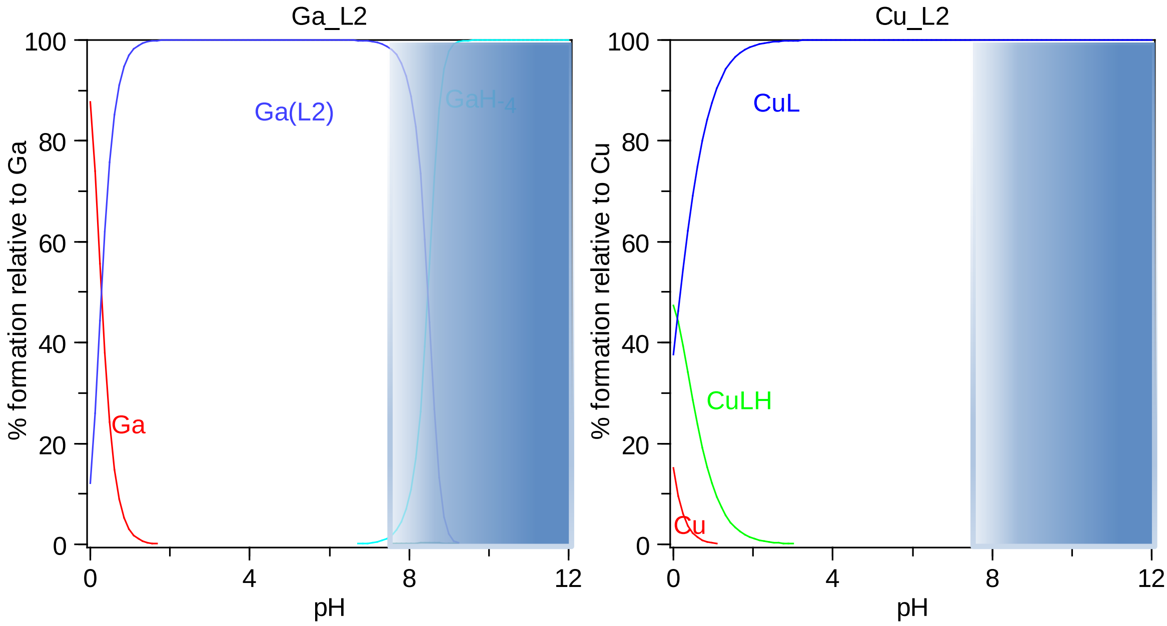

Figure S63. Speciation plot of H_2L^2 with Ga(III) (left) and Cu(II) (right) ($[H_2L^2]$ = [Ga(III)] = [Cu(II)] = 0.001 M) calculated with protonation constants in Table 3 and stability constants in Table S3. Metal hydrolysis constants were taken from the literature.[4] The gradient filled areas of both graphs represent the unknown speciation as the ligand H_2L^2 hydrolyses and it is not possible to fit the experimental data after pH 7.9. Nonetheless, there is evidence that the coordination of Cu(II) is maintained throughout the pH range 2-11 (Figure S61) and for Ga(III) up to at least pH 9, where the $[GaL^3]^-$ species start to decomplex (see NMR section).

Table S4. Protonation constants of discussed ligands.

Equilibrium Reaction	H_2L^{1a}	H_2L^{2a}	H_2dedpa	NOTA[e]	DOTA[f]
$L + H^+ \leftrightharpoons HL$	10.86 (1)	8.14 (1)	9.00 (3)[c]; 8.69 (1)[d]	10.773(7)	12.09 (4)
$HL + H^+ \leftrightharpoons H_2L$	9.91 (1)	4.98 (1)	6.31 (5)[c]; 6.18 (2)[d]	6.032(4)	9.680 (1)
$H_2L + H^+ \leftrightharpoons H_3L$	6.78 (1)	3.22 (1)	3.04 (6)[c]; 3.08 (2)[d]	3.163(3)	4.548 (3)
$H_3L + H^+ \leftrightharpoons H_4L$	4.23 (1)	2.14 (4)	2.59 (6)[c]; 2.33 (3)[d]	1.955(3)	4.130 (3)
$H_4L + H^+ \leftrightharpoons H_5L$	2.79 (1)				
$H_5L + H^+ \leftrightharpoons H_6L$	2.01 (6)				
$H_6L + H^+ \leftrightharpoons H_7L$	0.37 (2)[b]				
$\Sigma \log K_{HqL}$	36.95	18.48	20.94[c]; 20.28[d]	21.923	30.448

[a] Values were obtained from UV-potentiometric titrations (25 °C, l = 0.2 cm, I = 0.16 M NaCl). K_{HqL} defined as $[H_qL]/([H][H_{q-1}L])$; [b] From acidic in-batch UV titrations (25 °C, l = 1 cm, I = 0.16 M NaCl (when possible)); [c] From ref.[5]; [d] From ref.[6]; [e] From ref.[7]; [f] From ref.[8]; Charges omitted for clarity.

Correction to the $[Ga(dedpa)]^{+}$ Stability Constant (log K_{ML} = 28.11(8))

We noticed that the $[Ga(dedpa)]^{+}$ stability constant (log K_{ML} = 28.11(8)) reported in 2010 is very high.[5] It was calculated through ligand-ligand competition using EDTA as ligand competitor but this method of stability constant determination should be accompanied by a supporting spectroscopic technique such as ^{1}H NMR, UV-vis or in the case of Ga(III) ion, a simple potentiometric determination in competition with the $[Ga(OH)_4]^{-}$ ion at basic pH.

Only now that we intensively studied this series of H_2dedpa derivatives, we have discovered that the reported stability constant is too high compared to our results for H_2L^1 and H_2L^2. As the solution studies reported herein are a combination of multiple techniques, we were prompted to review the old data to investigate the validity of the data analysis. Figure S64, shows a potentiometric titration curve of H_2dedpa with Ga(III) at 1:1 molar ratio that was not included in the original paper and as mentioned above, it could have been fitted using the $[Ga(OH)_4]^{-}$ species as a competitor at basic pH. It is evident that log K_{ML} = 28.11(1) is too high as the fitted curve lies above the experimental curve. The correct fitted value log K_{ML} = 22.9(1) was based on a two replicate data set and it is in fact closer to our results for H_2L^1 and H_2L^2.

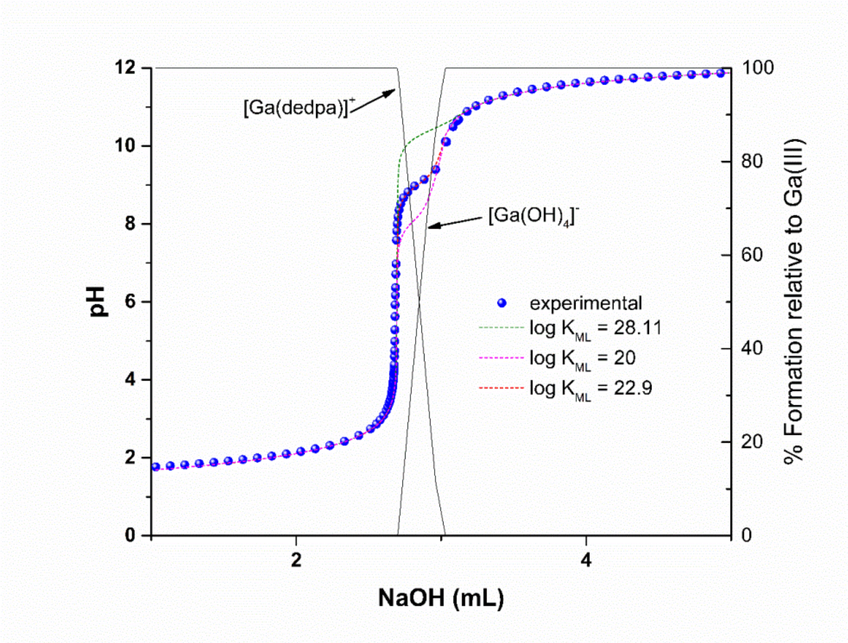

Figure S64. Potentiometric curve of an aqueous solution containing Ga(III)-H_2dedpa. $[Ga^{3+}]$ = $[H_2dedpa]$ = 9.86×10^{-4} M, I = 0.15 M NaCl at 25 °C. Green line is the fitting using the previously reported log K_{ML} = 28.11. Pink line is the fitting using log K_{ML} = 20 and red line is the fitting using log K_{ML} = 22.9. The graph also contains the speciation plot calculated with the corrected stability constant log K_{ML} = 22.9 and it shows how the potentiometric curve can be fitted based on the $[Ga(OH)_4]^{-}$ competition at pH~10.

Proton Assisted Dissociation Kinetics

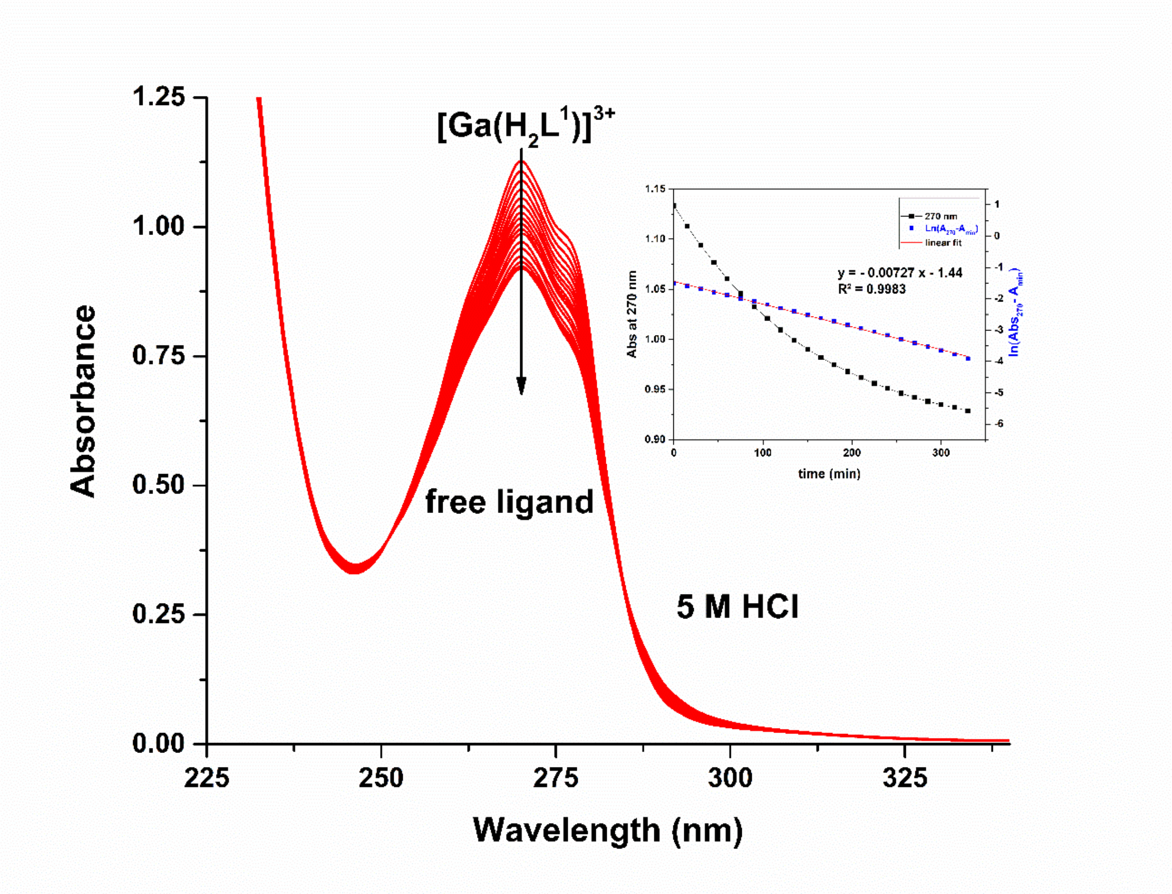

Figure S65. Time course of a UV spectra of a solution containing $[Ga]^{3+}$= $[H_2L^1]$ = 1 × 10^{-4} M ([HCl] = 5 M) at 25 °C, l = 1 cm. Spectra obtained at 15 min intervals. Inlet graph is the plot of the absorbance at 270 nm versus time and fit to obtain the first order rate constant and a $t_{1/2}$ = 1.6 h.

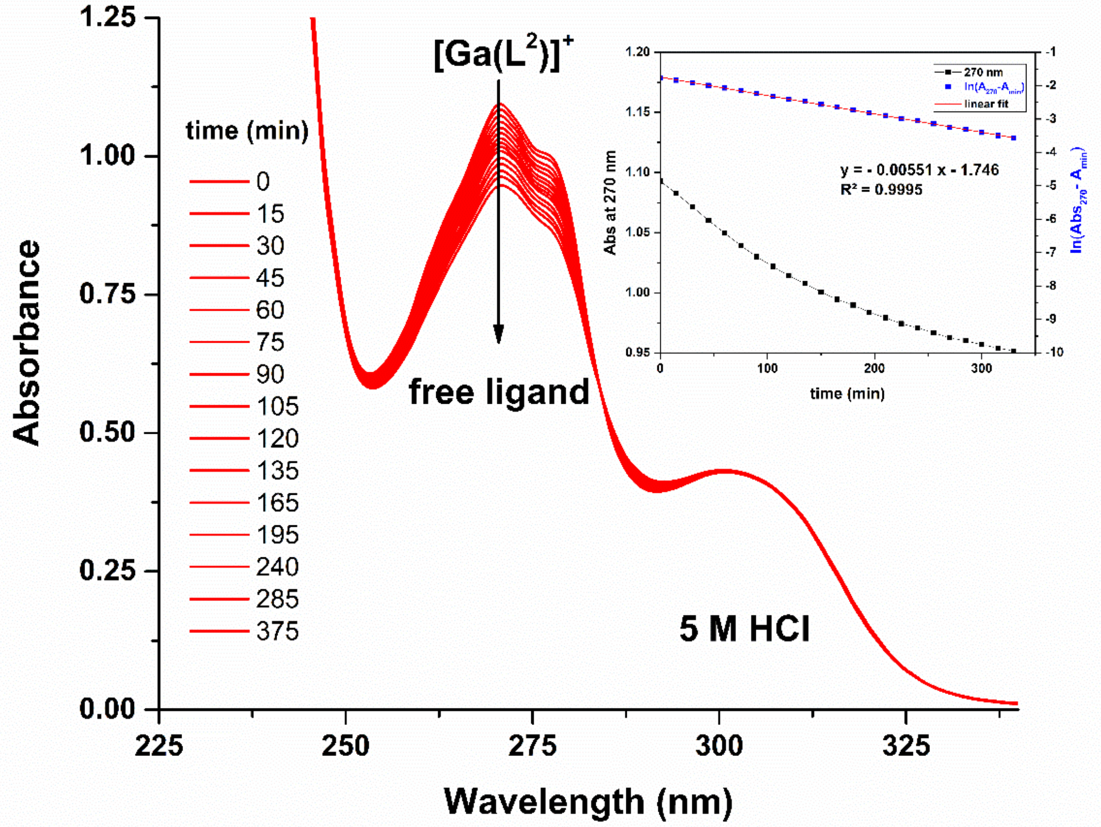

Figure S66. Time course of a UV spectra of a solution containing $[Ga]^{3+}$= $[H_2L^2]$ = 1 × 10^{-4} M ([HCl] = 5 M) at 25 °C, l = 1 cm. Spectra obtained at 15 min intervals. Inlet graph is the plot of the absorbance at 270 nm versus time and fit to obtain the first order rate constant and a $t_{1/2}$ = 2.1 h.

^{68}Ga and ^{64}Cu Radiolabeling Data

Concentration Dependent Radiolabeling

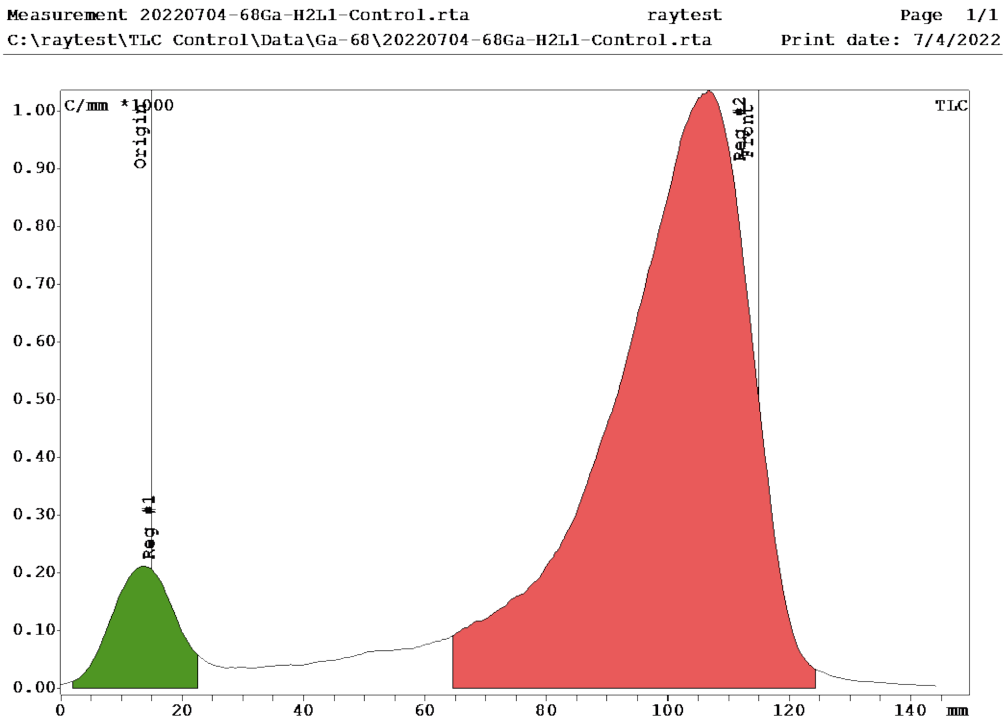

Figure S67. Representative radio thin-layer chromatogram of control ([^{68}Ga]GaCl$_3$) for [^{68}Ga][Ga(H$_2$L^1)]$^{3+}$ and [^{68}Ga][GaL^2]$^+$ concentration dependent radiolabeling. Reaction spotted on iTLC-SG paper plates with EDTA (50 mM, pH 5.5) as eluent.

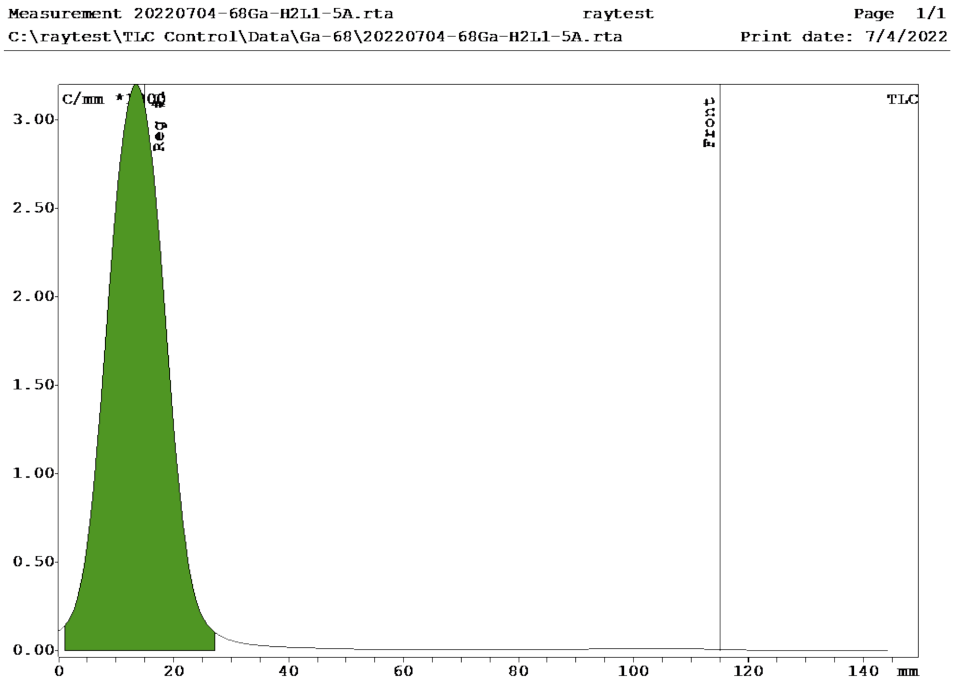

Figure S68. Representative radio thin-layer chromatogram of [^{68}Ga][Ga(H_2L^1)]$^{3+}$ concentration dependent radiolabeling ([H_2L^1] = 1 × 10^{-5} M, NaOAc buffer (2 M, pH 7.4), RT, 15 min). Reaction spotted on iTLC-SG paper plates with EDTA (50 mM, pH 5.5) as eluent.

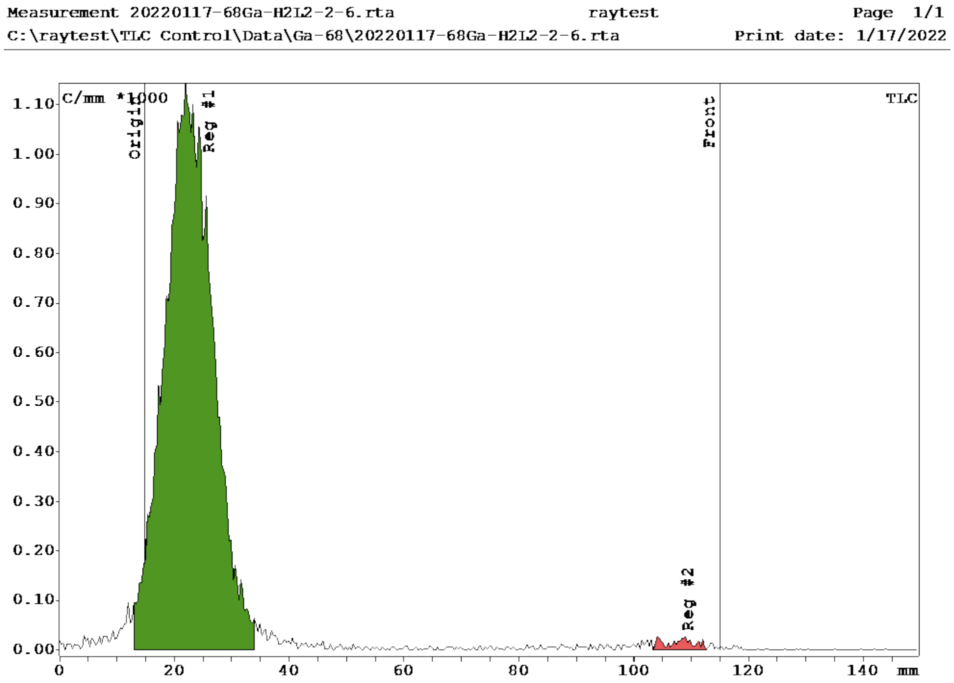

Figure S69. Representative radio thin-layer chromatogram of [^{68}Ga][GaL^2]$^{+}$ concentration dependent radiolabeling ([H_2L^2] = 1 × 10^{-6} M, NaOAc buffer (2 M, pH 7.4), RT, 15 min). Reaction spotted on iTLC-SG paper plates with EDTA (50 mM, pH 5.5) as eluent.

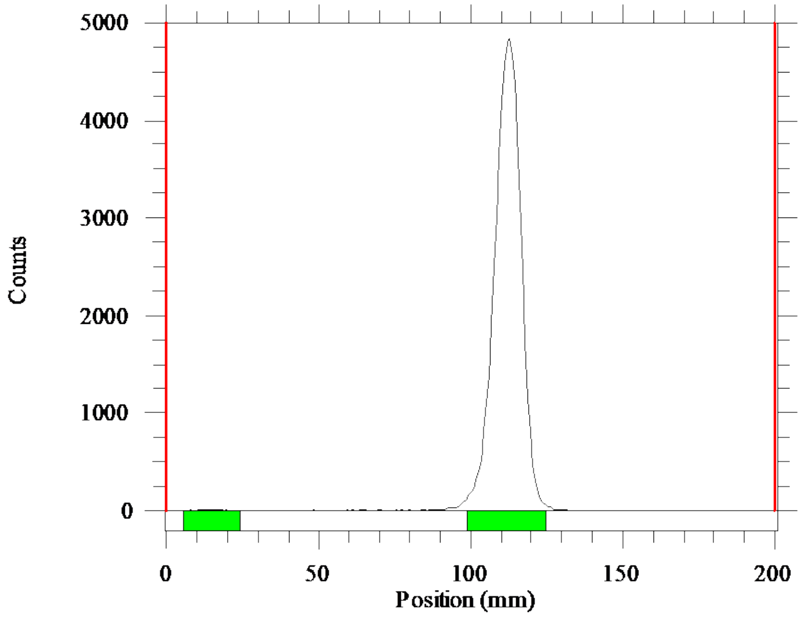

Figure S70. Representative radio thin-layer chromatogram of control ([^{64}Cu]$CuCl_2$) for [^{64}Cu][Cu(H_2L^1)]$^{2+}$ and [^{64}Cu][CuL^2] concentration dependent radiolabeling. Reaction spotted on iTLC-SA paper plates with EDTA (100 mM, pH 5) as eluent

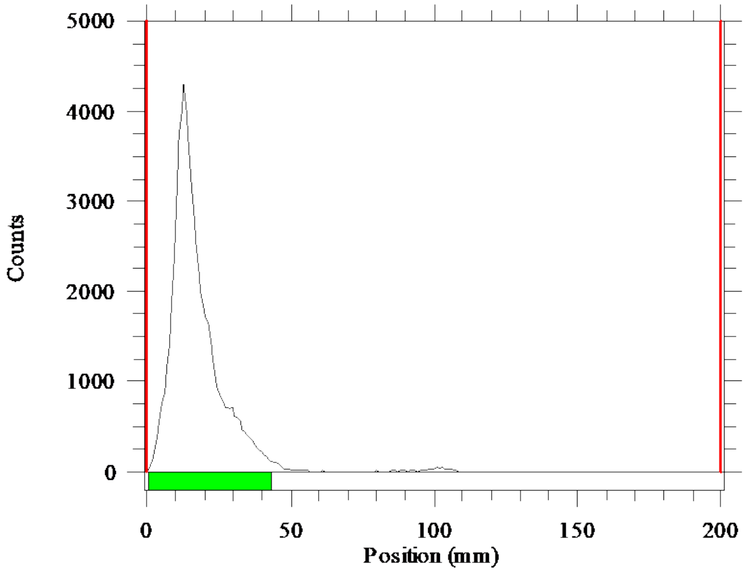

Figure S71. Representative radio thin-layer chromatogram of [^{64}Cu][Cu(H_2L^1)]$^{2+}$ concentration dependent radiolabeling ([H_2L^1] = 1×10^{-5} M, NaOAc buffer (0.5 M, pH 7), RT, 15 min). Reaction spotted on iTLC-SA paper plates with EDTA (50 mM, pH 5) as eluent.

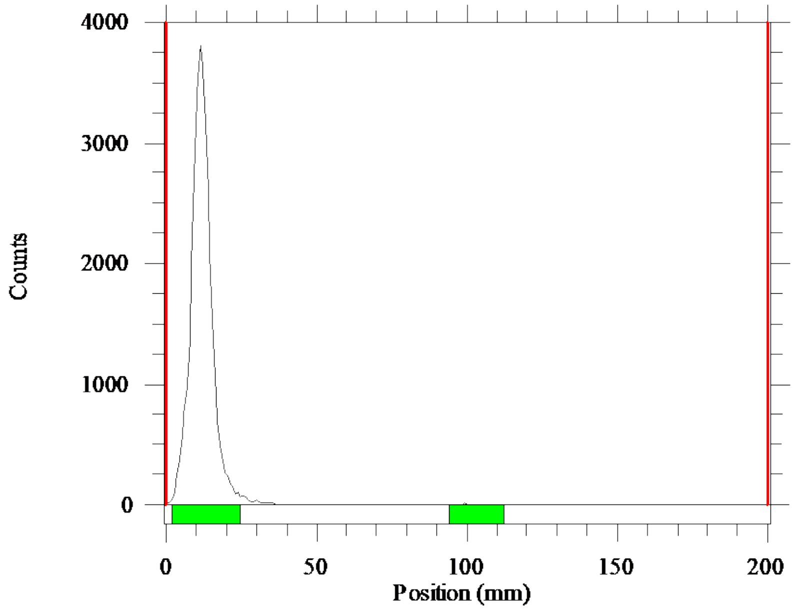

Figure S72. Representative radio thin-layer chromatogram of [^{64}Cu][CuL^2] concentration dependent radiolabeling ([H_2L^2] = 1 × 10^{-5} M, NaOAc buffer (0.5 M, pH 7), RT, 15 min). Reaction spotted on iTLC-SA paper plates with EDTA (50 mM, pH 5) as eluent.

Table S5. pH dependent [^{68}Ga]$GaCl_3$ radiolabeling (n = 1) with H_2L^1 and H_2L^2 ligands at 1 × 10^{-4} M final ligand concentration.

pH	H_2L^1 RCC (%)	H_2L^2 RCC (%)
**4**	100	100
**6**	100	99
**7.4**	100	98

2 M NaOAc buffer, RT, 7.4 MBq of [^{68}Ga]$GaCl_3$.

Table S6. pH dependent [^{64}Cu]$CuCl_2$ radiolabeling (n = 1) with H_2L^1 and H_2L^2 ligands at 1 × 10^{-4} M final ligand concentration.

pH	H_2L^1 RCC (%)	H_2L^2 RCC (%)
**4**	100	100
**5**	100	100
**7**	100	100

0.5 M NaOAc buffer, RT, 0.66 MBq of [^{64}Cu]$CuCl_2$.

Human Serum Stability Data

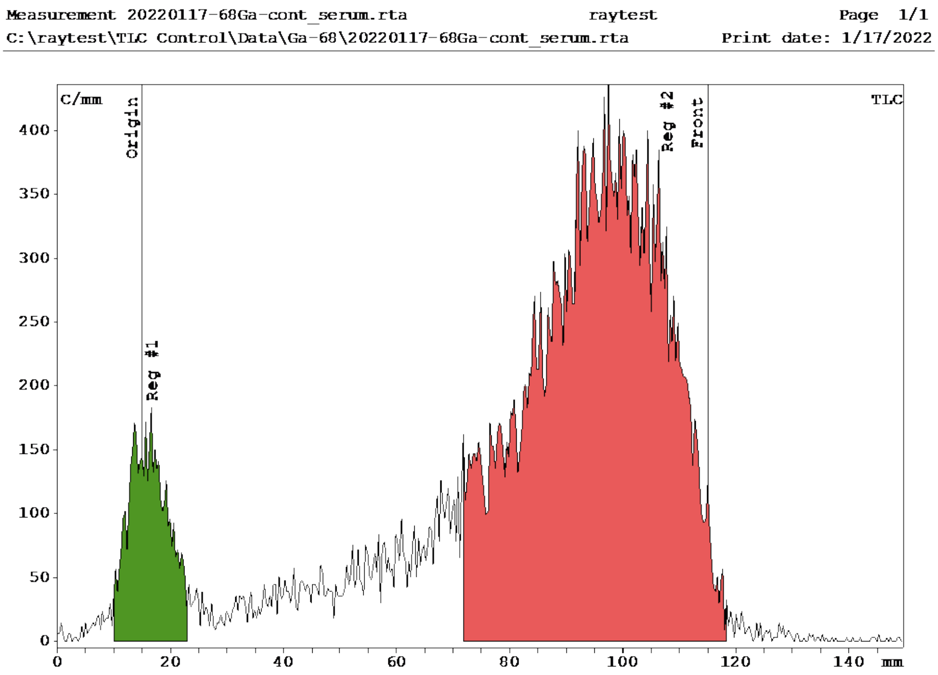

Figure S73. Representative radio thin-layer chromatogram of control ([^{68}Ga]$GaCl_3$) for [^{68}Ga][Ga(H_2L^1)]$^{3+}$ and [^{68}Ga][GaL^2]$^+$ human serum stability assays. Reaction spotted on iTLC-SG paper plates with EDTA (50 mM, pH 5.5) as eluent.

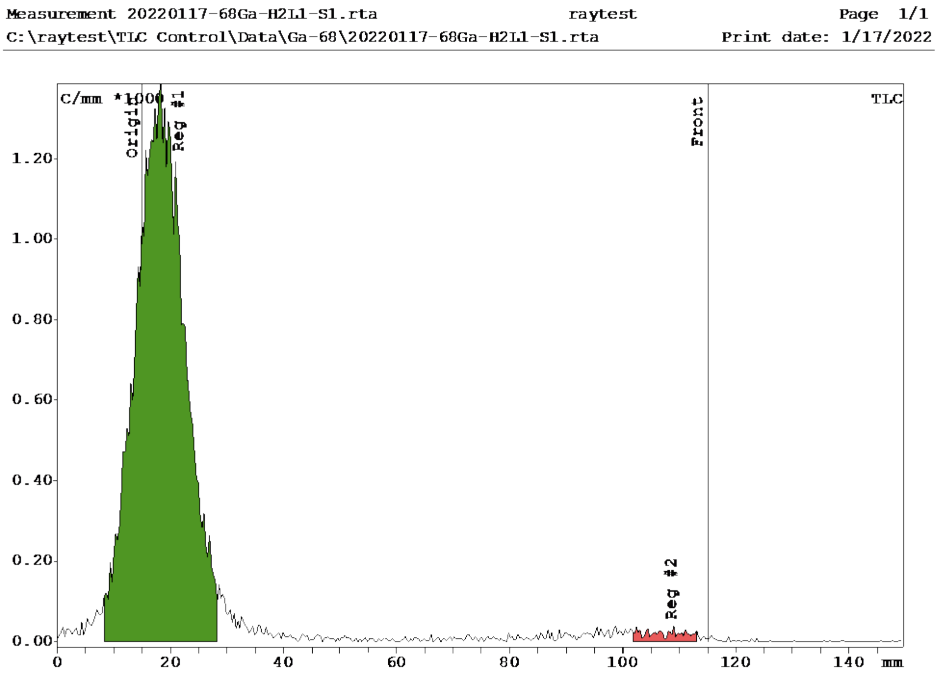

Figure S74. Representative radio thin-layer chromatogram of [^{68}Ga][Ga(H_2L^1)]$^{3+}$ in human serum after 1 h incubation time. Reaction spotted on iTLC-SG paper plates with EDTA (50 mM, pH 5.5) as eluent.

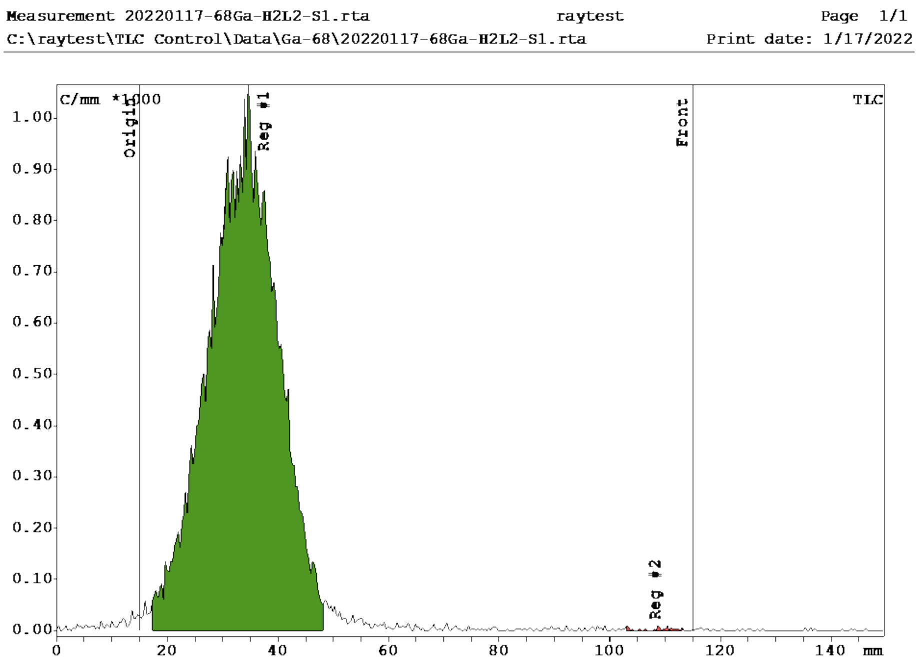

Figure S75. Representative radio thin-layer chromatogram of $[^{68}Ga][GaL^2]^+$ in human serum after 1 h incubation time. Reaction spotted on iTLC-SG paper plates with EDTA (50 mM, pH 5.5) as eluent.

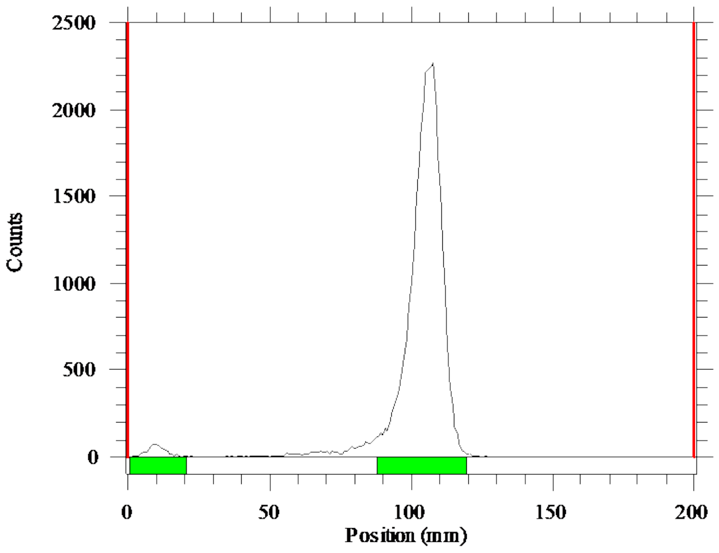

Figure S76. Representative radio thin-layer chromatogram of control ($[^{64}Cu]CuCl_2$) for $[^{64}Cu][Cu(H_2L^1)]^{2+}$ and $[^{64}Cu][CuL_2]$ concentration dependent radiolabeling. Reaction spotted on iTLC-SA paper plates with EDTA (50 mM, pH 5) as eluent.

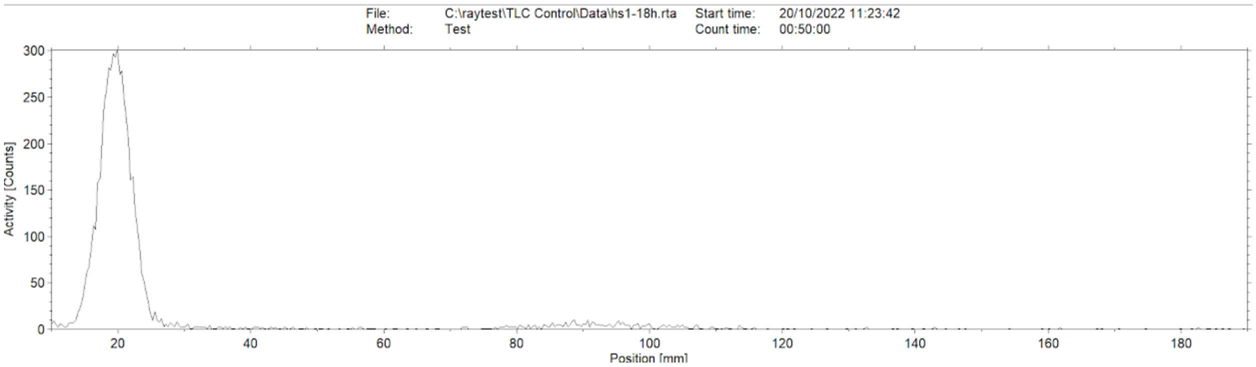

Figure S77. Representative radio thin-layer chromatogram of [^{64}Cu][Cu(H$_2$L^1)]$^{2+}$ in human serum after 18 h incubation time. Reaction spotted on iTLC-SA paper plates with EDTA (50 mM, pH 5) as eluent.

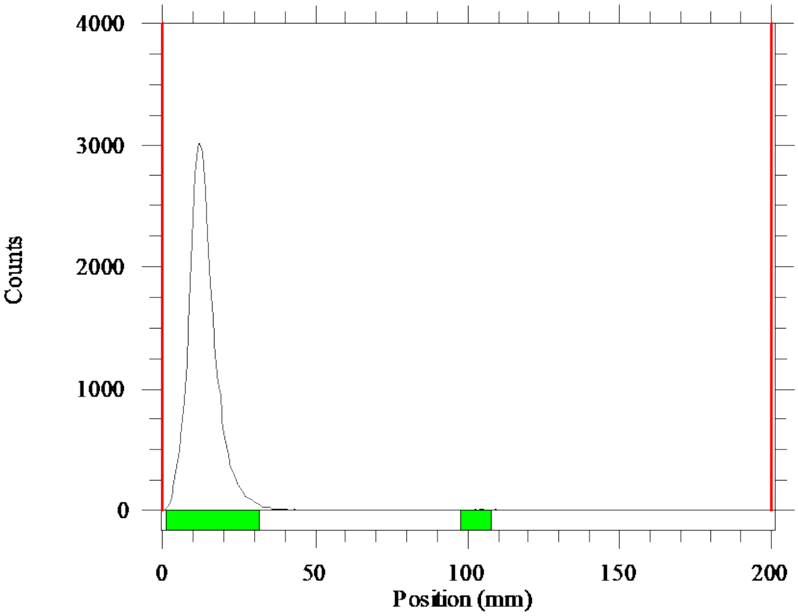

Figure S78. Representative radio thin-layer chromatogram of [^{64}Cu][CuL^2] in human serum after 18 h incubation time. Reaction spotted on iTLC-SA paper plates with EDTA (50 mM, pH 5) as eluent.

References

(1) Gans, P.; Sabatini, A.; Vacca, A. Investigation of Equilibria in Solution. Determination of Equilibrium Constants with the HYPERQUAD Suite of Programs. *Talanta* **1996**, *43*, 1739–1753.

(2) Gans, P.; Sabatini, A.; Vacca, A. Determination of Equilibrium Constants from Spectrophometric Data Obtained from Solutions of Known PH: The Program PHab. *Anal. Chim.* **1999**, *89*, 45–49.

(3) Alderighi, L.; Gans, P.; Ienco, A.; Peters, D.; Sabatini, A.; Vacca, A. Hyperquad Simulation and Speciation (HySS): A Utility Program for the Investigation of Equilibria Involving Soluble and Partially Soluble Species. *Coord. Chem. Rev.* **1999**, *184*, 311-318.

(4) Baes, C. F. Jr.; Mesmer, R. E. The Hydrolysis of Cations. In *The Hydrolysis of Cations*; John Wiley & Sons, New York: New York, 1976.

(5) Boros, E.; Ferreira, C. L.; Cawthray, J. F.; Price, E. W.; Patrick, B. O.; Wester, D. W.; Adam, M. J.; Orvig, C. Acyclic Chelate with Ideal Properties for ^{68}Ga PET Imaging Agent Elaboration. *J. Am. Chem. Soc.* **2010**, *132*, 15726–15733.

(6) Ferreirós-Martínez, R.; Esteban-Gómez, D.; Platas-Iglesias, C.; de Blas, A.; Rodríguez-Blas, T. Zn(II), Cd(II) and Pb(II) Complexation with Pyridinecarboxylate Containing Ligands. *Dalton Trans.* **2008**, 5754-5765.

(7) Bevilacqua, A.; Gelb, R. I.; Hebard, W. B.; Zompa, L. J. Equilibrium and Thermodynamic Study of the Aqueous Complexation of 1,4,7-Triazacyclononane-N,N',N''-Triacetic Acid with Protons, Alkaline-Earth-Metal Cations, and Copper(II). *Inorg. Chem.* **1987**, *26*, 2699–2706.

(8) Delgado, R.; da Silva, J. J. R. F. Metal Complexes of Cyclic Tetra-Azatetra-Acetic Acids. *Talanta* **1982**, *29*, 815–822.
